# Smartphone Psychological Therapy During COVID-19: A Study on the Effectiveness of Five Popular Mental Health Apps for Anxiety and Depression

**DOI:** 10.3389/fpsyg.2021.775775

**Published:** 2021-12-13

**Authors:** Jamie M. Marshall, Debra A. Dunstan, Warren Bartik

**Affiliations:** School of Psychology, Faculty of Medicine and Health, University of New England, Armidale, NSW, Australia

**Keywords:** mHealth, mental health apps, smartphones, COVID-19, anxiety, depression, single-case design

## Abstract

The aims of this study were to examine the effectiveness of a range of smartphone apps for managing symptoms of anxiety and depression and to assess the utility of a single-case research design for enhancing the evidence base for this mode of treatment delivery. The study was serendipitously impacted by the COVID-19 pandemic, which allowed for effectiveness to be additionally observed in the context of significant community distress. A pilot study was initially conducted using the*SuperBetter* app to evaluate the proposed methodology, which proved successful with the four finishing participants. In the main study, 39 participants commenced (27 females and 12 males,*M_Age_* = 34.04 years,*SD* = 12.20), with 29 finishing the intervention phase and completing post-intervention measures. At 6-month follow-up, a further three participants could not be contacted. This study used a digitally enhanced, multiple baseline across-individuals single-case research design. Participants were randomly assigned to the following apps:*SuperBetter* (*n* = 8),*Smiling Mind* (*n* = 7),*MoodMission* (*n* = 8),*MindShift* (*n* = 8), and*Destressify* (*n* = 8). Symptomatology and life functioning were measured at five different time points: pre-baseline/screening, baseline, intervention, 3-week post-intervention, and 6-month follow-up. Detailed individual perceptions and subjective ratings of the apps were also obtained from participants following the study’s completion. Data were analyzed using visual inspection, time-series analysis, and methods of statistical and clinical significance. Positive results were observed for all apps. Overall, more favorable outcomes were achieved by younger participants, those concurrently undertaking psychotherapy and/or psychotropic medication, those with anxiety and mixed anxiety and depression rather than stand-alone depression, and those with a shorter history of mental illness. Outcomes were generally maintained at 6-month follow-up. It was concluded that a diverse range of evidence-based therapies offered via apps can be effective in managing mental health and improving life functioning even during times of significant global unrest and, like all psychotherapies, are influenced by client features. Additionally, this single-case research design is a low-cost/high value means of assessing the effectiveness of mental health apps.

Clinical Trial Registration: The study is registered with the Australian and New Zealand Clinical Trials Registry (ANZCTR), which is a primary registry in the World Health Organization Registry Network, registration number ACTRN12619001302145p (http://www.ANZCTR.org.au/ACTRN12619001302145p.aspx).

## Introduction

Currently, there are over 10,000 mental health apps publicly available (Torous et al., 2018), but most of these have not been developed using established theoretical frameworks (Marshall et al., 2020a), or by recognized mental health experts (Shen et al., 2015; Alyami et al., 2017). Additionally, most of the comparatively few apps with research evidence of efficacy are not further supported by additional studies by researchers unaffiliated with the app or in diverse samples of participants (Marshall et al., 2019). In the interests of public safety and greater understanding of the usage of individual apps, more research needs to be carried out (Firth et al., 2017a,b; Lui et al., 2017). To achieve this, a low-cost/high-value research design is required (Clough and Casey, 2015a; Marshall et al., 2020b).

### Regulation and Risks of Mental Health Apps

Mental health apps have gone largely unregulated by government authorities in most parts of the world (Marshall et al., 2020d), but there is evidence that this is changing as it becomes apparent that regulatory oversight of mental health apps may improve the quality of the available apps. Increased regulation may assist app developers to create apps that use evidence-based, “best practice” principles and may also assist clinicians and consumers in choosing efficacious apps. The possible downside of regulation may be that smaller app developers with limited financial resources may not be able to afford to pay for their app to be regulated or “assessed,” and this in turn may lead to novel app interventions being restricted or blocked from being widely available.

One of the main issues at the heart of regulation concerns the risk of harm to consumers. Specifically, governments and health authorities need to be sure that, at the very least, a consumer will not be at risk of harm when using a mental health app. Without proper oversight, it is possible that an app may provide ill-advised advice to a consumer who may be experiencing significant mental health issues, such as suicidal ideation. If the wrong advice is given, or an inappropriate intervention is offered, the worst outcome could be harm or death to the user. Furthermore, regulation may be required to confirm than an app does what it says it does. For example, an app may claim to use interventions from a specific type of therapy (see next section), but if the interventions are not accurately based on such an evidence-based framework, it may result in the credibility of that framework being questioned by the user (Marshall et al., 2020a). More worryingly, if a user questioned such an evidence-based framework which was misinterpreted or misunderstood, the misinformation could easily and quickly be disseminated in online forums and social media platforms, possibly resulting in widespread unfair negative criticism being broadcast about that theoretical framework.

### Mental Health Apps as Mechanized Psychotherapy

Best practice for treating symptoms of anxiety and depression depends on the individual’s unique presentation and will involve evidence-based psychotherapy and/or antidepressant medication (American Psychiatric Association, 2009, 2010; Cuijpers et al., 2013). Widely used evidence-based psychotherapies include: cognitive-behavioral therapy (CBT; Beck et al., 1979; Butler et al., 2006; Bennett-Levy et al., 2009, 2010), interpersonal therapy (ITP; de Mello et al., 2005), dialectical behavior therapy (DBT; Lynch et al., 2006), acceptance and commitment therapy (ACT; Vollestad et al., 2012; A-Tjak et al., 2015), and positive psychology interventions (Seligman and Csikszentmihalyi, 2000; Seligman et al., 2005).

Research has shown that several factors influence the prognosis and outcomes of psychotherapy. These include client–therapist rapport (Tang and DeRubeis, 1999), client motivation (Addis and Jacobson, 2000), chronicity/history of mental illness (Hamilton and Dobson, 2002), functional impairment, social support, coping style, level of client resistance, subjective distress, and readiness to change (Beutler and Clarkin, 1990). Such factors combined with selected treatment may account for over 90% of the variance in successful outcomes (Beutler et al., 1999). In terms of appropriate treatment, while CBT is effective in treating depression, ITP may be more useful in circumstances where the precipitating factor is an interpersonal relationship issue (Zhou et al., 2017). Similarly, positive psychology approaches may be more applicable when highly motivated; older individuals wish to focus on strengths and positive interventions to maximize their psychological well-being (Sin and Lyubomirsky, 2009). In this way, positive psychology strategies may complement rather than replace traditional CBT approaches (Harvard Medical School, 2008). Overall, it is reasonable to assume that individual participant characteristics, the treatment approach, and the participant’s perceived engagement will influence clinical outcomes, including outcomes from treatments using a mental health app. It is these types of influences that are examined in effectiveness research (Singal et al., 2014).

### The Importance of Effectiveness Research

The current evidence base supporting the use of mental health apps for anxiety and depression includes a small number of studies of efficacy and even fewer of effectiveness. In clinical psychology, efficacy studies occur under controlled conditions where participants are screened for their suitability to improve the homogeneity of the experimental group, whereas effectiveness studies are designed to measure interventions in “real-world” clinical settings with more heterogeneous populations (Kazdin, 2017). An intervention that has been found to be efficacious also needs to demonstrate effectiveness in clinical practice (Singal et al., 2014). An efficacy trial may inflate an intervention’s clinical impact (effect size) in practice; therefore, it is important for treatments to have demonstrated effectiveness in this context. Although proven efficacy increases the chances of observing an intervention effect if one exists, effectiveness research accounts for individual clinician, client, and process characteristics that may moderate an intervention’s effect (Singal et al., 2014).

If the research on mental health apps is to be free of the limitations of inflated effect sizes found in efficacy studies, effectiveness studies are required. In a recent review of the two major app stores, only 3% of apps that claimed to offer a therapeutic treatment for anxiety and depression had published peer-reviewed research to back up their assertions (Marshall et al., 2019); thus, the majority of apps do not have the research evidence needed to inform individuals or clinicians (Firth et al., 2017a,b). If the proportion of research of both efficacy and effectiveness was increased, mental health apps could achieve widespread acceptance and validation by consumers and clinicians alike.

### Research on Mental Health Apps

There are several challenges to conducting traditional efficacy studies on mental health apps, and these are mainly due to the rapid pace of app development, the high cost of running large research trials, and the obsolescence of digital products – some mental health apps that go through a research process never become publicly available (Firth et al., 2017b). The app industry is populated by young start-up companies with large investment funds to produce the “next big thing” in health-related apps (Medical Startups, 2020) and bring it to market as soon as possible. As such, efficacy research using the traditional gold-standard randomized controlled trial (RCT) may be an impediment for mental health app research, given the long periods (sometimes years) to organize, run, and analyze a trial. During this time, other apps aimed at the same market may be listed for download, making the app going through this research process obsolete even before it has been publicly released (Clough and Casey, 2015a). Such costs on top of already large financial sums that have gone into the development of a product up to that point can be difficult for investors to accept. Thus, while RCTs are the “gold standard” for demonstrating efficacy, a different research approach may be required in the area of mental health apps (Clough and Casey, 2015a).

### Single-Case Designs

Single-case research designs are a viable alternative to large group designs, such as RCTs, and have the capacity to evaluate both the efficacy and effectiveness of mental health apps (Clough and Casey, 2015b; Mehrotra and Tripathi, 2018; Marshall et al., 2020d). This is because single-case research designs can assess the causal relationship between an intervention and outcomes (i.e., its efficacy), while also having the external validity to demonstrate effectiveness in heterogeneous samples (Lobo et al., 2017).

Single-case designs control for threats in internal validity by having continuous and repeated measurement of outcomes (dependent variables), random assignment, the potential for multiple participants, replication, and specific data analysis and statistics (Krasny-Pacini and Evans, 2018). With a baseline phase of “no treatment,” a participant acts as their own control through the sequential introduction of varying levels of an intervention (the independent variable) across “phases” of a study. In a design involving multiple participants, random assignment to the staggered introduction of the intervention addresses threats to internal validity from history, maturation and testing. In circumstances where three or more participants share similar presentations, receive identical treatment, and show strong outcomes, the results are considered to be a legitimate demonstration of efficacy (Horner et al., 2005; Barlow et al., 2009; Kazdin, 2017). By taking into account the features of individual participants, such designs also provide data on effectiveness (Buckley et al., 2014; Sheridan, 2014). See the Procedure section for details of the*multiple baseline across-individuals* design of the present study.

There have been calls for practicing clinicians to be more involved in the process of researching mental health interventions, especially those that are well-suited to being incorporated into real-world therapeutic settings, such as smartphone apps (Clough and Casey, 2015a). The use of single-case designs could facilitate the recruitment of practicing clinicians to research the efficacy and effectiveness of mental health apps by focusing on a limited number of participants from the clinician’s usual client load (Clough and Casey, 2015a; Marshall et al., 2020d). Marshall et al. (2020d) summarizes a model of how clinicians may contribute to a centralized database of efficacy and effectiveness information on mental health apps by following the design of the present study. Such a database would offer an ever-increasing knowledge hub that complements app review websites such as*PsyberGuide*^1^;*Head To Health*^2^; and the*NHS Apps Library*.^3^

### The Impact and Consequences of COVID-19

Soon after the present study commenced in early 2020, the COVID-19 pandemic began to have widespread negative impacts around the globe. It became clear that mental health was one such negative impact in countries, including Australia (Koh, 2020), China (Feng, 2020), India (Mukherjee, 2020), New Zealand (Mindfood, 2020), United Kingdom (Chowdhury, 2020), United States (Heilweil, 2020), and others. Due to the increased demand for services from mental health professionals, many people struggled to access in-person services and this led to increased demand for online/Telehealth options (Dunlop et al., 2019; Liu et al., 2020; Medhora, 2020; Marshall et al., 2020c). This included a 50% increase in the number of Australian young people aged 18–25 who were accessing online mental health help (Reachout.com, 2020), and increased downloads of mental health apps (Basu, 2020; Heilweil, 2020; Statista, 2020; Marshall et al., 2020c).

Mental health apps may seem like a good option to manage mental illness during a pandemic. After all, over 5.2 billion people worldwide own a smartphone (Barboutov et al., 2017), and these figures are growing. With such potential for wide access to mental health apps, and with ongoing difficulties accessing in-person treatment for mental health issues (Liu et al., 2020), it was little wonder that people turned to digital options during the pandemic.

Mental health apps are also attractive for general practitioners. Apps have the potential to reduce the burden on primary health care at a time when such care is dealing en masse with the acute need to treat COVID-19 (Azarang et al., 2019). It is possible that many general practitioners believed that they could “prescribe” a mental health app for their patients (Byambasuren et al., 2018) due to the shortage of in-person mental health treatment options. However, it is likely that many would not have been aware of the lack of evidence for the efficacy and effectiveness of most publicly available mental health apps.

The timing of the pandemic in relation to the present study is both serendipitous and intriguing. The baseline period for all participants commenced on January 30, 2020, and all participants were using their assigned app by February 28, 2020. In Australia, where the study was completed, the Federal Government made several key announcements (including lockdown orders and making available additional government payments for people who became unemployed) between March 12-23 (Klapdor, 2020). This 12-day period saw an increase in stress across communities, including panic buying at grocery stores (Wright, 2020).

In relation to the present study, all participants had been using their app for at least 2 weeks before the pandemic reached fever pitch in Australia. The methodology was able to detect reliable spikes in Subjective Units of Distress (SUDS) between the crucial period of March 12–23, 2020, and in the weeks and months afterward. Therefore, the study has been able to provide data on how well these five mental health apps were able to assist people to manage symptoms of anxiety and/or depression during what has arguably been the most stressful period in a generation. More broadly, the results of this study provide quality evidence of the effectiveness of these apps to help manage anxiety and/or depression during a period of massive global upheaval.

### Lessons From Pilot Work

A pilot study using a randomly chosen app from the five used in this study (*SuperBetter*) was conducted to test the feasibility of the proposed methodology. The pilot study confirmed that the methodology can be used to answer the research questions and that assertive follow-up of participants who prematurely stop providing daily SUDS ratings should be used in an effort to reduce the rate of attrition. The pilot results also provided data for comparative comments about the apparent effectiveness of the intervention when delivered in the main study and in the context of COVID-19, as the pilot study was conducted prior to COVID-19 having pandemic status.

### The Present Studies

The main objective of the research was to examine the effectiveness of five mental health apps, from a range of theoretical orientations for reducing symptoms of anxiety and/or depression. The apps selected were*SuperBetter*,*Smiling Mind*,*MoodMission*,*MindShift*, and*Destressify* (see Materials and Measures section for further details).

The protocol for this research has been published (Marshall et al., 2020b) and is registered with the Australian and New Zealand Clinical Trials Registry (ANZCTR), which is a primary registry in the World Health Organization Registry Network, registration number ACTRN12619001302145p.^4^ Readers are encouraged to refer to the published open access protocol (Marshall et al., 2020b) for further information relating to the Methods used in the present research.

The present research sought to answer the following research questions:

1. Can a range of mental health apps, employing diverse theoretical orientations, reduce subjective distress and clinically significant symptoms of anxiety and/or depression, and improve functioning in a sample of heterogeneous participants?
2. Are there specific factors about the participants that impact on the results?
3. What are the participants’ experiences of using the apps?

## Materials and Methods

The following Materials and Methods section is a summary. Refer to the published open access research protocol (Marshall et al., 2020b) for the complete Materials and Methods section.

### Participants

Inclusion criteria:

1. Eighteen years of age or older;
2. Ability to read English;
3. Have access to a smartphone or tablet device capable of connecting to the Internet and downloading the required app, and sending and receiving SMS text messages;
4. Agreeable to providing daily SUDS ratings via SMS text message and to completing self-report measures at five different time points; and
5. Mild-to-moderate anxiety and/or depression, diagnosed by a qualified health professional, and confirmed by the researchers (all of whom are clinical psychologists) after screening.

Exclusion criteria:

1. Severe anxiety and/or depression, as indicated by the initial outcome measures and in any responses to specific questions in the Demographics Questionnaire;
2. History of psychosis, or other complex mental health presentation as deemed by the researchers to be unsuitable for participation in this research (a question in the Demographics Questionnaire asked participants for their complete mental health diagnoses); and
3. Current suicidal ideation, as indicated by a participant’s responses on the initial outcome measures.

Removal criteria:

1. Not providing any SUDS rating for a 2-week period;
2. Not providing a minimum of 20 SUDS ratings in the baseline and post-intervention phases, or a minimum of 40 SUDS ratings in the intervention phase;
3. Not completing outcome measures either pre-intervention, or post-intervention;
4. Clinically significant/unsafe decline in mental health as indicated by SUDS ratings or outcome measures, or in the judgment of researchers; and
5. Suicidal ideation that has developed during the participants’ involvement in the study.

### Materials and Measures

#### The Apps

The apps used in this study were*SuperBetter* (Roepke et al., 2015; Worthen-Chaudhari et al., 2017)^5^;*Smiling Mind* (Flett et al., 2019)^6^;*MoodMission* (Bakker and Rickard, 2018; Bakker et al., 2018a,b)^7^;*MindShift* (Paul and Fleming, 2019)^8^; and*Destressify* (Lee and Jung, 2018). These apps were purposively selected on the basis of using an evidence-based treatment approach; evidence of efficacy in reducing symptoms of anxiety and/or depression and had an accompanying website with further information, including privacy statements (Note that at time of publication,*Destressify* is no longer available). In terms of theoretical orientations,*SuperBetter* uses a positive psychology framework and incorporates ideas from neuroscience in the area of neuroplasticity;*Smiling Mind* uses a structured mindfulness-based framework;*MoodMission* uses a CBT framework that emphasizes a behavioral approach, but also contains cognitive elements;*MindShift* uses a more cognitively focused CBT framework; and*Destressify* uses a less structured mindfulness-based framework compared to*Smiling Mind*. These apps were also chosen because the instructions given to participants could be equally applied across all of the apps. That is, the instruction to use the app for at least 10 min per day, for 5 days per week, would encourage participants to engage with their app and to use it for longer periods if they wished. Furthermore, 10 min per day, for 5 days per week, was deemed as dose equivalent to one “therapeutic hour” of psychological intervention each week.

#### Demographic and Biographic Features

A questionnaire was developed by the researchers to elicit demographic and biographic information.

#### Mental Health and Well-Being

The three-phase model of psychotherapy outcomes (Howard et al., 1993) was used as the framework for examining participant outcomes relating to subjective distress, symptomatology, and life functioning as follows:

1. Subjective distress: SUDS ratings – participants rated their level of distress in response to the question: “How do you feel today?,” with 0 indicating*no distress* and 10 indicating*worst possible distress*, and a score of 3 or more indicating a mild but noticeable level of upset (Wolpe and Lazarus, 1966). SUDS ratings have been shown to be a valid measure of emotional discomfort when compared with other measures of distress (*r* = 0.351,*p* < 0.05; Tanner, 2012).
2. Symptoms: The Depression Anxiety Stress Scale-21 short-form version (DASS-21; Henry and Crawford, 2005). Participants rated their experience of symptoms of depression, anxiety and stress over the previous week on a four-point scale ranging from 0 (*did not apply to me at all*) to 3 (*applied to me very much, or most of the time*). The total scores for the subscales are multiplied by two in order to interpret the severity ratings according to the longer 42-item scale (Lovibond and Lovibond, 1995; Antony et al., 1998). In this study, only the depression and anxiety subscales were used. The ratings for the depression subscale are 0–9 (*Normal*), 10–13 (*Mild*), 14–20 (*Moderate*), 21–27 (*Severe*) and 28+ (*Extremely Severe*); and, for the anxiety subscale are 0–7 (*Normal*), 8–9 (*Mild*), 10–14 (*Moderate*), 15–19 (*Severe*) and 20+ (*Extremely Severe*).
3. Life functioning: The Outcome Questionnaire-45 2nd Edition version (OQ-45.2; Boswell et al., 2013) is a 45-item self-report scale that measures distress, interpersonal relationships and social role functioning in adults 18 years and older (Beckstead et al., 2003). An index for overall life functioning is calculated (Lambert and Finch, 1999). Participants rate their feelings over the previous week on a five-point scale ranging from 0 (*never*) to 4 (*always*). Possible scores range from 0 to 180 with a total score of 63 or more being indicative of clinically significant symptoms (Lambert and Finch, 1999). Lambert et al. (2004) have suggested the following interpretive labels: >105 is*High*, 83–104 is*Moderately High*, 63–82 is*Moderate*, and <63 is*Normal*.

#### App Appraisal

The Mobile Application Rating Scale-User Version (uMARS; Stoyanov et al., 2016) is a 20-item questionnaire recording an individual’s rating on the quality of a mobile app. It contains multiple-choice and Likert-type responses and also contains a free-text field allowing users to provide a qualitative description of any aspect of the app, or their experience of using the app.

### Data Analysis

Data from this project are publicly available through the University of New England’s*Research UNE* website,^9^ DOI: 10.25952/c5nc-fq89. For further information on the data analysis plan and statistical methods used, see the published research protocol for this study (Marshall et al., 2020b).

#### Descriptive Statistics and Qualitative Accounts

Descriptive statistics were used to describe individual participant features and augment the findings from the other analyses.

#### Visual Inspection

Visual inspection was used to assess the impact of the intervention on subjective distress (SUDS). Plotted data allow for a personal judgment about the effect of an intervention (Kazdin, 2017), and in this study, visual inspection was possible using up to 122 data points of SUDS ratings (this was the highest number of individual SUDS ratings, by Participant B4 – see Supplementary Table 20 in the Supplementary Material section).

#### Time-Series Analysis

A time-series analysis was used to assess the statistical significance of changes in each participant’s plotted data across each phase of the study. Scores at the commencement of the intervention were used as the predictor in a regression model.

#### Clinical Significance and Statistical Reliability

Clinically significant symptoms of depression and anxiety, and changes in level of severity, were identified according to the published normative data for the DASS-42. Life functioning was assessed for clinical significance and change using the clinical significance index (CSI; Jacobson et al., 1999) and the reliable change index (RCI; Jacobson and Truax, 1991). Normative data for a scale are used to calculate the CSI, which is the cutoff point between the scores obtained by functional (non-clinical) and dysfunctional (clinical) populations (Jacobson and Truax, 1991; Evans et al., 1998). In this study, the CSI was used to note each participant’s clinical status pre- and post-intervention (Jacobson and Truax, 1991; Evans et al., 1998). The reliable change index (RCI) was used to assess and classify the statistical significance of any change in participants’ score from pre- to post-intervention:*Recovered* = clinically significant and statistically reliable;*Improved* = not clinically significant, but statistically reliable;*Unchanged* = not clinically significant or statistically reliable;*Deteriorated* = clinically significant and/or statistically reliable in a worsening direction.

### Procedure

The University of New England Human Research Ethics Committee approved the project on November 1, 2019, Approval Number HE19-186.

**Figure 1:**
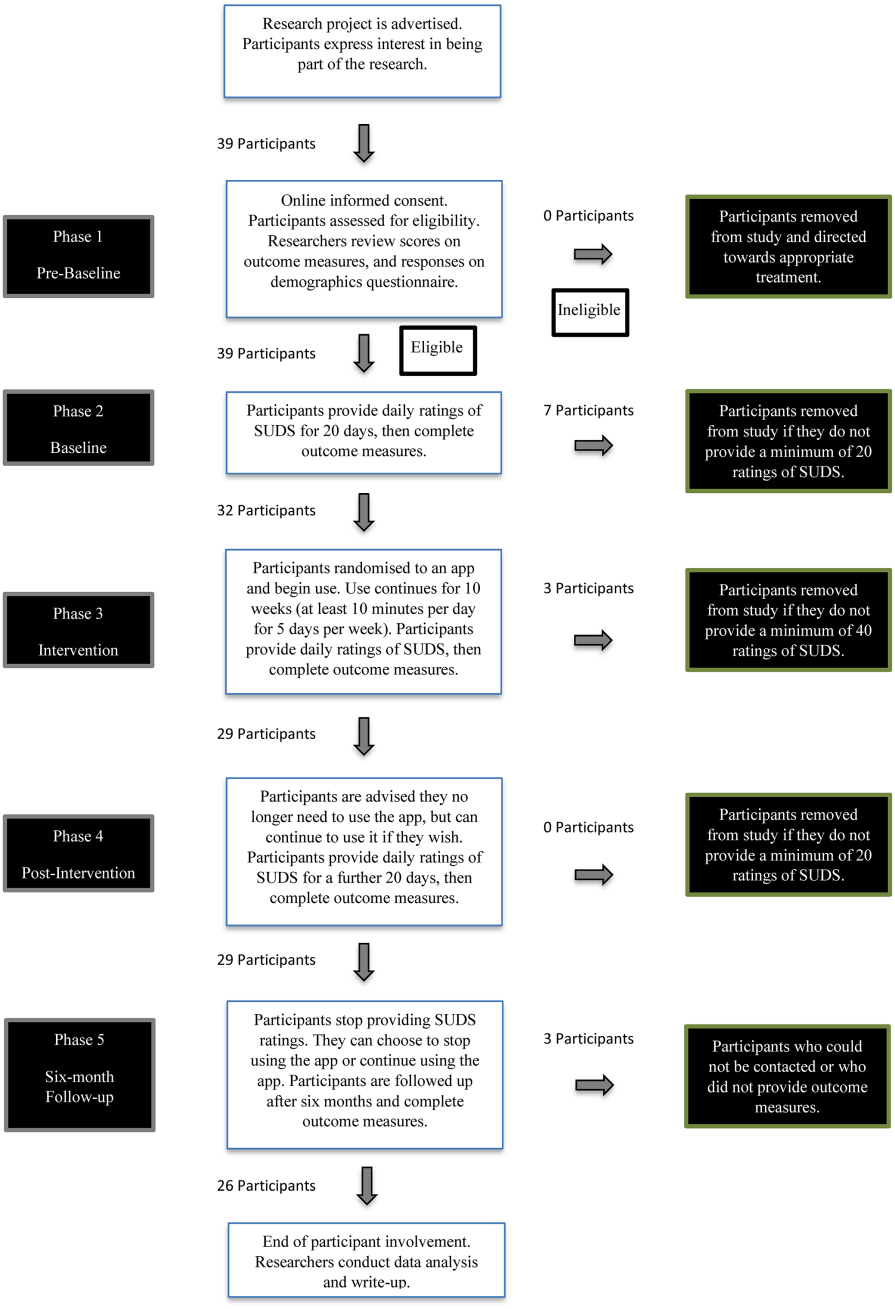
Flowchart of participant involvement and study phases.

Between November 1, 2019, and January 30, 2020, participants were recruited throughout Australia by directly approaching non-government mental health services, mental health associations (both consumer and professional), and support groups and other organizations in the mental health sector. By early December 2019, 10 participants were recruited and used in the pilot study. Another 39 participants responded to calls for expressions of interest and were used in the main study. After informed consent was obtained, participants commenced the baseline phase simultaneously and were randomly selected to begin the intervention phase in staggered order. Randomization was achieved using the online random number generator,*Research Randomizer* (Urbaniak and Plous, 2019).^10^ See Figure 1 for a flowchart of the study’s phases and participant involvement.

By using a single-case design, participants were able to be observed closely and in “real-time” allowing for highly responsive treatment. This is an important consideration in mental health research where participants may be experiencing suicidal ideation. Individual well-being was monitored by participants providing daily SUDS ratings by sending a SMS text message from their smartphone to a centrally monitored hub. While it is acknowledged that a rating out of 10 is itself limited in its ability to convey the complexities of an individual’s mental health, it can allow a mental health researcher/clinician to evaluate the relatively immediate influence of a treatment (Machalicek and Horner, 2018), adjust the intervention in response to changes in ratings, or halt the intervention and rapidly arrange crisis support if necessary (Bentley et al., 2019). However, halting the intervention or crisis support was not required for any participant.

Six participants dropped out of the pilot study prior to completing the intervention phase and were not followed up.

In the main study, 10 participants were lost to the study by the time the intervention phase had finished. A total of 29 participants completed the post-intervention phase, producing an attrition rate of 25.60%. This was substantially improved from the pilot study’s attrition rate of 60% and is attributed to assertive follow-up by the researchers when participants did not provide SUDS ratings for three consecutive days during the baseline or intervention phases. This strategy was introduced following the outcomes of the pilot study and was the single difference in methodology between the main study and the pilot study.

For a more detailed breakdown of the processes of each phase, refer to the published research protocol (Marshall et al., 2020b). The phases were identified as: phase 1 (pre-baseline), phase 2 (baseline), phase 3 (intervention), phase 4 (post-intervention), and phase 5 (6-month follow-up).

## Results: Pilot Study

Four out of 10 participants completed the pilot study with an age range of 20–49 (*M* = 35.25,*SD* = 14.9). Three participants were female; three reported comorbid anxiety and depression, and one reported anxiety disorders only; two had chronic illness of >11 years and two were receiving concurrent treatment (either psychotherapy or psychotropic medication); all were ambivalent in their motivation to comply with the app. The age range of the six participants that dropped out was 31–55 (*M* = 44.3,*SD* = 10.5), which was not significantly different to the mean age of the finishing participants [*t*(9) = −1.14,*p* = 0.29]. Four were male; two reported co-morbid anxiety and depression, and four reported depression only; five had chronic illness of >11 years and five were receiving concurrent treatment (either psychotherapy or psychotropic medication); one was strongly motivated and five were ambivalent in their motivation to comply with the app. See upplementary Tables 1–3 in the Supplementary Material section for further information regarding the demographic and biographic features of all participants in the pilot study, including those who dropped out.

Visual inspection of the plotted SUDS data for the four participants who completed the pilot study revealed that all were experiencing noticeable feelings of distress at baseline. By post-intervention, three participants had achieved a reduction in subjective distress; two to a non-noticeable level (i.e., a rating of <3). Supplementary Table 4 in the Supplementary Material section shows the mean SUDS ratings per participant by phase. Time-series analyses confirmed the findings observed through visual inspection of the plotted SUDS data. See Supplementary Figure 1; Supplementary Table 5 in the Supplementary Material section for the time-series analysis data.

The severity of each participant’s symptoms of anxiety, measured by the DASS-21 Anxiety subscale, is shown in Supplementary Table 6 in the Supplementary Material section. Two participants exhibited clinically significant improvements in anxiety from baseline to post-intervention. See Supplementary Figure 2 in the Supplementary Material section for a summary of the anxiety outcomes.

The severity of each participant’s symptoms of depression, measured by the DASS-21 Depression subscale, is shown in Supplementary Table 6. Three participants exhibited clinically significant improvements in depression from baseline to post-intervention. See Supplementary Figure 3 in the Supplementary Material section for a summary of depression outcomes.

Each participant’s overall functioning, measured by the OQ-45 Total Score, is shown in Supplementary Table 7 and illustrated in Supplementary Figure 4 in the Supplementary Material section. All participants recorded an improvement in their life functioning ratings from baseline to post-intervention. See Supplementary Table 7; Supplementary Figure 4 for a summary of life functioning outcomes.

All finishing participants showed some level of improvement in one or more areas examined by the self-report measures. The two key factors that were associated with discontinuation in the study for the six non-completers were a diagnosis of depression alone and a longer duration of mental illness. Four of the six non-completers had depression only, and five of the six non-completers had their mental illness for longer than 11 years.

See Supplementary Table 8 in the Supplementary Material section for how participants rated the app.

## Results: Main Study

### Participant Characteristics and Descriptive Statistics

A total of 39 participants commenced the main study. Of the 29 who finished the post-intervention phase, 20 were female. Seven participants (B7, C3, D3, D5, E1, E4, and E6) were assertively followed up during the study when they did not provide SUDS ratings for three consecutive days and then re-joined the study. The age-range of completers was 18–57 (*M* = 34.0,*SD* = 12.2); 16 (55.17%) had their diagnosis for 5 years or less, 15 (51.72%) were receiving concurrent counselling, and 14 (48.28%) were taking psychotropic medication. Eleven (37.93%) had an anxiety disorder only, eight (27.59%) had depression only, and 10 (34.48%) had co-morbid anxiety and depression. In terms of motivation to comply with the intervention, 10 of the completers (34.48%) agreed that their psychological health would improve, 13 (44.83%) were neutral, and six (20.69%) thought their psychological health would not improve.

Of the 10 non-completers, seven dropped out in the baseline phase and three in the intervention phase. All 10 were followed up once after not providing SUDS for 3 days and encouraged to continue in the study. The age range of the non-completers was 29–68 (*M* = 44.5,*SD* = 12.3) and was significantly different to the completers [*t*(39) = −2.34;*p* = 0.03] who were younger. For the 10 non-completers, all had depression with two (20%) having comorbid anxiety. Six (60%) agreed that the intervention would improve their psychological health, three (30%) were neutral, and one (10%) thought their psychological health would not improve.

Three participants (B2, B4, and D1) could not be contacted at 6-month follow-up.

For more information on participant characteristics, including those who dropped out of the study, see Supplementary Tables 9–18 in the Supplementary Material section.

### Effectiveness of the Apps in Reducing Subjective Distress

All the apps were able to demonstrate significant improvements in reducing subjective distress for at least two of their participants.

#### Visual Inspection

Visual inspection of the plotted SUDS data and the time-series analyses for the 29 participants who completed the study is reported below by app.

##### SuperBetter

Five participants used the*SuperBetter* app. Visual inspection revealed that four participants were experiencing noticeable feelings of distress at baseline. By post-intervention, three participants had achieved a reduction in subjective distress to a non-noticeable level (i.e., a rating of <3), but two had deteriorated. Supplementary Table 19 in the Supplementary Material section shows the mean SUDS ratings per participant by phase; Supplementary Figures 5, 6 in the Supplementary Material section display the continuous data.

##### Smiling Mind

Seven participants used the*Smiling Mind* app. Visual inspection revealed that all were experiencing noticeable feelings of distress at baseline. By post-intervention, six participants had achieved a reduction in subjective distress; five to a non-noticeable level (i.e., a rating of <3). Supplementary Table 20 in the Supplementary Material section shows the mean SUDS ratings per participant by phase; Supplementary Figures 7, 8 in the Supplementary Material section display the continuous data.

##### MoodMission

Six participants used the*MoodMission* app. Visual inspection revealed that all were experiencing noticeable feelings of distress at baseline. By post-intervention, all but one participant (C3) had achieved a reduction in subjective distress; four to a non-noticeable level (i.e., a rating of <3). Supplementary Table 21 in the Supplementary Material section shows the mean SUDS ratings per participant by phase; Supplementary Figures 9, 10 in the Supplementary Material section display the continuous data.

##### MindShift

Five participants used the*MindShift* app. Visual inspection revealed that all were experiencing noticeable feelings of distress at baseline. By post-intervention, three participants had achieved a reduction in subjective distress; two to a non-noticeable level (i.e., a rating of <3). Supplementary Table 22 in the Supplementary Material section shows the mean SUDS ratings per participant by phase; Supplementary Figures 11, 12 in the Supplementary Material section display the continuous data.

##### Destressify

Six participants used the*Destressify* app. Visual inspection revealed that all but one participant were experiencing noticeable feelings of distress at baseline. By post-intervention, four participants had achieved a reduction in subjective distress; three to a non-noticeable level (i.e., a rating of <3). Supplementary Table 23 in the Supplementary Material section shows the mean SUDS ratings per participant by phase; Supplementary Figures 13, 14 in the Supplementary Material section display the continuous data.

#### Time-Series Analysis

Time-series analyses confirmed the findings observed through visual inspection of the plotted SUDS data for each app. Using the statistical package,*R*, version 1.2.5033, an interrupted time-series analysis (ITSA) used autoregressive integrated moving average (ARIMA) models to evaluate intervention effects on each participant’s data. Autocorrelation effects were addressed using the augmented Dickey–Fuller test (Mushtaq, 2011) and Ljung–Box Q (Burns, 2002). The residuals in the models exhibited independence and normality.

The SUDS time-series analysis data are presented in Tables 1–5 and can be matched to the relevant participants in Supplementary Figures 5–14 in the Supplementary Material section.

**Table 1:** Times-series analysis results for participants A1–A5 using*SuperBetter*.

Participant	Baseline	Intervention	Post-intervention	Overall (baseline to post-intervention)
A1	*t* = 1.18,*p* = 0.26	*t* = −0.80,*p* = 0.43	*t* = −1.85,*p* = 0.08	*t* = −1.13,*p* = 0.27
A2	*t* = −1.15,*p* = 0.27	*t* = −4.87,*p* = <0.001	*t* = 2.00,*p* = 0.08	*t* = −5.51,*p* = <0.001
A3	*t* = −1.57,*p* = 0.13	*t* = 3.23,*p* = 0.00	*t* = −4.04,*p* = 0.00	*t* = 4.89,*p* = <0.001
A4	*t* = −6.78,*p* = <0.001	*t* = −0.93,*p* = 0.36	*t* = −0.54,*p* = 0.60	*t* = 3.31,*p* = <0.001
A5	*t* = −0.75,*p* = 0.46	*t* = −0.64,*p* = 0.53	*t* = −2.23,*p* = 0.04	*t* = −2.33,*p* = 0.02

**Table 2:** Time-series analysis results for participants B1–B7 using*Smiling Mind*.

Participant	Baseline	Intervention	Post-intervention	Overall (baseline to post-intervention)
B1	*t* = 3.07,*p* = 0.01	*t* = −4.74,*p* = <0.001	*t* = 0.00,*p* = 1.00	*t* = −5.52,*p* = <0.001
B2	*t* = 0.45,*p* = 0.66	*t* = −7.87,*p* = <0.001	*t* = −1.24,*p* = 0.43	*t* = −10.19,*p* = <0.001
B3	*t* = −0.80,*p* = 0.43	*t* = −1.17,*p* = 0.25	*t* = −1.24,*p* = 0.23	*t* = −4.40,*p* = <0.001
B4	*t* = −0.39,*p* = 0.70	*t* = −0.90,*p* = 0.37	*t* = −0.44,*p* = 0.66	*t* = −2.52,*p* = 0.01
B5	*t* = 1.61,*p* = 0.12	*t* = −8.59,*p* = <0.001	*t* = −0.64,*p* = 0.53	*t* = −9.11,*p* = <0.001
B6	*t* = 3.48,*p* = 0.00	*t* = −0.98,*p* = 0.33	*t* = −1.87,*p* = 0.07	*t* = −1.69,*p* = 0.10
B7	*t* = 0.47,*p* = 0.65	*t* = −5.58,*p* = <0.001	*t* = −0.33,*p* = 0.75	*t* = −8.78,*p* = <0.001

**Table 3:** Time-series analysis results for participants C1–C6 using*MoodMission*.

Participant	Baseline	Intervention	Post-intervention	Overall (baseline to post-intervention)
C1	*t* = 1.58,*p* = 0.13	*t* = −3.15,*p* = 0.00	*t* = 0.31,*p* = 0.76	*t* = −4.52,*p* = <0.001
C2	*t* = −0.69,*p* = 0.50	*t* = −2.67,*p* = 0.01	*t* = −1.67,*p* = 0.11	*t* = −5.32,*p* = <0.001
C3	*t* = −1.48,*p* = 0.15	*t* = 3.20,*p* = 0.00	*t* = −1.66,*p* = 0.11	*t* = 2.48,*p* = 0.02
C4	*t* = −0.50,*p* = 0.62	*t* = −1.82,*p* = 0.07	*t* = 0.55,*p* = 0.59	*t* = −2.99,*p* = 0.01
C5	*t* = −5.31,*p* = <0.001	*t* = −6.32,*p* = <0.001	*t* = −2.60,*p* = 0.02	*t* = −4.73,*p* = <0.001
C6	*t* = −0.31,*p* = 0.76	*t* = −2.08,*p* = 0.04	*t* = −1.72,*p* = 0.10	*t* = −0.36,*p* = 0.72

**Table 4:** Time-series analysis results for participants D1–D5 using*MindShift*.

Participant	Baseline	Intervention	Post-intervention	Overall (baseline to post-intervention)
D1	*t* = 2.67,*p* = 0.12	*t* = 1.483,*p* = 0.14	*t* = −2.28,*p* = 0.03	*t* = −1.42,*p* = 0.16
D2	*t* = −0.48,*p* = 0.64	*t* = −1.33,*p* = 0.19	*t* = −2.16,*p* = 0.04	*t* = −2.23,*p* = 0.03
D3	*t* = 0.59,*p* = 0.56	*t* = −8.02,*p* = <0.001	*t* = 1.20,*p* = 0.24	*t* = −6.73,*p* = <0.001
D4	*t* = −0.02,*p* = 0.98	*t* = −4.37,*p* = <0.001	*t* = 0.46,*p* = 0.65	*t* = −1.93,*p* = 0.06
D5	*t* = 0.04,*p* = 0.97	*t* = −3.99,*p* = <0.001	*t* = −0.14,*p* = 0.89	*t* = −0.67,*p* = 0.51

**Table 5:** Time-series analysis results for participants E1–E5 using*Destressify*.

Participant	Baseline	Intervention	Post-intervention	Overall (baseline to post-intervention)
E1	*t* = 1.98,*p* = 0.06	*t* = −1.36,*p* = 0.18	*t* = 1.01,*p* = 0.32	*t* = −3.26,*p* = 0.00
E2	*t* = −0.67,*p* = 0.51	*t* = 1.64,*p* = 0.11	*t* = −1.74,*p* = 0.09	*t* = −0.98,*p* = 0.33
E3	*t* = 1.25,*p* = 0.23	*t* = −1.21,*p* = 0.23	*t* = −4.498,*p* = <0.001	*t* = 2.67,*p* = 0.01
E4	*t* = 1.99,*p* = 0.06	*t* = −11.97,*p* = <0.001	*t* = −0.67,*p* = 0.51	*t* = −7.16,*p* = <0.001
E5	*t* = 1.25,*p* = 0.22	*t* = −0.53,*p* = 0.60	*t* = −0.65,*p* = 0.52	*t* = −5.10,*p* = <0.001
E6	*t* = 1.47,*p* = 0.16	*t* = −2.71,*p* = 0.01	*t* = 1.18,*p* = 0.25	*t* = −6.23,*p* = <0.001

### Effectiveness of the Apps for Reducing Anxiety

All the apps were able to demonstrate significant improvements in anxiety for at least three of their participants. The severity scores of each participant’s symptoms of anxiety, measured by the DASS-21 Anxiety subscale and illustrated in Supplementary Figures 15–19 in the Supplementary Material section, are reported by app below.

#### SuperBetter

Three of five participants showed improvement in their symptoms of anxiety from commencement to 6-month follow-up, but two did not. See Supplementary Figure 15 for a summary of the*SuperBetter* anxiety outcomes.

#### Smiling Mind

All seven participants showed improvement in their symptoms of anxiety from study commencement to 6-month follow-up. See Supplementary Figure 16 for a summary of the*Smiling Mind* anxiety outcomes.

#### MoodMission

Five of six participants showed improvement in their symptoms of anxiety from study commencement to 6-month follow-up, but one did not. See Supplementary Figure 17 for a summary of the*MoodMission* anxiety outcomes.

#### MindShift

Four of five participants showed improvement in their symptoms of anxiety from study commencement to 6-month follow-up, and one did not. See Supplementary Figure 18 for a summary of the*MindShift* anxiety outcomes.

#### Destressify

Five of six participants showed improvement in their symptoms of anxiety from study commencement to 6-month follow-up, but one did not. See Supplementary Figure 19 for a summary of the*Destressify* anxiety outcomes.

### Effectiveness of the Apps for Reducing Depression

All the apps were able to demonstrate significant improvements in depression for at least two of their participants. The severity scores of each participant’s symptoms of depression, measured by the DASS-21 Depression subscale and illustrated in Supplementary Figures 20–24 in the Supplementary Material section, are reported by app below.

#### SuperBetter

Two of five participants showed improvement in their symptoms of depression from study commencement to 6-month follow-up, two showed no change, and one participant had a worsening in symptoms. See Supplementary Figure 20 for a summary of*SuperBetter* depression outcomes.

#### Smiling Mind

Five of seven participants could be contacted at 6-month follow-up. Four showed improvements in symptoms of depression from study commencement to 6-month follow-up. The two participants that could not be contacted at 6-month follow-up both showed improvement in symptoms of depression from study commencement to 6-month follow-up. See Supplementary Figure 21 for a summary of*Smiling Mind* depression outcomes.

#### MoodMission

Two of six participants showed improvement in symptoms of depression from study commencement to 6-month follow-up, and four remained unchanged. See Supplementary Figure 22 for a summary of*MoodMission* depression outcomes.

#### MindShift

Four of five participants could be contacted at 6-month follow-up. One participant showed improvement in symptoms of depression from study commencement to 6-month follow-up, and three remained unchanged (one of whom was not depressed throughout). The participant who could not be contacted at 6-month follow-up had showed a worsening of symptoms of depression from study commencement to post-intervention. See Supplementary Figure 23 for a summary of*MindShift* depression outcomes.

#### Destressify

Three of six participants showed improvement in symptoms of depression from study commencement to 6-month follow-up, two remained unchanged (including one who was not depressed throughout), and one had worsened. See Supplementary Figure 24 for a summary of*Destressify* depression outcomes.

### Effectiveness of the Apps in Improving Life Functioning

**Figure 2:**
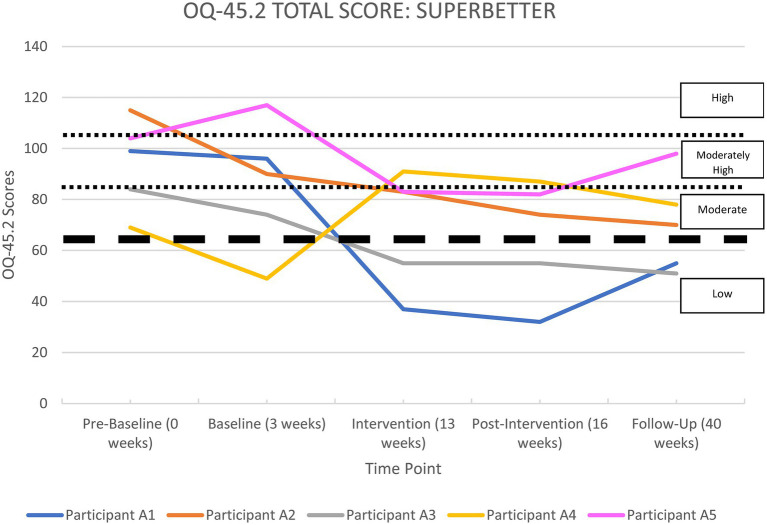
OQ-45.2 total scores for*SuperBetter*.

**Figure 3:**
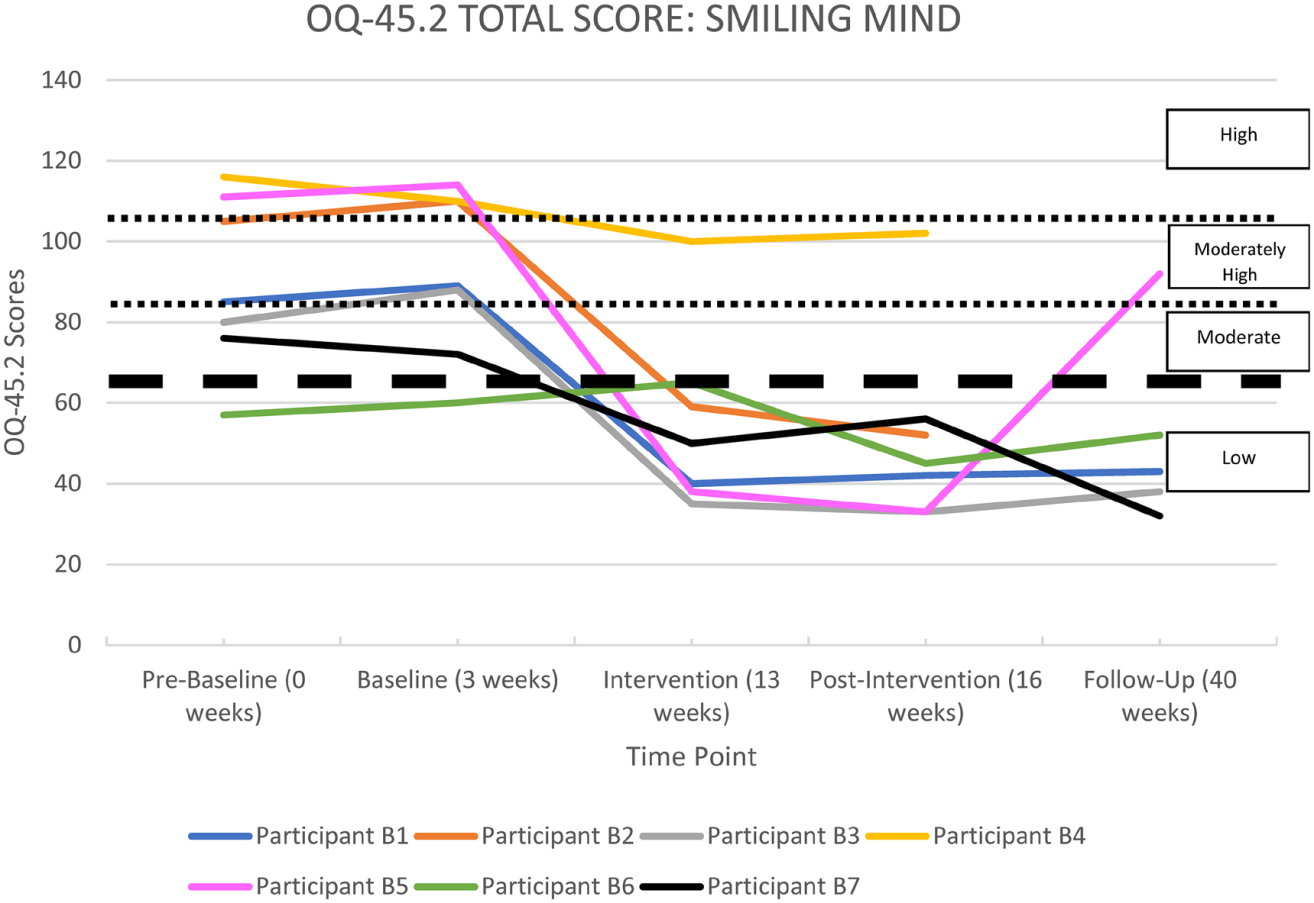
OQ-45.2 total scores for*Smiling Mind*.

**Figure 4:**
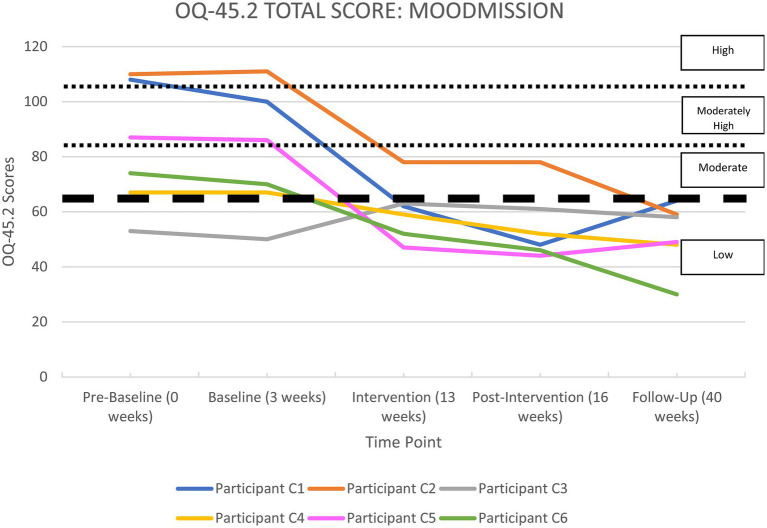
OQ-45.2 total scores for*MoodMission*.

**Figure 5:**
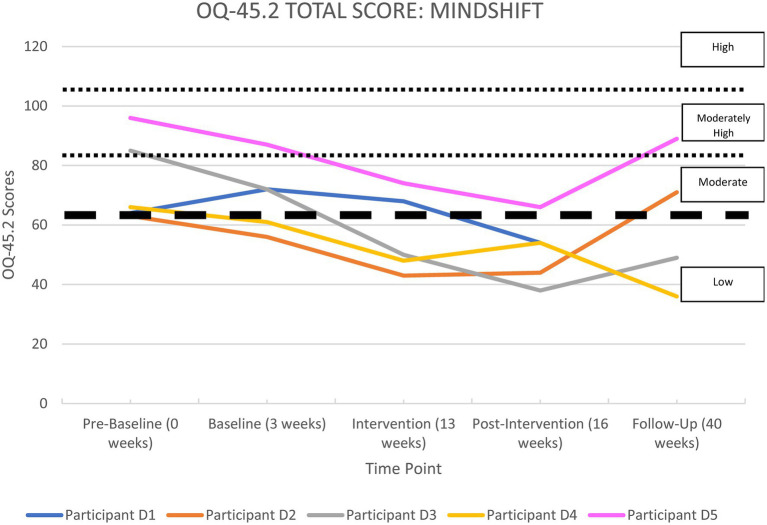
OQ-45.2 total scores for*MindShift*.

**Figure 6:**
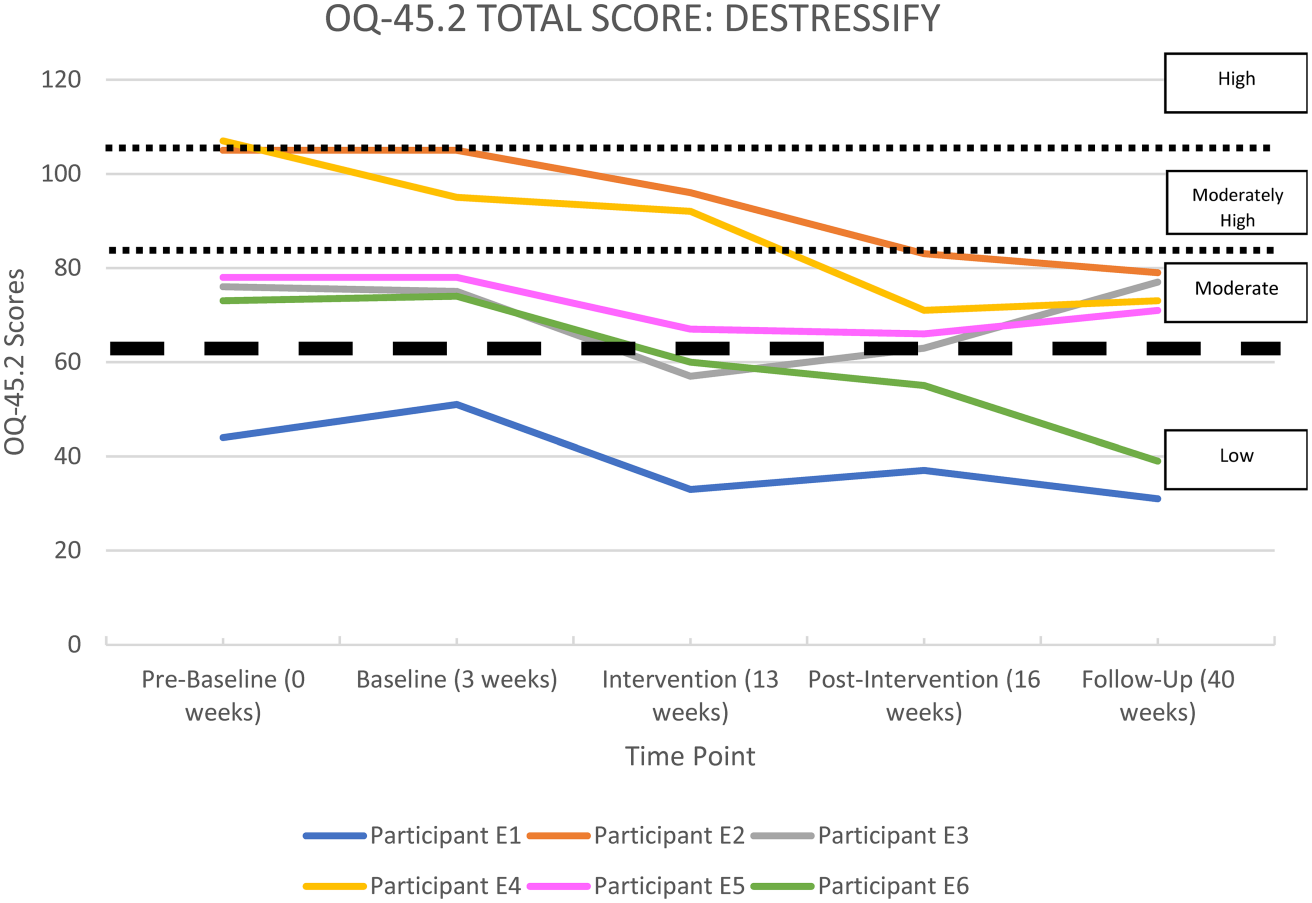
OQ-45.2 total scores for*Destressify*.

All the apps were able to demonstrate significant improvements in life functioning for at least two of their participants. Each participant’s overall functioning, measured by the OQ-45 Total Score, is shown in Supplementary Tables 24–28 in the Supplementary Material section and illustrated in Figures 2–6, and reported by app below.

#### SuperBetter

Supplementary Table 24 and Figure 2 show a summary of life functioning outcomes for participants using*SuperBetter*. As can be seen in Figure 2, at pre-baseline all five participants recorded a clinically significant impairment in life functioning. At 6-month follow-up, three participants were improved, one participant remained unchanged, and one had worsened.

#### Smiling Mind

Supplementary Table 25 and Figure 3 show a summary of life functioning outcomes for seven participants using*Smiling Mind*. As can be seen in Figure 3, at pre-baseline six participants recorded a clinically significant impairment in life functioning. At 6-month follow-up, four participants were improved and these improvements were classified as clinically significant, and one remained unchanged in the normal range. Two participants could not be contacted at 6-month follow-up, but were improved at the post-intervention phase.

#### MoodMission

Supplementary Table 26 and Figure 4 show a summary of life functioning outcomes for six participants using*MoodMission*. As can be seen in Figure 4, at pre-baseline five participants recorded a clinically significant impairment in life functioning. At 6-month follow-up, five participants were classified as showing clinically significant improvements, with one remaining under the clinically significant threshold. All participants were in the non-clinical range at 6-month follow-up.

#### MindShift

Supplementary Table 27 and Figure 5 show a summary of life functioning outcomes for five participants using*MindShift*. As can be seen in Figure 5, at pre-baseline all participants recorded a clinically significant impairment in life functioning. At 6-month follow-up, two participants recorded clinically significant improvements, two were unchanged, and one could not be contacted at 6-month follow-up, but had improved at the post-intervention phase.

#### Destressify

Supplementary Table 28 and Figure 6 show a summary of life functioning outcomes for six participants using*Destressify*. As can be seen in Figure 6, at pre-baseline five participants recorded a clinically significant impairment in life functioning. At 6-month follow-up (Phase 5), three participants had shown clinically significant improvements, and three had remained unchanged (with one participant remaining in the non-clinical range throughout the study).

### Summary of Improvements

Participants were classified into different groups based on their overall improvements in daily distress, symptomatology, and life functioning, and according to the criteria in the*Note* in Table 6. The classifications were: highly effective (those participants who responded with the greatest improvements), moderately effective (those participants who responded with moderate improvements in some areas), less effective (those participants who responded with smaller overall improvements), and did not finish. See Supplementary Table 29 in the Supplementary Material section for how participants were classified on all measures.

**Table 6:** Summary of improvements for all participants.

Improvement summary classification	Participants	Total
Highly effective	A2, B1, B2, B3, B5, B7, C5, D3	8
Moderately effective	A1, A3, A5, B4, C1, C2, C4, C6, D2, E4, E6	11
Less effective	A4, B6, C3, D1, D4, D5, E1, E2, E3, E5	10
Did not finish	A6, A7, A8, C7, C8, D6, D7, D8, E7, E8	10

Classification summary of each participant into an effectiveness group is based on author consensus of the following criteria relating to Supplementary Table 29 in the Supplementary Material section: Highly effective = 1 x “High” rating in three different categories, or “High” and “Mod” ratings across all categories; Moderately effective = 1 x “Mod” rating in three different categories, or at least 1 x “High” rating; and Less effective = 1 x “Less” rating in three different categories.

### Participant Factors That Impacted the Results

There were differences between participants who either finished the study or dropped out, and those who gained greater or lesser benefit from the intervention. Four main characteristics stood out amongst the group of participants who failed to finish the study: (a) having a diagnosis of depression alone; (b) having a longer duration of mental illness; (c) being older; and (d) having increased motivation and beliefs prior to commencing the study that their app would provide an improvement to their mental health. All participants who finished the study showed some level of improvement in one or more areas examined by the self-report measures. However, the following participant factors were associated with greater treatment effectiveness.

Treatment effectiveness tended to be greater in younger participants compared to older participants [*t*(29) = 3.24;*p* = 0.002]. The average age of the highly effective group was 26.63 (*SD* = 6.28), the moderately effective group was 34.46 (*SD* = 14.19), and the less effective group was 39.50 (*SD* = 11.25).

All participants in the highly effective group and all but two participants in the moderately effective group were receiving concurrent psychotherapy and/or psychotropic medication.

All participants in the highly effective group had their diagnosis for less than 5 years, whereas eight of the 10 participants (80%) in the less effective group had their diagnosis for greater than 6 years. Furthermore, all 10 participants who failed to finish had their diagnosis for greater than 6 years.

All participants in the highly effective group had an anxiety disorder, with six out of the eight (75%) having co-morbid depression, whereas five of the 10 participants (50%) in the less effective group had stand-alone depression, and eight of the 10 non-finishers (80%) had stand-alone depression.

All but one participant in the highly effective group (87.5%) had neutral or negative views about their motivation to do what the app suggests prior to starting the intervention (and prior to knowing which app they had been randomized to). In the less effective group, four out of 10 participants (40%) had neutral or negative views, and in the non-finishers group, 3 out of 10 participants (30%) had neutral or negative views.

### App Ratings

Overall, participants who finished the study rated their app using the uMARS as follows:*SuperBetter* 2.60 out of 5 stars (*SD* = 0.55) and 79.60 out of 130 (*SD* = 12.16);*Smiling Mind* 3.86 (*SD* = 1.46) and 100.00 (*SD* = 20.12);*MoodMission* 3.50 (*SD* = 0.55) and 98.17 (*SD* = 13.00);*MindShift* 2.60 (*SD* = 1.14) and 83.60 (*SD* = 18.20); and*Destressify* 3.00 (*SD* = 0.63) and 82.17 (*SD* = 15.83). See Supplementary Table 30 in the Supplementary Material section for further details about individual participant responses.

## Discussion

This study evaluated the effectiveness of five mental health apps:*SuperBetter*,*Smiling Mind*,*MoodMission*,*MindShift*, and*Destressify*. Participants who completed the study were diverse in terms of age, level of subjective distress, symptoms of anxiety and depression, and impairment in life functioning. Even so, all 29 participants who completed the main study recorded at least a moderate improvement in some aspect of their pre-intervention presentation, indicating that using these mobile apps may aid in improving the mental health of people with symptoms of anxiety and/or depression, including in the context of a global pandemic such as COVID-19.

At the 6-month follow-up, for most participants there was no clear pattern of changes in symptoms or functioning when compared to their ratings at post-intervention. Treatment gains were generally maintained across all measures.

### Answering the Research Questions

The present study sought to answer three research questions:

1. *Can a range of mental health apps, employing diverse theoretical orientations, reduce subjective distress and clinically significant symptoms of anxiety and/or depression, and improve functioning in a sample of heterogeneous participants?*

Given the results outlined above, it is evident that the apps are effective at improving multiple dimensions of mental health and well-being. These results enhance the previous evidence obtained from a range of methodologies, including RCT designs (Firth et al., 2017a,b). It also reflects that a range of evidence-based theoretical frameworks, when incorporated into an app, can be effective across a range of participants. In this case, the apps in this study included theoretical framework elements of CBT, mindfulness, positive psychology, and neuroplasticity.

1. *Are there specific factors about the participants that impact on the results?*

The apps have potential to offer a range of positive results over a broad cross-section of individuals. Treatment effectiveness tended to be greater in younger participants compared to older participants. This suggests that younger people get more benefit from mental health apps than older people. This may be reflective of the fact that younger people have grown up using mobile Internet-enabled devices and may therefore have greater affinity with and comfort using this technology, and who may simply rely more on their smartphone to perform a variety of tasks.

In both the main study and pilot study, those who failed to complete the study and those whose results demonstrated less improvement had a higher proportion of diagnoses of stand-alone depression. Those who completed the studies with improved results were more likely to have either stand-alone anxiety or comorbid anxiety and depression. These results suggest that these apps may be less effective for people with stand-alone depression. Detailing a reason why this might be so is difficult to postulate, given that the apps are quite different from each other from the perspective of their theoretical frameworks, functionality, and aesthetic qualities. The answer may lie in the levels of motivation of depressed participants. That is, participants who failed to finish the study were more likely to be depressed and to have high motivation. Was their motivation high because of a perception that the apps might provide a “quick fix”? When no quick fix was forthcoming, were they therefore more likely to drop out of the study? Future research on these characteristics may clarify this situation. All five apps, however, had at least one finishing participant who showed a clinically significant improvement in symptoms of depression at the conclusion of the study and in the period from post-intervention to 6-month follow-up. This suggests that the apps are still able to effectively target symptoms of depression, but may be more effective at doing so in participants who had a diagnosis of anxiety.

All participants in the highly effective group, and all but two participants in the moderately effective group, were receiving concurrent psychotherapy and/or psychotropic medication. This may potentially signal that those who are receiving concurrent psychotherapy and/or psychotropic medication may see greater benefit from using apps as an adjunct to this treatment compared to people who are using apps as a stand-alone treatment for their symptoms of anxiety and/or depression. Even though the best results were obtained by people receiving concurrent therapy and/or psychotropic medication, their stable baselines indicate that their recovery had plateaued, and improvement came after adding the app intervention. One possible explanation for this mechanism of action is that participants were instructed to use their app regularly (for at least 10 min per day, 5 days per week), which effectively means that they were doing regular “homework” to help improve their mental health. Doing such regular homework is known to aid in treatment effectiveness of therapies such as CBT (Addis and Jacobson, 2000). Additionally, adding pharmacotherapy may improve outcomes for some people also engaged in CBT (Dunlop et al., 2019). It may similarly allow some people to engage more effectively in app-delivered treatments that contain cognitive-based elements. There was no difference between participants who were taking psychotropic medication alone, those engaged in psychotherapy alone, or those who were both taking psychotropic medication and engaged in psychotherapy. That is, each of these three conditions seemed to have an equal outcome in regard to adding the use of an app as an adjunctive treatment. Additionally, most participants who were taking psychotropic medication were on an antidepressant, but other classes were mentioned as well, including benzodiazepines, mood stabilizers, and antipsychotics. Further information about the sub-type of medication was not obtained (such as whether the antidepressant was a selective serotonin reuptake inhibitor, serotonin and norepinephrine reuptake inhibitor, tricyclic etc.). See Supplementary Tables 9–18 for more information on participant demographics, including their medication status.

All 10 participants that dropped out of this study had long-term chronicity of their anxiety and/or depression diagnoses; two of these participants had had their diagnoses for 6–10 years, and the other eight had their diagnoses for over 11 years. This suggests that the use of apps to treat symptoms of anxiety and/or depression is potentially more effective for individuals with a shorter chronicity of their diagnosis. This was also suggested in similar results obtained from the pilot study.

There is further evidence of this possibility when looking at the summary classifications of participants who completed the study. Of the 10 participants classified as having less effective results overall, eight (80%) had a long-term diagnosis over 6 years. In contrast, all participants classified in the highly effective group had shorter chronicity of symptoms (less than 5 years). This, again, is evidence that perhaps apps for anxiety and/or depression are more effective for individuals who have experienced their symptoms for shorter periods.

Furthermore, there is an indication of an interaction effect. That is, the combination of younger individuals with symptoms of a shorter duration,*and* who are engaged in concurrent psychotherapy and/or psychotropic medication, may produce the most effective conditions for successful outcomes from using one of the five apps in this study.

Another participant characteristic that was present in the highly effective group of participants was a neutral or negative view about their motivation to do what the app suggests prior to starting the intervention (and prior to knowing which app they had been randomized to). Only one participant in the highly effective group had positive motivation, whereas 60 and 70%, respectively, had positive motivation in the less effective and non-finishers group. This runs counter to previous research that has found motivation to be compliant with a therapy predicts successful outcomes in treatment (Addis and Jacobson, 2000). Future research could delve deeper into this finding to determine whether mental health apps offer an effective treatment option for those individuals with low motivation toward treatment.

However, the multiple single-case design approach revealed exceptions to other demographic factors that have previously been used to predict outcomes for in-person treatment, e.g., attitudes toward mental health professionals, and the presence of another chronic medical condition, did not necessarily predict successful outcomes generally. Similarly, factors that may intuitively feel as though they should predict outcomes here, such as app star ratings, technology, and smartphone abilities, or having a particular belief prior to treatment that technology has the potential to help anxiety and/or depression, did not necessarily predict outcomes in this study. What the findings do reveal is that the smartphone apps used in this study have the potential to help a wide cross-section of people manage their symptoms of anxiety and/or depression, even in the context of a major worldwide crisis such as the COVID-19 pandemic.

One interesting participant characteristic is that level of mental health literacy (Jorm, 2012) did not necessarily predict outcomes, except to say that the mental health apps used here were effective for some individuals who had negative attitudes toward mental health professionals. For instance, three participants who used the*Smiling Mind* app reported that psychiatrists or psychologists were “harmful” for an individual’s mental health, yet all achieved excellent outcomes from using the app in areas of reducing daily levels of distress, symptoms of anxiety and/or depression, and improving life functioning. Another finding is that ability with technology and smartphones did not seem to be a factor in influencing the results, as there was a cross-section amongst participants of “average” to “excellent” abilities, according to their own self-ratings.

Comparing each of the apps, it is apparent that there are more similarities in the results than differences. Overall, it seems that the biggest impact on successful treatment effectiveness was individual participant characteristics such as being of younger age, having shorter chronicity of mental illness, receiving concurrent psychotherapy and/or taking psychotropic medication, having anxiety with or without depression (rather than stand-alone depression), and not having high motivation to engage with a mental health app.

1. *What are the participants’ experiences of using the apps?*

Although it is difficult to directly compare the results of app ratings by participants of this study to ratings from studies with a different experimental design, it would appear that the finishing participants in both the main study and pilot study rated the apps at least moderately positively. Participants using the*Smiling Mind* app rated it the highest using the combined scores from the different sections on participants’ ratings on the uMARS questionnaire (see Supplementary Table 30 in the Supplementary Material section for details), although all the apps can claim some measure of positive outcomes based on the uMARS data. However, app ratings did not necessarily correlate with treatment effectiveness, as measured by a variety of self-report methods. The 10 participants that dropped out of the study were not asked to rate their app because most dropped out before an app was assigned to them.

Emerging from this study have been insights into the benefits, facilitators, and barriers to using single-case research, conducted by practicing clinicians, to develop the evidence base for mental health apps. The findings show that a single-case research methodology is able to provide nuanced and detailed information about the effectiveness of an app. For instance, not only was it established that the apps are effective in facilitating improvements in a number of domains (subjective distress, symptoms of psychopathology, and life functioning) across a wide age range (18–57 years of age), this also occurred in a variety of contexts. These include: with individuals receiving concurrent psychotherapy and/or taking antidepressant medication; having co-morbid anxiety and depression; and having relatively low levels of motivation. Therefore, not only can a single-case research design adequately and comprehensively assess the effectiveness of a mental health app in a real-world setting, but given the automated nature of the methodology, it could easily be scaled up to accommodate more participants with the same level of detailed information available.

### COVID-19 Summary

This study provides an interesting snapshot of a digitized and automated method of psychological intervention during the worldwide COVID-19 pandemic. It has shown that five apps with an assortment of evidence-based theoretical frameworks, activities, and approaches have been effective in managing symptoms of anxiety and/or depression during the height of the COVID-19 emergency for a wide variety of participants. It provides evidence that these smartphone apps can be used for people with mild-to-moderate symptoms so that other limited in-person resources may be allocated to individuals who may have more acute and/or severe presentations, or who require sustained in-person treatment during an emergency such as COVID-19.

### Strengths of the Present Study

This study was able to closely examine the outcomes of 29 diverse participants. The design allowed detailed information on participants to be considered when examining how effective an app had been at improving their mental health and well-being. This type of nuanced information has often been unreported in previous published research on the efficacy and effectiveness of mental health apps. A further strength of the study was the relatively low attrition rate (25%) due to the assertive follow-up approach that compares favorably to the attrition rates of other studies on the efficacy and effectiveness of mental health apps (Roepke et al., 2015). Thirdly, clear instructions on how to use the apps were given to participants. Most mental health apps do not offer such precise instructions, which means they are not comparable to other evidence-based treatments, such as having weekly 50-min psychotherapy sessions, or taking a prescribed dose of psychotropic medication. Fourthly, this study was successful in providing independent evidence on the effectiveness of five apps, all of which have other published research, but lack replicated independent evidence. Finally, much of the limited previous research into the efficacy of mental health apps lacks follow-up data. This study followed up participants at 6 months, with a 90% contact rate.

### Limitations

The findings of this study should be read with the following limitations in mind. Firstly, due to the multiple single-case design, generalizations need to be made in the context of the demographics of the participants. Secondly, five participants did not exhibit statistically stable baseline periods prior to intervention commencement, which is a usual prerequisite for a multiple baseline across-individuals design (Barlow et al., 2009). This instability in subjective distress prior to the implementation of the intervention means that caution is required in interpreting the results achieved in response to the intervention for these participants, especially for the three participants whose baseline readings were trending toward improvement.

### Future Directions

This study provides a number of possible avenues of future research through the comprehensive information obtained about participant characteristics. These include age of participants, level of motivation to engage with the app, chronicity of illness, and stand-alone or comorbid diagnoses of anxiety and depression. A key area for future research should focus on the amount of concurrent intervention required to support positive outcomes with apps.

## Conclusion

This study has many potential implications for the research literature, clinical practice, and future mental health app development. In addition to providing suggestions for future research directions, this research offers a pathway for clinicians to be more engaged in the research process. This is especially so in light of the findings that suggest a mental health app has greatest effectiveness when used as an adjunctive treatment for those who are taking psychotropic medication and/or engaged in psychotherapy. Clinicians therefore have the opportunity to add the use of mental health apps to their treatment “toolbox” in their work with patients and clients. This is also relevant to app developers – it represents an opportunity to create more apps specifically targeted at clinicians for use with their patients and clients. A lack of apps with such features represents a current gap (and opportunity) in the mental health app marketplace.

This study has shown that mental health apps that have been developed by individuals and organizations with mental health expertise and contain evidence-based frameworks are able to offer support to a variety of individuals with mild-to-moderate symptoms of anxiety and depression. This offers a potential solution for those who are unable to access in-person services, such as during a time of the global unrest caused during COVID-19. The methodology of the present study demonstrates that a single-case research design is able to provide complementary evidence of efficacy alongside larger RCTs. It is possible to scale up this methodology so that it can be used for many other apps and larger numbers of participants, with the ultimate aim to give consumers greater choice of evidence-based mental health apps when searching the app stores, and ultimately more options in treating anxiety and depression.

## Data Availability Statement

The datasets presented in this study can be found in online repositories at the University of New England’s Research UNE website (https://rune.une.edu.au/web/), https://doi.org/10.25952/c5nc-fq89.

## Ethics Statement

This study was reviewed and approved by The University of New England Human Research Ethics Committee on November 1, 2019, Approval Number HE19-186. The patients/participants provided their written informed consent to participate in this study.

## Author Contributions

JM conducted the research and wrote the manuscript drafts. DD and WB supervised the research and provided editing suggestions for the manuscript drafts. All authors contributed to the article and approved the submitted version.

## Funding

JM was supported by an Australian Government Research Training Program Stipend Scholarship.

## Conflict of Interest

The authors declare that the research was conducted in the absence of any commercial or financial relationships that could be construed as a potential conflict of interest.

## Publisher’s Note

All claims expressed in this article are solely those of the authors and do not necessarily represent those of their affiliated organizations, or those of the publisher, the editors and the reviewers. Any product that may be evaluated in this article, or claim that may be made by its manufacturer, is not guaranteed or endorsed by the publisher.

## Footnotes

^1^ https://psyberguide.org/

^2^ https://headtohealth.gov.au/

^3^ https://www.nhs.uk/apps-library/

^4^ http://www.ANZCTR.org.au/ACTRN12619001302145p.aspx

^5^ https://www.superbetter.com

^6^ https://www.smilingmind.com.au/

^7^ http://moodmission.com/

^8^ https://www.anxietycanada.com/resources/mindshift-cbt/

^9^ https://rune.une.edu.au/web/

^10^ https://www.randomizer.org
