# Supplementary material for: Smartphone Psychological Therapy During COVID-19: A Study on the Effectiveness of Five Popular Mental Health Apps for Anxiety and Depression
Source: Front Psychol. 2021 Dec 13;12:775775. doi: 10.3389/fpsyg.2021.775775 (PMC8771308; doi:10.3389/fpsyg.2021.775775)
Supplement: Supplementary file 1 [file Data_Sheet_1.docx]

SUPPLEMENTARY MATERIAL FOR FRONTIERS IN PSYCHOLOGY

**Supplementary Table 1**

*Demographic and Biographic Information for Participants that Finished Pilot Study - Participants 1 to 4*

| Variable | Participant 1 | Participant 2 | Participant 3 | Participant 4 |
| --- | --- | --- | --- | --- |
| Age | 49 | 47 | 25 | 20 |
| Sex | M | F | F | F |
| Highest education | Uni | Uni | Secondary – Years 11-12 | Secondary – Years 11-12 |
| Mental health status | Depression, PTSD | Depression, panic attacks | Social anxiety, agoraphobia, generalized anxiety | Depression, social anxiety, panic attacks |
| Years with mental illness | 11+ | 11+ | 6-10 | 1-5 |
| Currently receiving counselling? | No | No | No | Yes - Psychiatrist |
| Currently on medication? | Yes - Antidepressant | No | No | Yes - Antidepressant |
| I am motivated to do what the mobile app suggests? | Somewhat disagree | Neither agree nor disagree | Neither agree nor disagree | Somewhat agree |
| Ability with technology generally | Average | Average | Good | Good |

**Supplementary Table 2**

*Demographic and Biographic Information for Participants That Failed to Finish Pilot Study – Participants 5 to 7*

| Variable | Participant 5 | Participant 6 | Participant 7 |
| --- | --- | --- | --- |
| Age | 31 | 50 | 33 |
| Sex | M | M | M |
| Highest education | Secondary – Years 11-12 | Secondary – Years 11-12 | Uni |
| Mental health status | Depression | Depression | Depression |
| Years with mental illness | 1-5 | 11+ | 11+ |
| Currently receiving counselling? | No | No | Psychologist |
| Currently on medication? | Yes - Antidepressant | No | No |
| I am motivated to do what the mobile app suggests? | Somewhat agree | Strongly agree | Neither agree nor disagree |
| Ability with technology generally | Average | Good | Average |

**Supplementary Table 3**

*Demographic and Biographic Information for Participants That Failed to Finish Pilot Study – Participants 8 to 10*

| Variable | Participant 8 | Participant 9 | Participant 10 |
| --- | --- | --- | --- |
| Age | 43 | 54 | 55 |
| Sex | F | F | M |
| Highest education | Uni | Secondary – Years 7-10 | Secondary – Years 11-12 |
| Mental health status | Depression | Depression, panic attacks, obsessive compulsive disorder | Depression, anxiety |
| Years with mental illness | 11+ | 11+ | 11+ |
| Currently receiving counselling? | No | Psychologist | Psychologist |
| Currently on medication? | Yes - Antidepressant | Yes - Antidepressant | Yes - Antidepressant |
| I am motivated to do what the mobile app suggests? | Somewhat agree | Somewhat agree | Neither agree nor disagree |
| Ability with technology generally | Excellent | Average | Good |

**Supplementary Table 4**

*SUDS Data Summary for Pilot Study*

| Participant |  | Baseline SUDS | Intervention SUDS | Post-intervention SUDS |
| --- | --- | --- | --- | --- |
| 1 | *M*  *SD*  Frequency (*N*) | 5.3  1.3  20 | 2.1  1.7  77 | 1.9  1.2  25 |
| 2 | *M*  *SD*  Frequency (*N*) | 7.0  1.5  20 | 5.8  1.4  71 | 6.1  1.6  20 |
| 3 | *M*  *SD*  Frequency (*N*) | 5.5  1.2  20 | 2.7  1.4  67 | 1.5  1.2  22 |
| 4 | *M*  *SD*  Frequency (*N*) | 5.9  1.7  21 | 3.1  1.5  68 | 3.1  1.6  22 |

**Supplementary Figure 1**

*Participant SUDS Ratings Across Each Phase of Pilot Study*


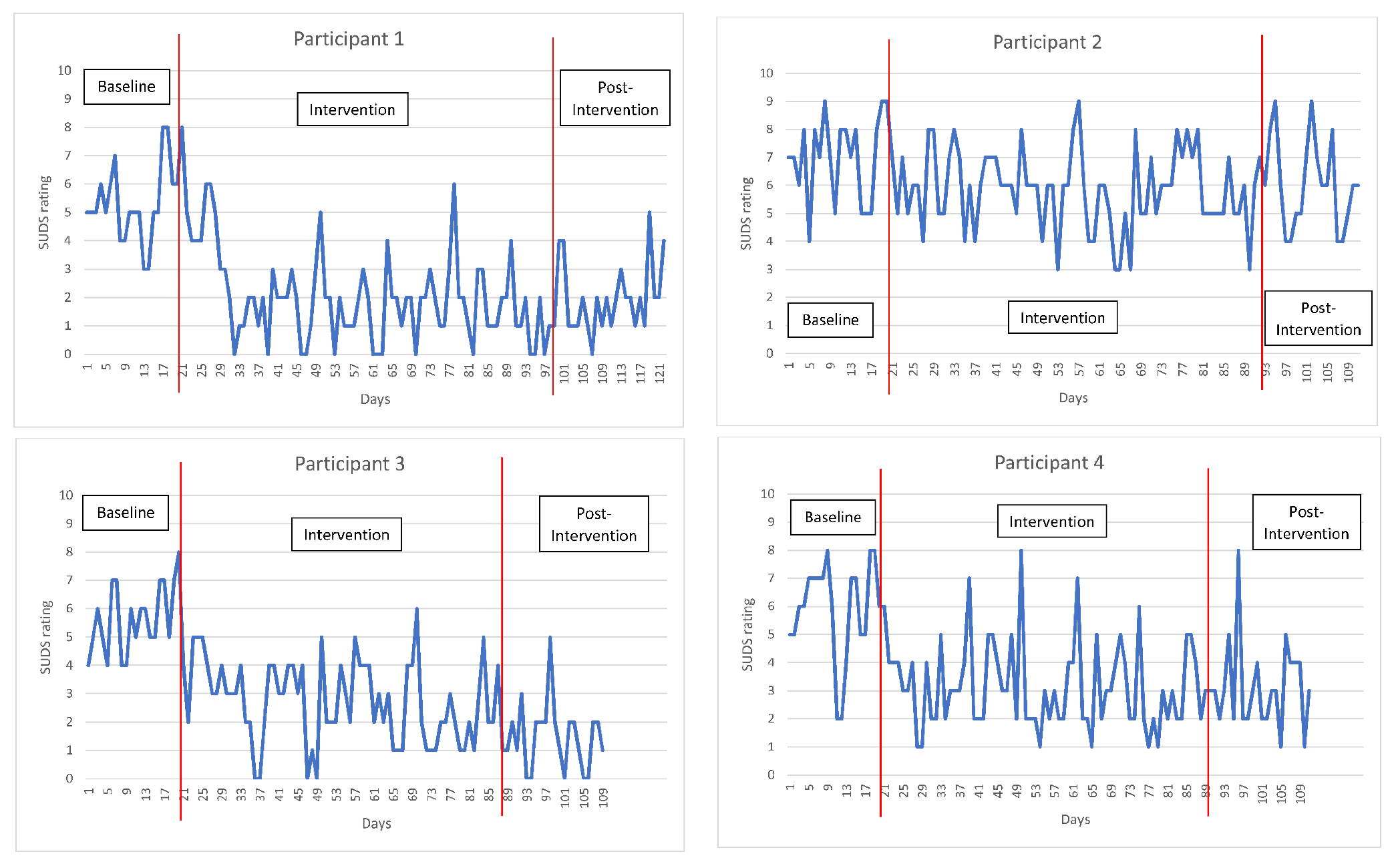


**Supplementary Table 5**

*SUDS Time Series Analysis Summary for Pilot Study*

| Participant | Baseline | Intervention | Post-intervention | Overall (baseline to post-intervention) |
| --- | --- | --- | --- | --- |
| 1 | *t* = 0.98, *p* = .34 | *t* = -3.54, *p* = .001 | *t* = 1.29, *p* = .21 | *t* = -6.62, *p* < .001 |
| 2 | *t* = 0.63, *p* = .54 | *t* = -1.07, *p* = .29 | *t* = -1.08, *p* = .29 | *t* = -0.26, *p* = .80 |
| 3 | *t* = 1.55, *p* = .14 | *t* = -2.29, *p* = .03 | *t* = -0.65, *p* = .53 | *t* = -6.72, *p* < .001 |
| 4 | *t* = -0.30, *p* = .77 | *t* = -0.80, *p* = .43 | *t* = 0.40, *p* = .97 | *t* = -3.22, *p* = .003 |

**Supplementary Table 6**

*DASS-21 Scores for Participants 1 – 4 for Pilot Study*

| Participant | Scale | Beginning to end of Baseline | Beginning to end of Intervention | Beginning to end of Post-intervention |
| --- | --- | --- | --- | --- |
| 1 | Depression | 34-26 | 26-18 | 18-16 |
|  | Anxiety | 4-4 | 4-2 | 2-6 |
| 2 | Depression | 12-10 | 10-4 | 4-4 |
|  | Anxiety | 30-20 | 20-16 | 16-14 |
| 3 | Depression | 4-6 | 6-8 | 8-10 |
|  | Anxiety | 8-12 | 12-12 | 12-14 |
| 4 | Depression | 18-16 | 16-14 | 14-12 |
|  | Anxiety | 20-18 | 18-14 | 14-10 |

*Note*. The ratings for the depression subscale are 0-9 (*Normal*), 10-13 (*Mild*), 14-20 (*Moderate*), 21-27 (*Severe*) and 28+ (*Extremely Severe*); and, for the anxiety subscale are 0-7 (*Normal*), 8-9 (*Mild*), 10-14 (*Moderate*), 15-19 (*Severe*) and 20+ (*Extremely Severe*).

**Supplementary Figure 2**


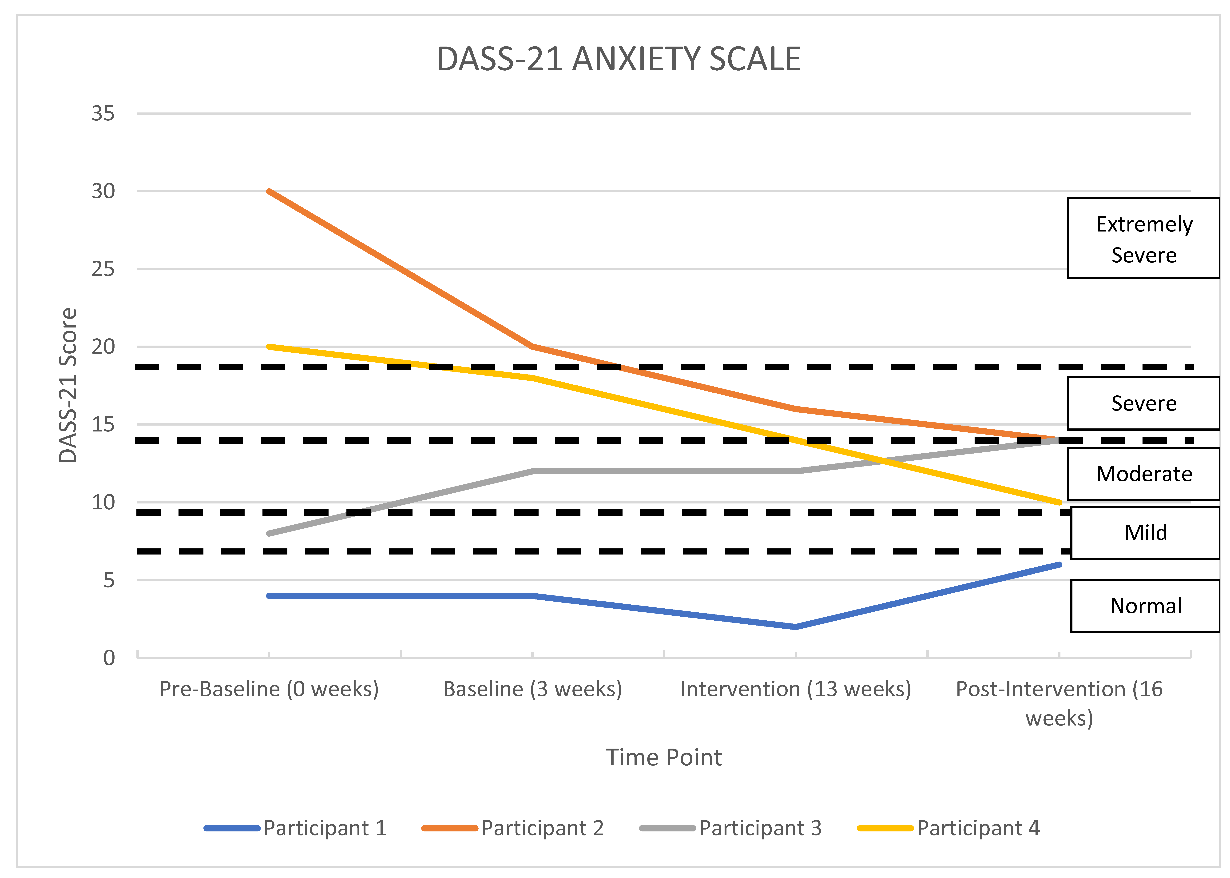
*DASS-21 Anxiety Scale Scores for Participants 1 – 4 of Pilot Study*

*Note*. Descriptive severity labels from the DASS manual (Lovibond & Lovibond, 1995).

**Supplementary Figure 3**


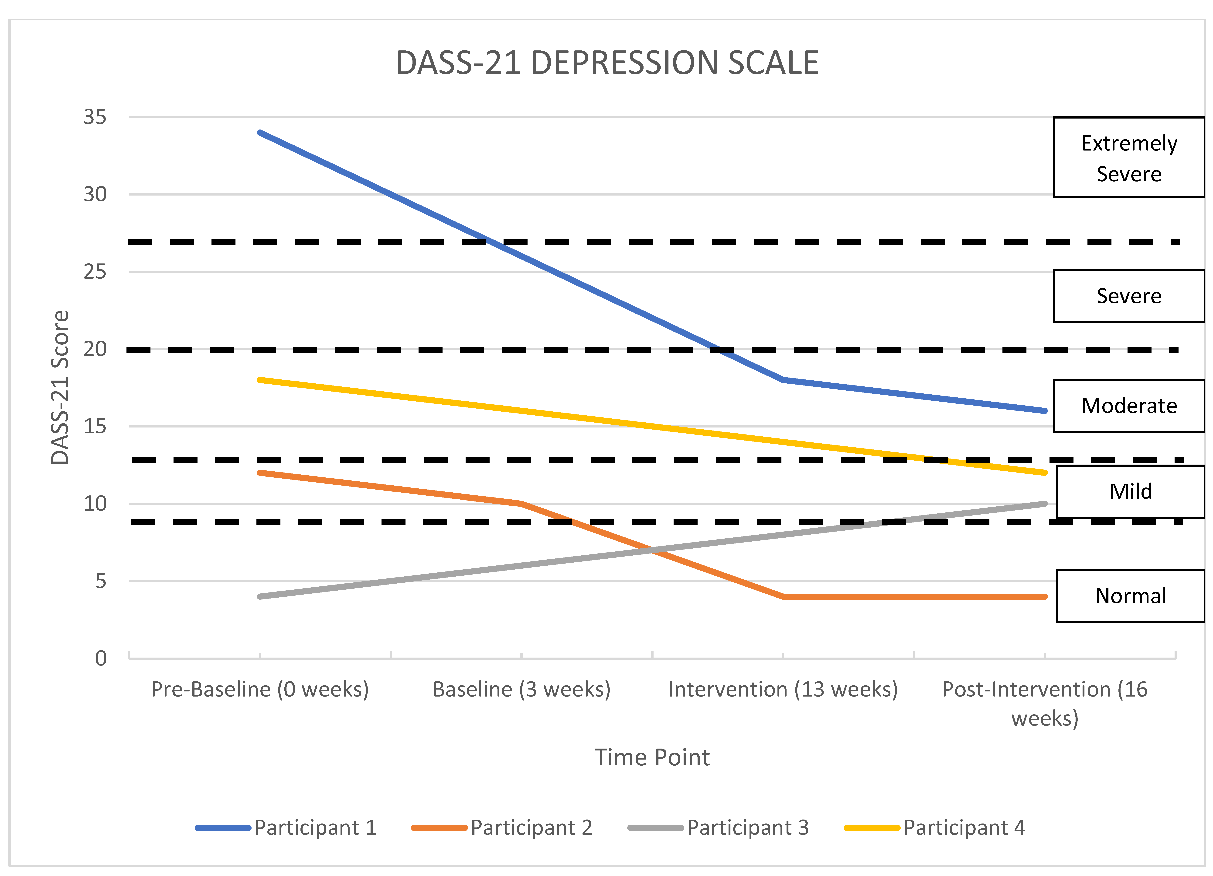
*DASS-21 Depression Scale Scores for Participants 1 – 4 of Pilot Study*

*Note*. Descriptive severity labels from the DASS manual (Lovibond & Lovibond, 1995).

**Supplementary Table 7**

*OQ-45.2 Total Scores for Participants 1 – 4 of Pilot Study*

| Participant | Pre-baseline to post-baseline | Post-baseline to post-intervention | Post-intervention to post-3-week follow up | Overall (pre-baseline to post-3-week follow up) | Classification (pre-baseline to post-3-week follow up) | Improvement (pre-baseline to post-3-week follow up) |
| --- | --- | --- | --- | --- | --- | --- |
| 1 | (91-97) ^ | 97-70 * | (70-72) ^ | 91-72 * | Improved | 20.88% |
| 2 | 122-117 ^ | 117-80 * | 80-78 ^ | 122-78 * | Improved | 36.07% |
| 3 | 74-69 ^ | 69-67 ^ | 67-47 * | 74-47 * | Improved | 36.49% |
| 4 | 104-100 ^ | (100-106) ^ | 106-94 ^ | 104-94 ^ | Unchanged | 9.62% |

*Note*. Based on RCI = 14 and CSI = 63 for Total Score. ^ denotes not statistically reliable, not clinically significant; * denotes statistically reliable (*p* < .05); + denotes clinically significant; *+ denotes statistically reliable and clinically significant. Parentheses indicate change in a worsening direction. *Recovered* = clinically significant and statistically reliable; *Improved* = not clinically significant, but statistically reliable; *Unchanged* = not clinically significant or statistically reliable; *Deteriorated* = clinically significant and/or statistically reliable in a worsening direction.

**Supplementary Figure 4**


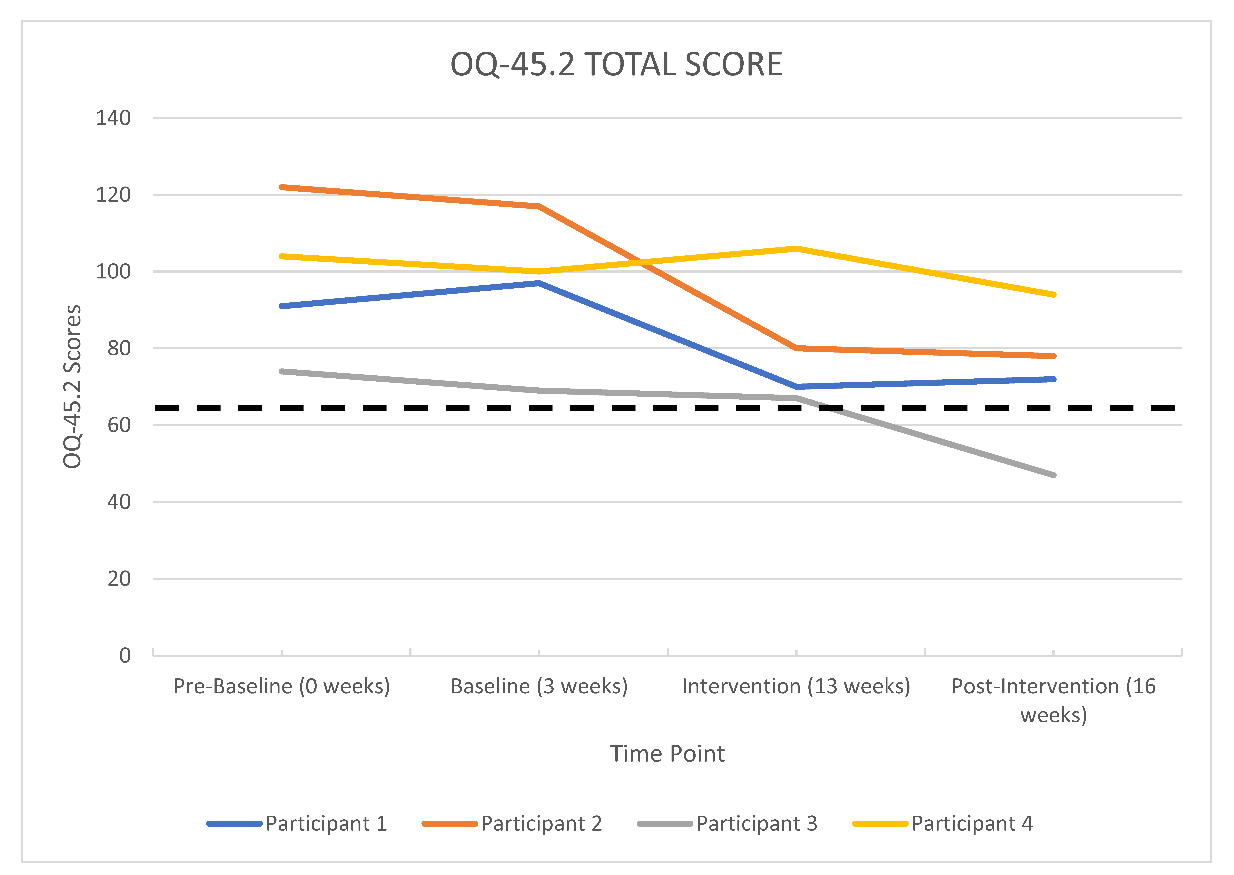
*OQ-45.2 Total Scores for Participants 1 – 4 of Pilot Study*

*Note*. Clinical cut-off of 63 from the OQ-45.2 technical manual (Lambert et al., 2004).

**Supplementary Table 8**

*uMARS App Ratings for Participants of Pilot Study*

| Participant | Section A – Engagement  (out of 25) | Section B – Functionality  (out of 20) | Section C – Aesthetics  (out of 15) | Section D – Information  (out of 20) | Section E – App subjective quality  (out of 20) | Section F – Perceived impact  (out of 30) | TOTAL  (out of 130) |
| --- | --- | --- | --- | --- | --- | --- | --- |
| 1 | 18 | 13 | 12 | 14 | 13 | 20 | 90 |
| 2 | 21 | 18 | 14 | 18 | 20 | 27 | 118 |
| 3 | 21 | 16 | 13 | 16 | 17 | 26 | 109 |
| 4 | 17 | 13 | 10 | 13 | 8 | 17 | 78 |

**Supplementary Table 9**

*Demographic and Biographic Information for Participants A1 – A4 Using* SuperBetter

| Variable | Participant A1 | Participant A2 | Participant A3 | Participant A4 |
| --- | --- | --- | --- | --- |
| Age (in years) | 26 | 36 | 35 | 19 |
| Sex | Female | Female | Female | Female |
| Highest education | University | Secondary – Years 7-10 | University | Secondary – Years 11-12 |
| Mental health status | Depression, GAD | Depression, panic attacks, PTSD, social anxiety, bipolar disorder | Panic attacks, PTSD | Depression, panic attacks, GAD |
| Years with mental illness | 1-5 | 1-5 | 1-5 | 1-5 |
| Currently receiving counselling? | Yes – Psychologist | Yes – Psychiatrist, psychologist | No | Yes - Psychologist |
| Currently on medication? | Yes – Antidepressant, antipsychotic, mood stabiliser | Yes - Benzodiazepine | No | Yes - Antidepressant |
| I am motivated to do what the mobile app suggests? | Strongly agree | Strongly agree | Strongly agree | Strongly agree |
| Ability with technology generally | Excellent | Good | Excellent | Excellent |

**Supplementary Table 10**

*Demographic and Biographic Information for Participants A5 – A8 Using* SuperBetter

| Variable | Participant A5 | Participant A6^a^ | Participant A7^a^ | Participant A8^a^ |
| --- | --- | --- | --- | --- |
| Age (in years) | 30 | 51 | 46 | 40 |
| Sex | Female | Female | Female | Male |
| Highest education | University | Secondary – Years 7-10 | University | University |
| Mental health status | Depression | Depression | Depression | Depression |
| Years with mental illness | 1-5 | > 11 | > 11 | > 11 |
| Currently receiving counselling? | No | Yes – Psychologist | No | No |
| Currently on medication? | Yes – Antidepressant | No | No | Yes - Antidepressant |
| I am motivated to do what the mobile app suggests? | Somewhat agree | Strongly agree | Neutral | Somewhat agree |
| Ability with technology generally | Average | Good | Average | Excellent |

^a^ Participants that dropped out of the study.

**Supplementary Table 11**

*Demographic and Biographic Information for Participants B1 – B4 Using* Smiling Mind

| Variable | Participant B1 | Participant B2 | Participant B3 | Participant B4 |
| --- | --- | --- | --- | --- |
| Age (in years) | 20 | 23 | 24 | 55 |
| Sex | Female | Male | Female | Female |
| Highest education | Secondary – Years 11-12 | University | Secondary – Years 11-12 | University |
| Mental health status | Depression, panic attacks, GAD | Depression, social anxiety, autism | Panic attacks, OCD, social anxiety | Depression |
| Years with mental illness | 1-5 | 1-5 | 1-5 | > 11 |
| Currently receiving counselling? | Yes – Psychologist | Yes – Psychiatrist, psychologist | Yes - Counsellor | No |
| Currently on medication? | No | Yes – Antidepressant | No | Yes - Antidepressant |
| I am motivated to do what the mobile app suggests? | Somewhat disagree | Neutral | Neutral | Somewhat agree |
| Ability with technology generally | Good | Excellent | Excellent | Poor |

**Supplementary Table 12**

*Demographic and Biographic Information for Participants B5 – B7 Using* Smiling Mind

| Variable | Participant B5 | Participant B6 | Participant B7 |
| --- | --- | --- | --- |
| Age (in years) | 18 | 42 | 30 |
| Sex | Female | Female | Male |
| Highest education | Secondary – Years 11-12 | Secondary – Years 7-10 | University |
| Mental health status | Panic attacks, social anxiety, GAD | Depression | Depression, GAD |
| Years with mental illness | 1-5 | 6-10 | 1-5 |
| Currently receiving counselling? | Yes - Psychologist | No | Psychologist |
| Currently on medication? | No | No | Yes - ? |
| I am motivated to do what the mobile app suggests? | Neutral | Somewhat agree | Somewhat disagree |
| Ability with technology generally | Good | Average | Good |

**Supplementary Table 13**

*Demographic and Biographic Information for Participants C1 – C4 Using* MoodMission

| Variable | Participant C1 | Participant C2 | Participant C3 | Participant C4 |
| --- | --- | --- | --- | --- |
| Age (in years) | 27 | 47 | 42 | 20 |
| Sex | Female | Male | Female | Female |
| Highest education | Secondary – Years 11-12 | Secondary – Years 11-12 | University | Secondary – Years 11-12 |
| Mental health status | Depression, panic attacks, OCD | Depression | Panic attacks, GAD | Social anxiety |
| Years with mental illness | 6-10 | > 11 | > 11 | 1-5 |
| Currently receiving counselling? | Yes – Psychologist | No | No | Yes - Counsellor |
| Currently on medication? | No | Yes - Antidepressant | No | No |
| I am motivated to do what the mobile app suggests? | Neutral | Neutral | Neutral | Somewhat agree |
| Ability with technology generally | Excellent | Good | Poor | Average |

**Supplementary Table 14**

*Demographic and Biographic Information for Participants C5 – C8 Using* MoodMission

| Variable | Participant C5 | Participant C6 | Participant C7^a^ | Participant C8^a^ |
| --- | --- | --- | --- | --- |
| Age (in years) | 31 | 44 | 30 | 35 |
| Sex | Female | Female | Male | Male |
| Highest education | University | University | Secondary – Years 11-12 | Secondary – Years 11-12 |
| Mental health status | Depression, GAD | Panic attacks, PTSD, GAD | Depression | Depression, PTSD |
| Years with mental illness | 1-5 | > 11 | 6-10 | > 11 |
| Currently receiving counselling? | Yes - Psychologist | No | Yes – Psychologist | No |
| Currently on medication? | No | No | No | Yes - Antidepressant |
| I am motivated to do what the mobile app suggests? | Somewhat disagree | Neutral | Strongly agree | Neutral |
| Ability with technology generally | Average | Poor | Excellent | Good |

^a^ Participants that dropped out of the study.

**Supplementary Table 15**

*Demographic and Biographic Information for Participants D1 – D4 Using* MindShift

| Variable | Participant D1 | Participant D2 | Participant D3 | Participant D4 |
| --- | --- | --- | --- | --- |
| Age (in years) | 49 | 57 | 31 | 22 |
| Sex | Male | Female | Female | Female |
| Highest education | Secondary – Years 11-12 | University | Secondary – Years 11-12 | Secondary – Years 11-12 |
| Mental health status | Depression | GAD | Depression, panic attacks, Borderline personality disorder | GAD |
| Years with mental illness | > 11 | > 11 | 1-5 | 1-5 |
| Currently receiving counselling? | No | No | Yes - Psychologist | Yes - Psychologist |
| Currently on medication? | Yes - Antidepressant | Yes - Antidepressant | No | No |
| I am motivated to do what the mobile app suggests? | Somewhat agree | Neutral | Somewhat disagree | Somewhat agree |
| Ability with technology generally | Average | Poor | Excellent | Good |

**Supplementary Table 16**

*Demographic and Biographic Information for Participants D5 – D8 Using* MindShift

| Variable | Participant D5 | Participant D6^a^ | Participant D7^a^ | Participant D8^a^ |
| --- | --- | --- | --- | --- |
| Age (in years) | 52 | 52 | 39 | 55 |
| Sex | Male | Female | Female | Female |
| Highest education | Secondary – Years 7-10 | University | University | University |
| Mental health status | Depression | Depression | Depression | Depression |
| Years with mental illness | > 11 | > 11 | 6-10 | > 11 |
| Currently receiving counselling? | No | Yes - Counsellor | No | Yes - Psychologist |
| Currently on medication? | Yes - Antidepressant | No | Yes - ? | Yes - Antidepressant |
| I am motivated to do what the mobile app suggests? | Somewhat disagree | Strongly agree | Somewhat agree | Somewhat agree |
| Ability with technology generally | Good | Good | Good | Excellent |

^a^ Participants that dropped out of the study.

**Supplementary Table 17**

*Demographic and Biographic Information for Participants E1 – E4 Using* Destressify

| Variable | Participant E1 | Participant E2 | Participant E3 | Participant E4 |
| --- | --- | --- | --- | --- |
| Age (in years) | 46 | 39 | 35 | 18 |
| Sex | Male | Male | Male | Female |
| Highest education | University | University | University | Secondary – Years 11-12 |
| Mental health status | Panic attacks, social anxiety, autism | Depression, bipolar disorder | Depression, PTSD | Panic attacks, GAD |
| Years with mental illness | > 11 | > 11 | > 11 | 1-5 |
| Currently receiving counselling? | No | No | No | Yes - Psychologist |
| Currently on medication? | No | Yes – Antidepressant, antipsychotic | Yes – Antidepressant, benzodiazepine | No |
| I am motivated to do what the mobile app suggests? | Neutral | Somewhat disagree | Strongly agree | Strongly agree |
| Ability with technology generally | Excellent | Average | Average | Average |

**Supplementary Table 18**

*Demographic and Biographic Information for Participants E5 – E8 Using* Destressify

| Variable | Participant E5 | Participant E6 | Participant E7^a^ | Participant E8^a^ |
| --- | --- | --- | --- | --- |
| Age (in years) | 49 | 20 | 29 | 68 |
| Sex | Female | Male | Female | Female |
| Highest education | University | Secondary – Years 11-12 | University | Secondary – Years 7-10 |
| Mental health status | Depression | Depression, panic attacks, GAD | Depression, PTSD | Depression |
| Years with mental illness | > 11 | 1-5 | > 11 | > 11 |
| Currently receiving counselling? | No | Yes - Psychologist | Yes – Psychologist | No |
| Currently on medication? | Yes - Antidepressant | No | Yes - Antidepressant | Yes - Antidepressant |
| I am motivated to do what the mobile app suggests? | Strongly agree | Somewhat agree | Strongly agree | Neutral |
| Ability with technology generally | Average | Excellent | Excellent | Good |

^a^ Participants that dropped out of the study.

Supplementary Table 19

*SUDS Data Summary for Participants Using* SuperBetter

| Participant |  | Baseline SUDS | Intervention SUDS | Post-intervention SUDS |
| --- | --- | --- | --- | --- |
| A1 | *M*  *SD*  Frequency (*n*) | 3.7  1.8  21 | 2.3  2.5  51 | 1.7  1.6  22 |
| A2 | *M*  *SD*  Frequency (*n*) | 4.3  1.7  22 | 2.7  2.1  64 | 1.2  0.8  21 |
| A3 | *M*  *SD*  Frequency (*n*) | 2.6  2.0  26 | 5.8  2.4  70 | 4.5  1.7  25 |
| A4 | *M*  *SD*  Frequency (*n*) | 4.3  1.5  21 | 6.0  1.5  64 | 6.1  1.3  28 |
| A5 | *M*  *SD*  Frequency (*n*) | 5.0  1.2  23 | 3.5  1.6  67 | 2.9  1.2  26 |

**Supplementary Figure 5**


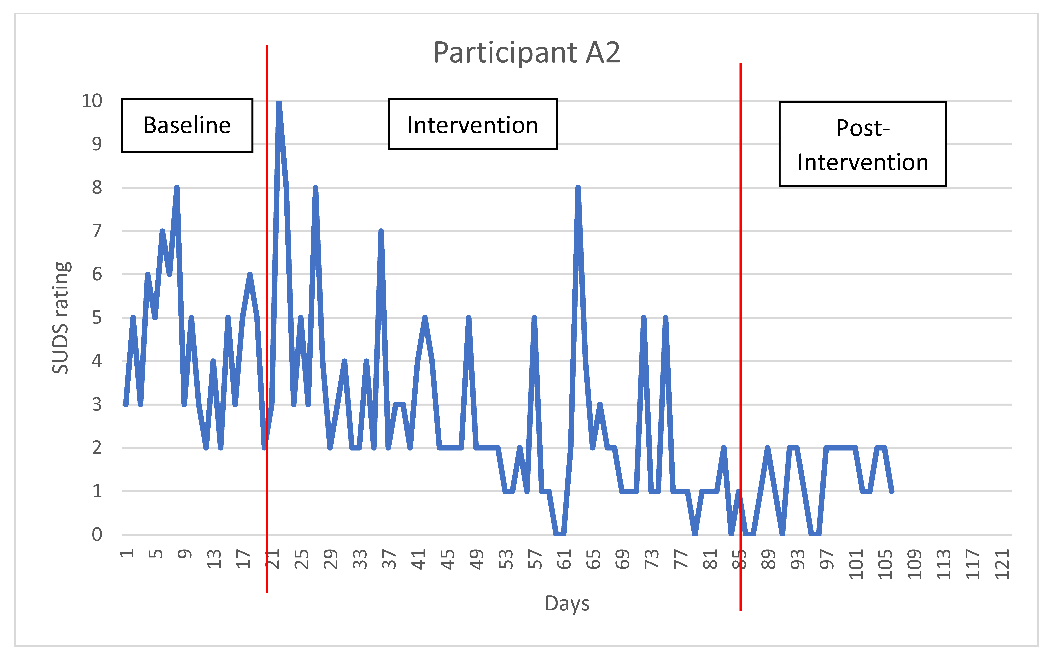

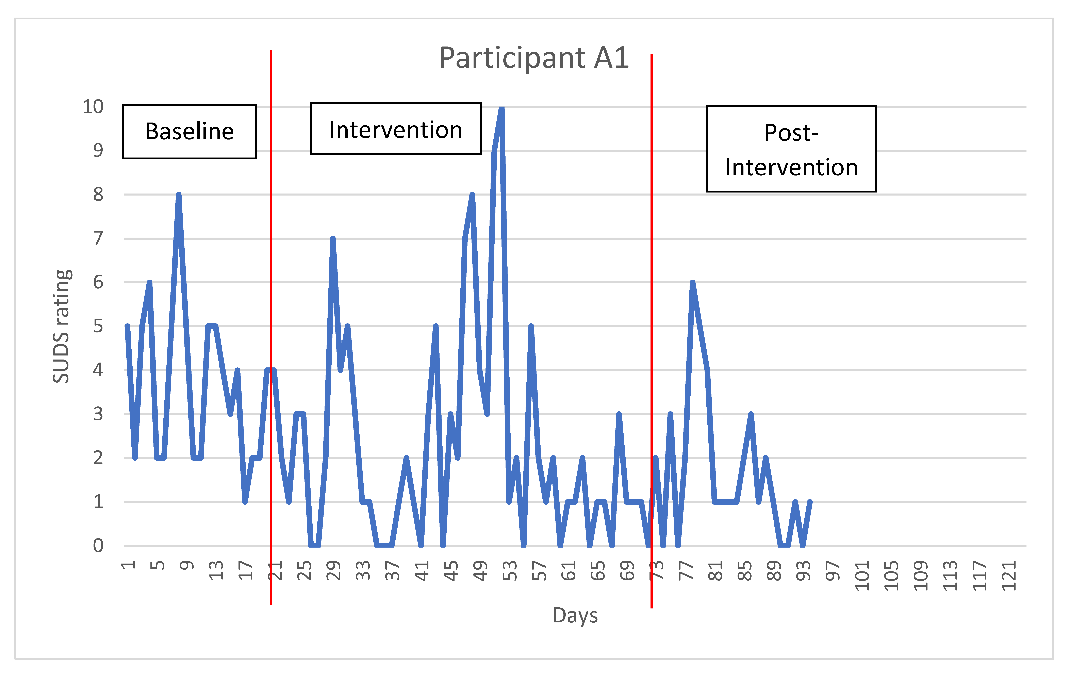
*SUDS Ratings for Participants A1 – A4 Using* SuperBetter


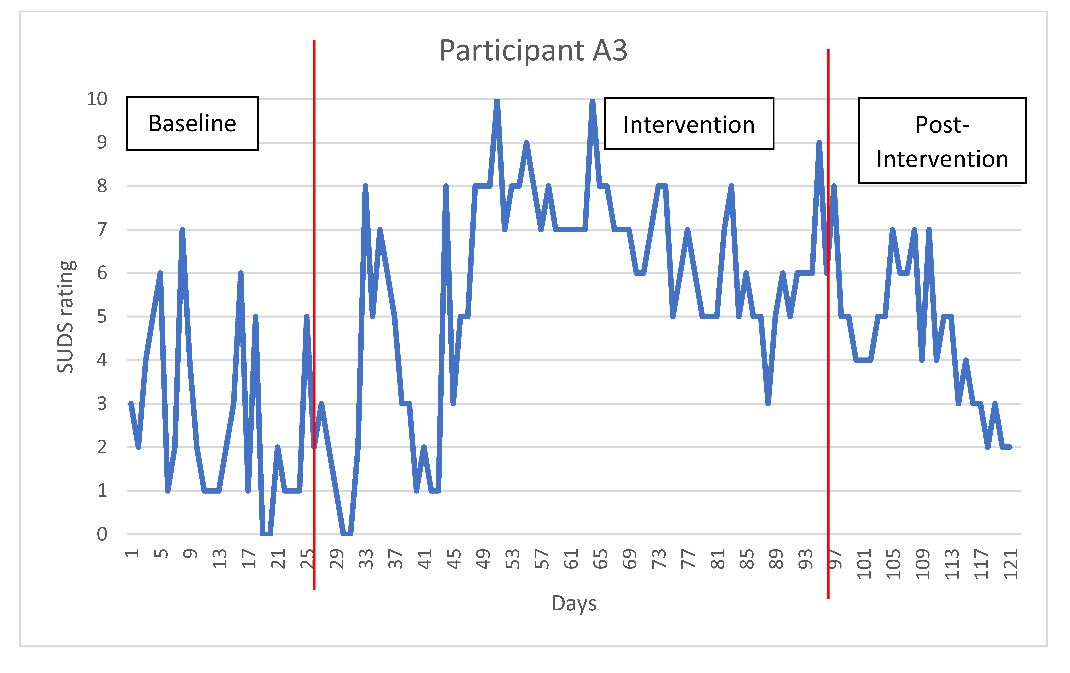

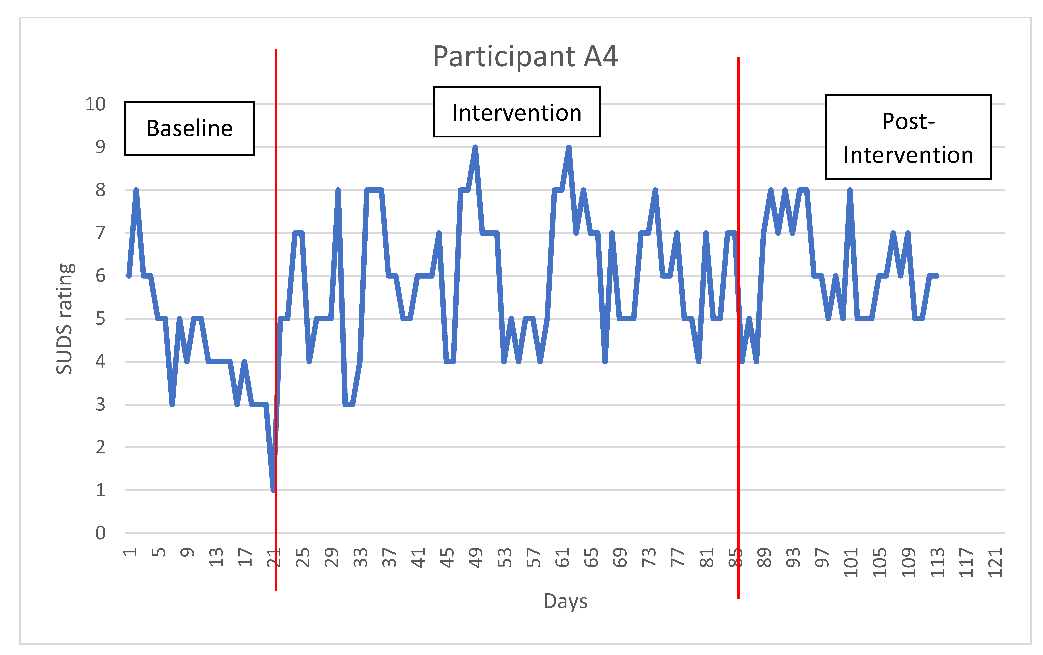


**Supplementary Figure 6**

*SUDS Ratings for Participant A5 Using* SuperBetter

*
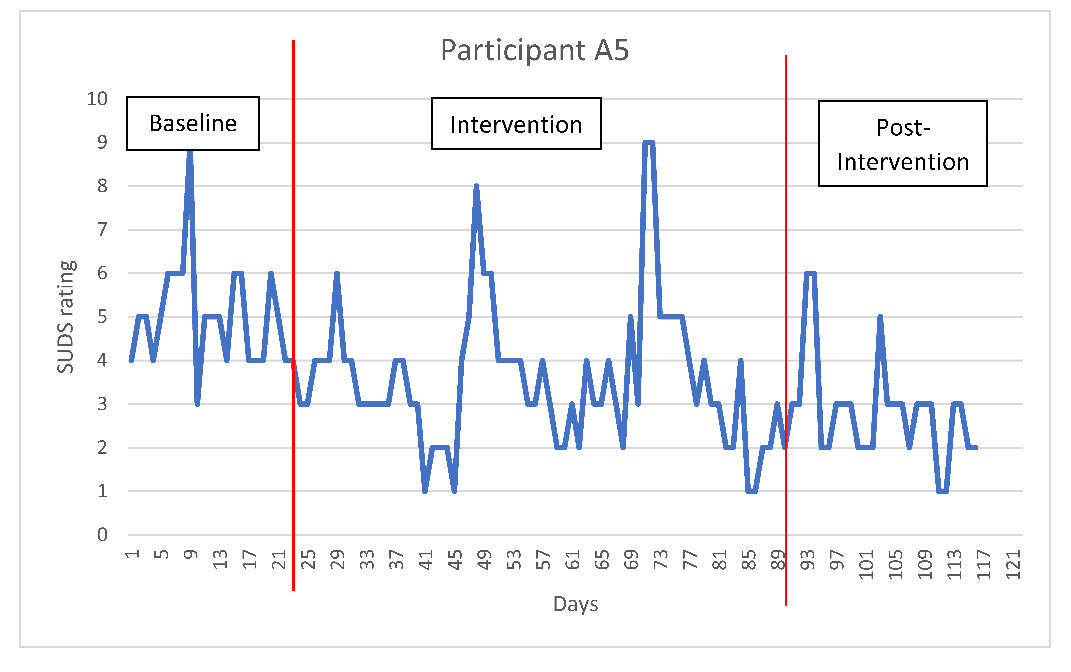
*

Supplementary Table 20

*SUDS Data Summary for Participants Using* Smiling Mind

| Participant |  | Baseline SUDS | Intervention SUDS | Post-intervention SUDS |
| --- | --- | --- | --- | --- |
| B1 | *M*  *SD*  Frequency (*n*) | 5.0  1.6  22 | 3.5  2.0  65 | 1.6  0.9  25 |
| B2 | *M*  *SD*  Frequency (*n*) | 7.1  1.0  21 | 4.7  1.7  65 | 3.0  0.6  25 |
| B3 | *M*  *SD*  Frequency (*n*) | 5.1  1.1  23 | 4.0  1.8  66 | 2.8  0.8  26 |
| B4 | *M*  *SD*  Frequency (*n*) | 6.8  1.2  27 | 5.9  2.0  68 | 4.9  1.5  27 |
| B5 | *M*  *SD*  Frequency (*n*) | 7.2  1.2  23 | 3.0  2.0  72 | 2.0  1.2  26 |
| B6 | *M*  *SD*  Frequency (*n*) | 3.7  1.2  23 | 2.6  1.0  66 | 2.2  1.0  25 |
| B7 | *M*  *SD*  Frequency (*n*) | 5.5  0.7  22 | 4.0  2.1  70 | 2.1  0.9  26 |

**Supplementary Figure 7**

*SUDS Ratings for Participants B1 – B4 Using* Smiling Mind


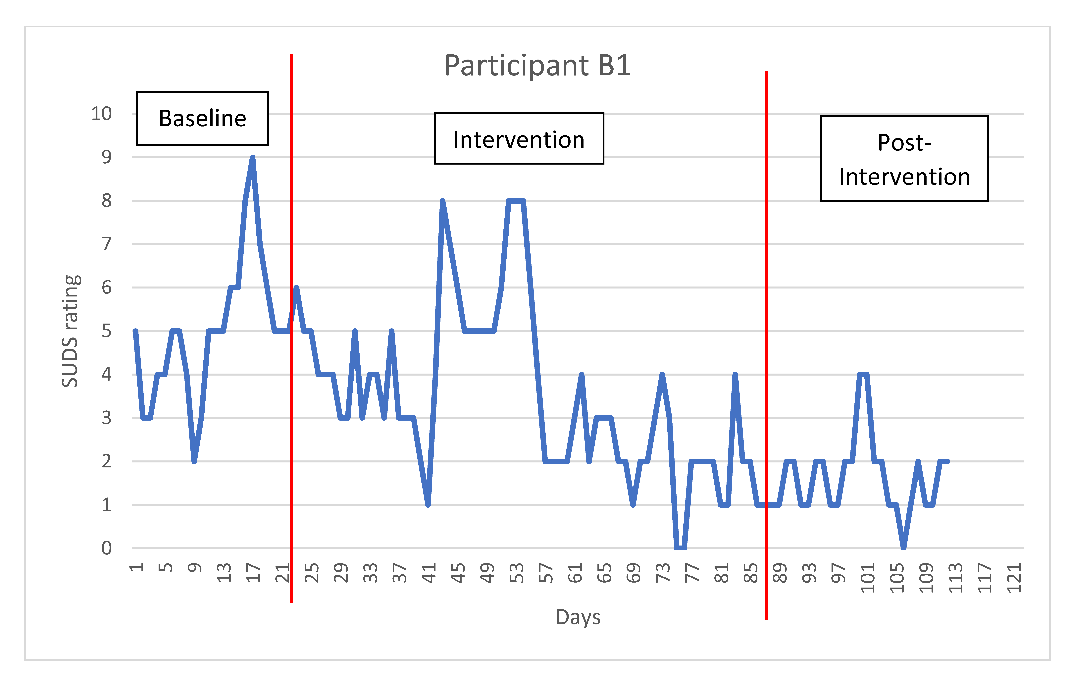

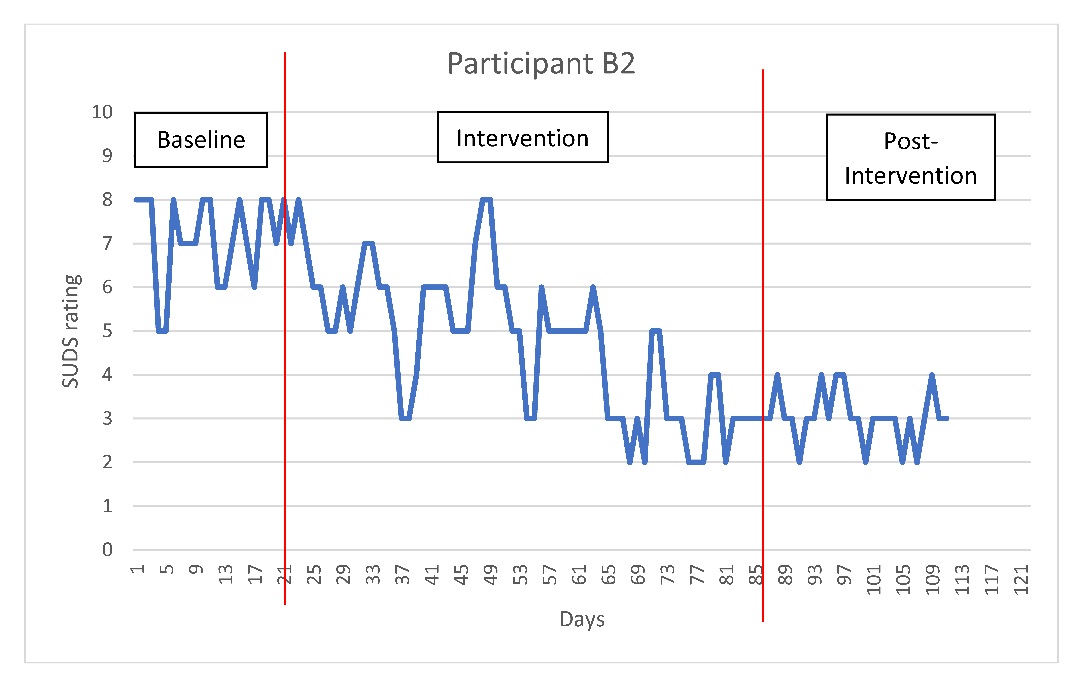

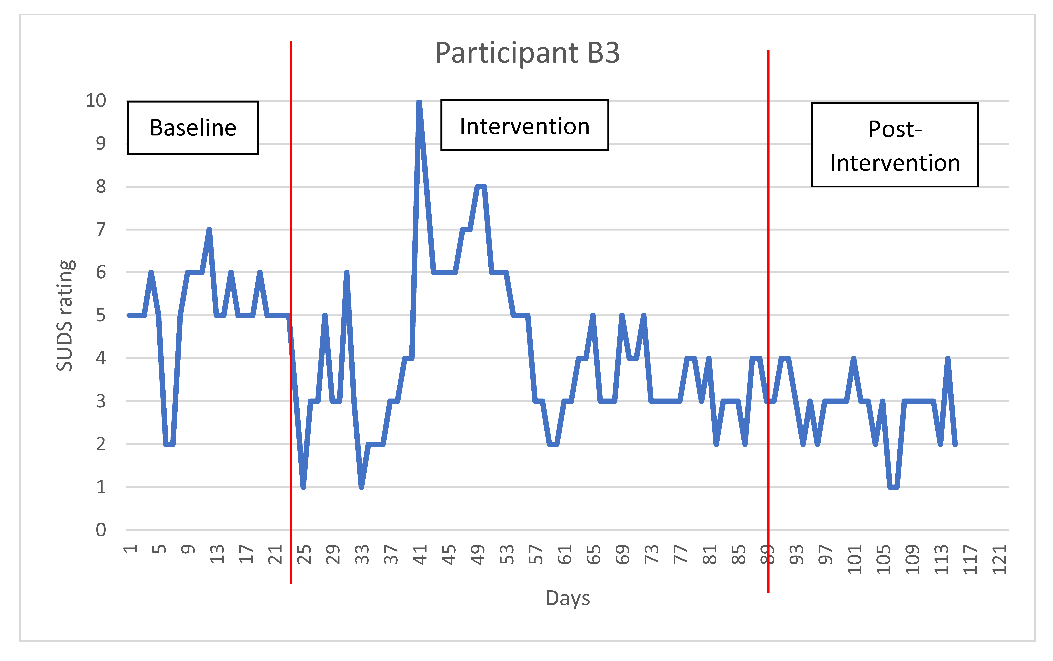

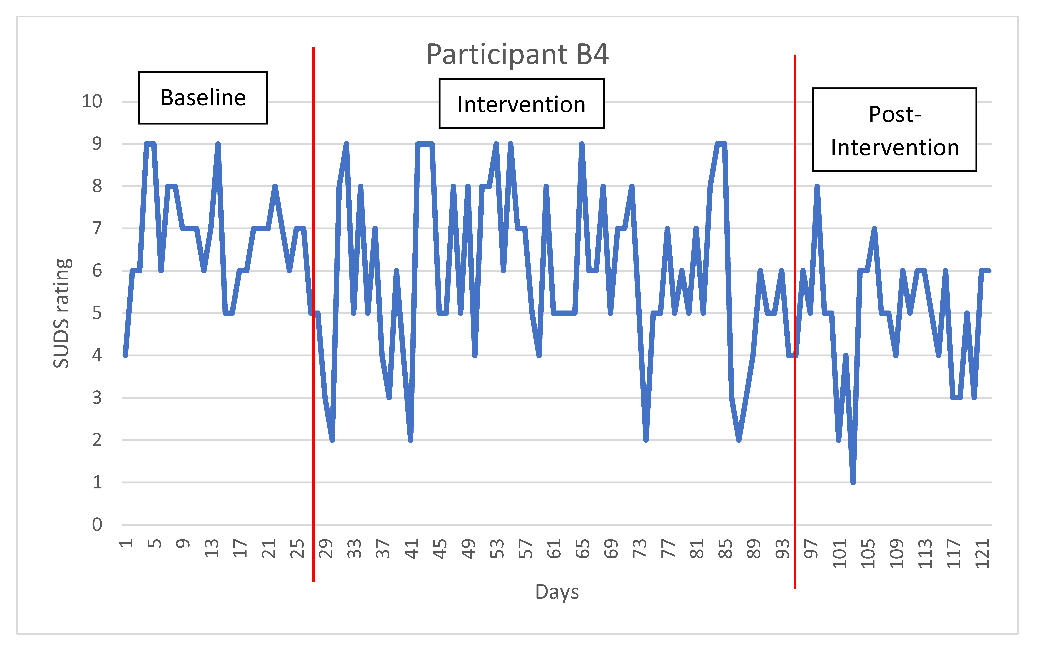


**Supplementary Figure 8**


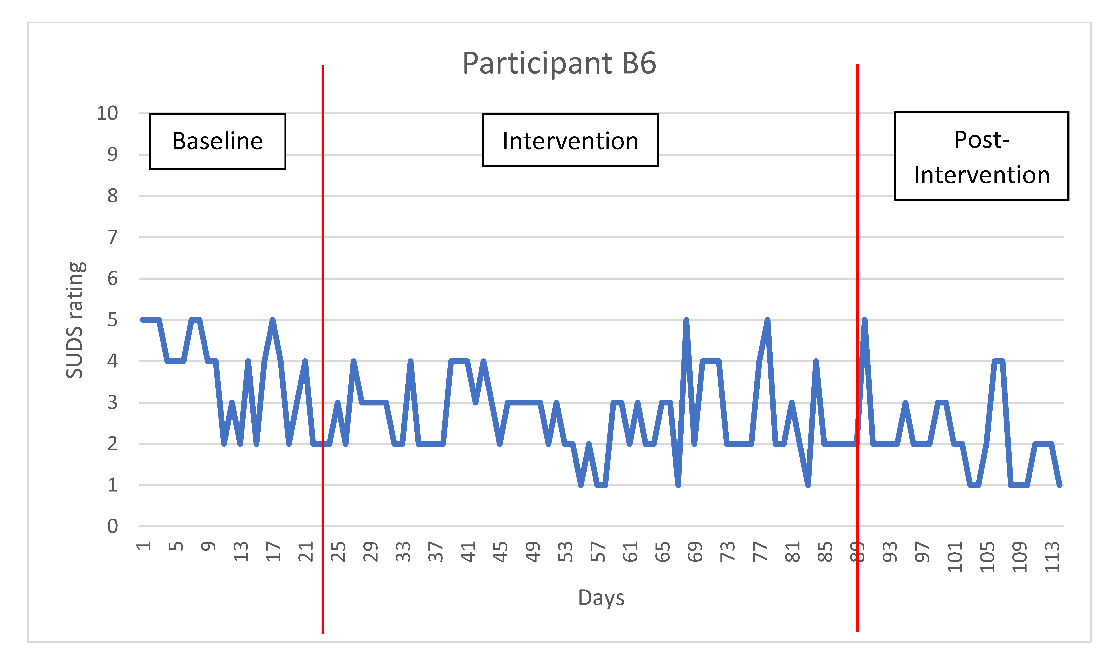

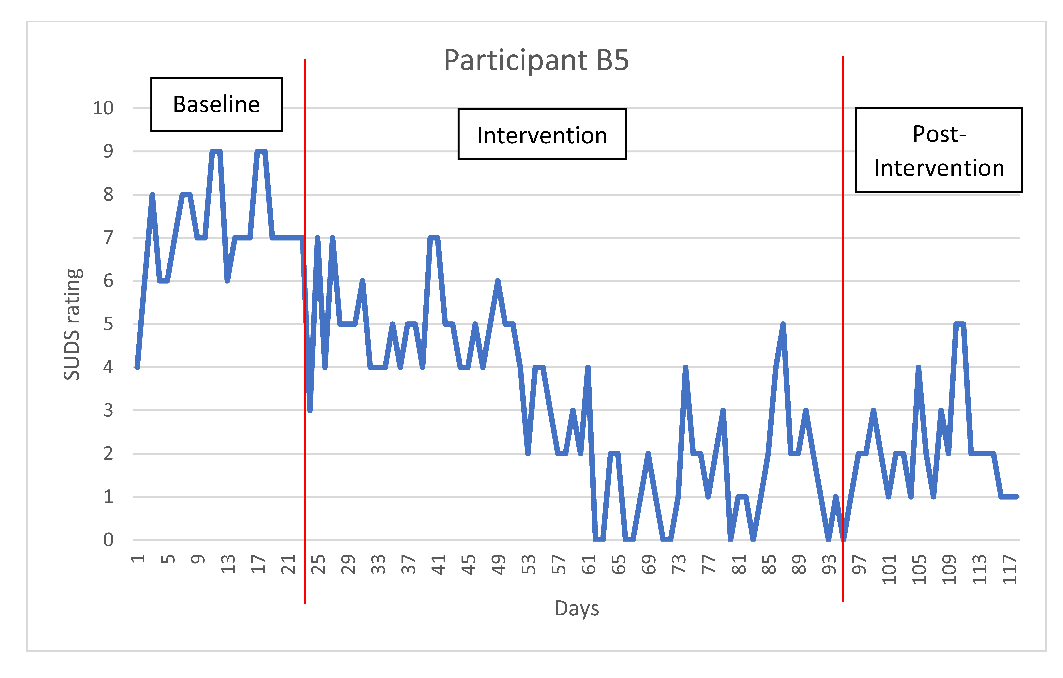
*SUDS Ratings for Participants B5 – B7 Using* Smiling Mind


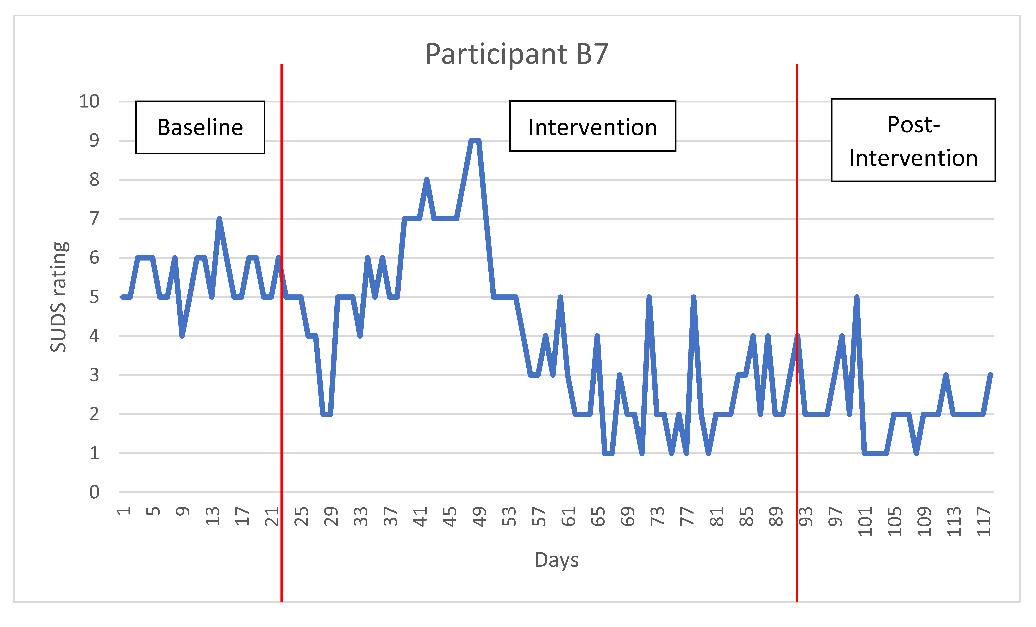


Supplementary Table 21

*SUDS Data Summary for Participants Using* MoodMission

| Participant |  | Baseline SUDS | Intervention SUDS | Post-intervention SUDS |
| --- | --- | --- | --- | --- |
| C1 | *M*  *SD*  Frequency (*n*) | 4.5  1.8  22 | 4.8  2.1  62 | 1.8  0.8  27 |
| C2 | *M*  *SD*  Frequency (*n*) | 7.3  0.9  26 | 5.6  1.2  63 | 4.6  0.9  25 |
| C3 | *M*  *SD*  Frequency (*n*) | 4.8  1.4  24 | 6.3  1.9  65 | 5.6  0.9  26 |
| C4 | *M*  *SD*  Frequency (*n*) | 4.1  1.5  25 | 3.2  1.6  67 | 2.6  0.9  27 |
| C5 | *M*  *SD*  Frequency (*n*) | 5.9  1.5  26 | 4.9  1.4  68 | 2.7  0.8  26 |
| C6 | *M*  *SD*  Frequency (*n*) | 3.6  1.0  25 | 3.2  1.2  67 | 2.5  1.4  26 |

**Supplementary Figure 9**


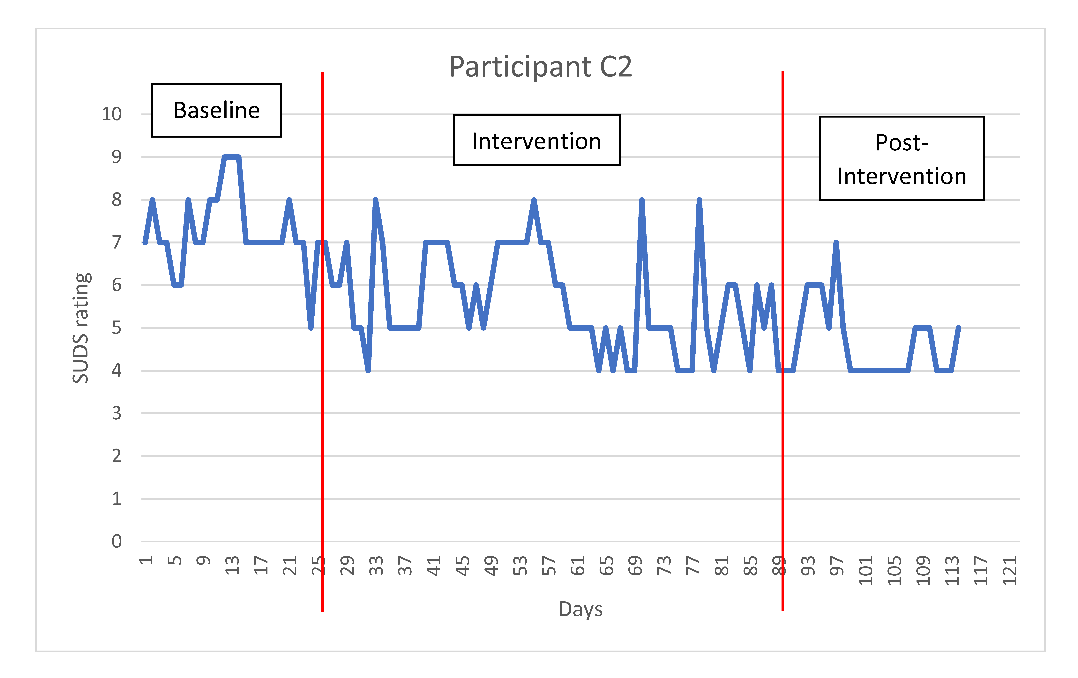
*SUDS Ratings for Participants C1 – C4 Using* MoodMission


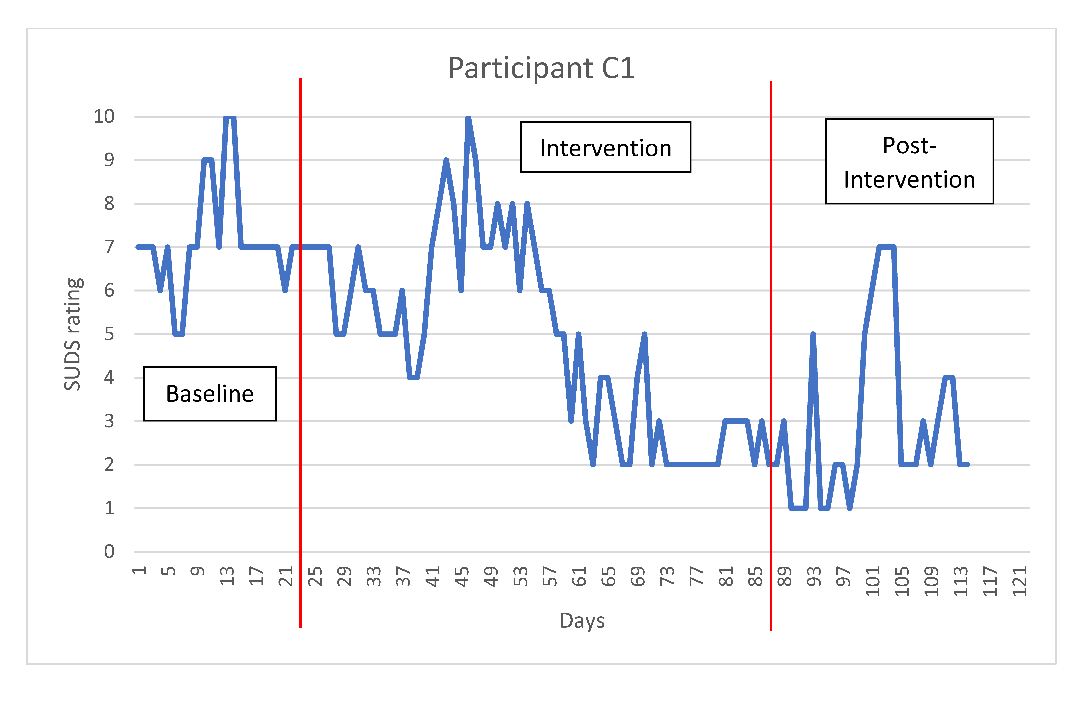

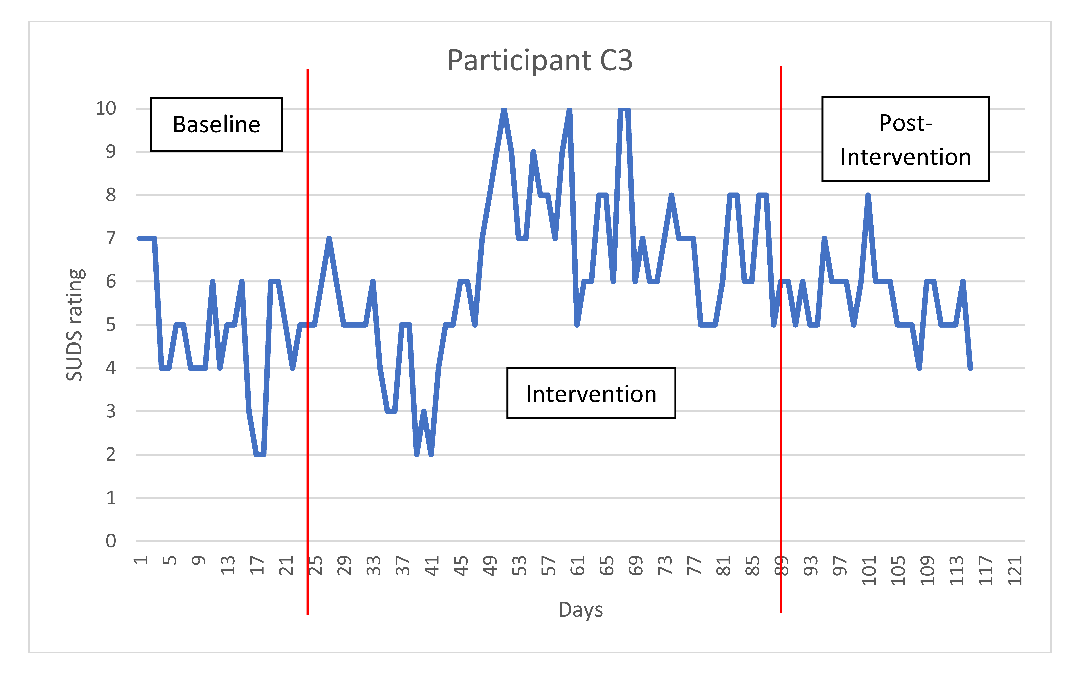

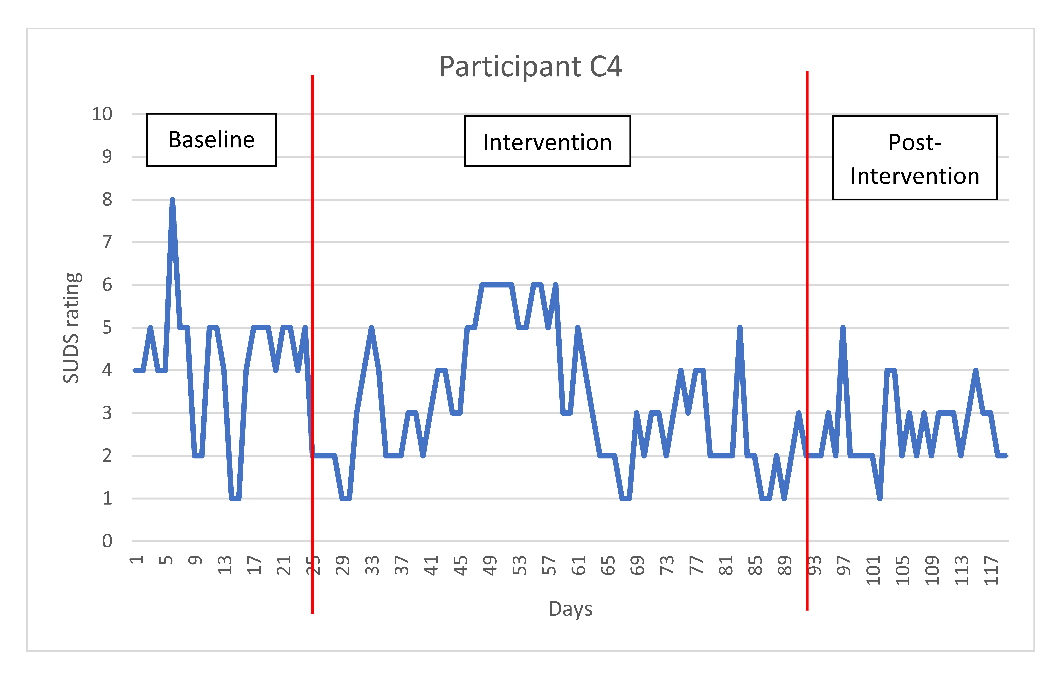


**Supplementary Figure 10**

*SUDS Ratings for Participants C5 – C6 Using* MoodMission


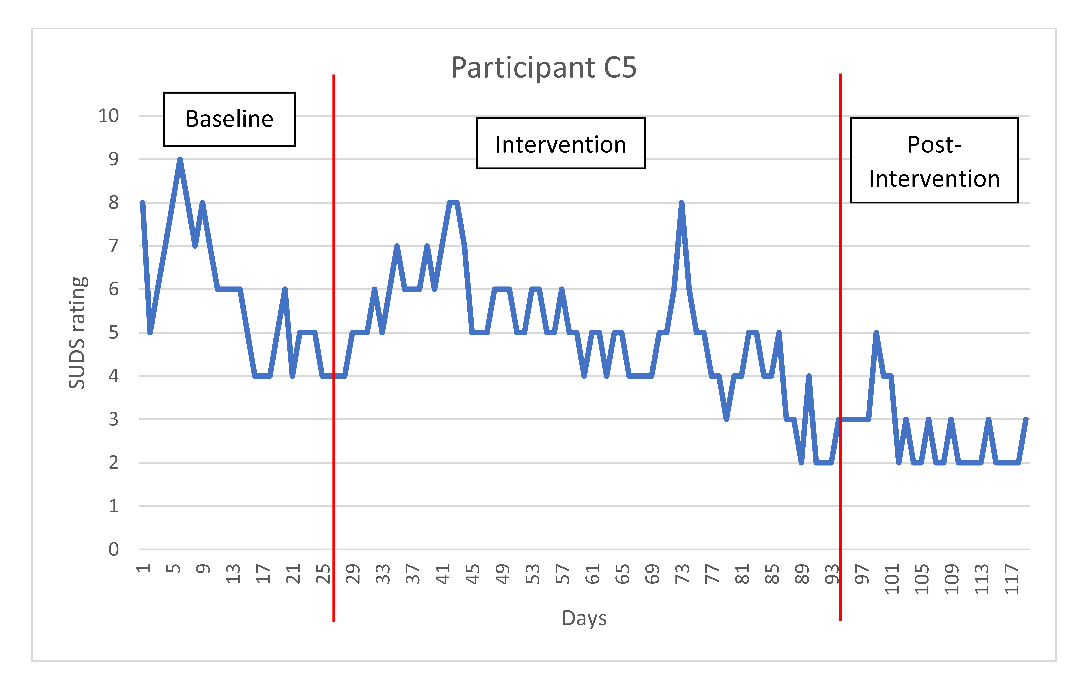

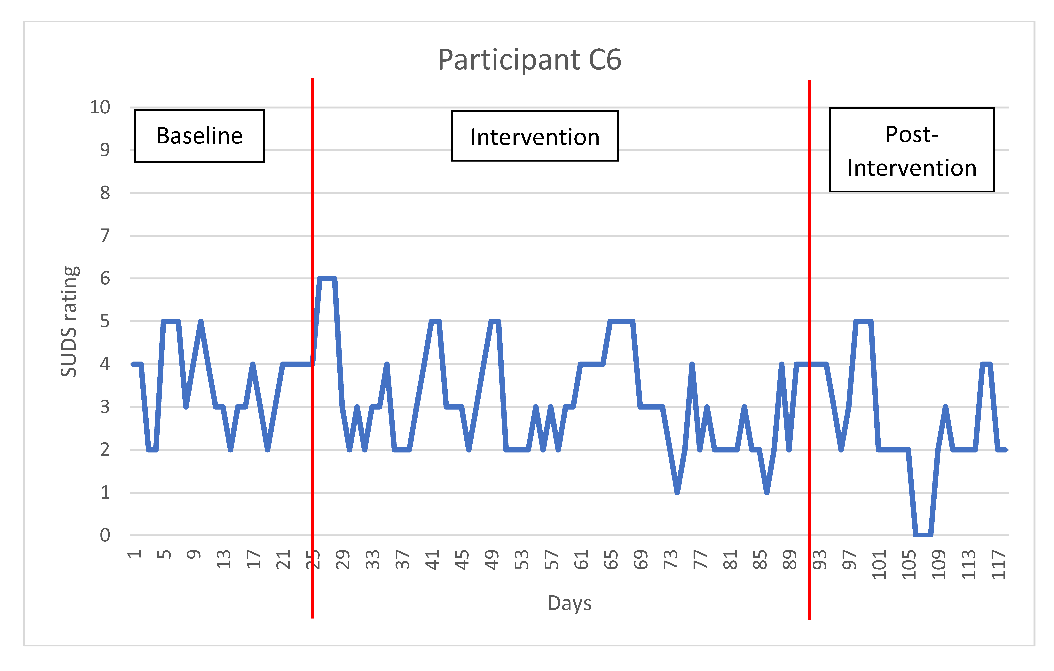


Supplementary Table 22

*SUDS Data Summary for Participants Using* MindShift

| Participant |  | Baseline SUDS | Intervention SUDS | Post-intervention SUDS |
| --- | --- | --- | --- | --- |
| D1 | *M*  *SD*  Frequency (*n*) | 4.8  1.5  20 | 5.2  1.8  64 | 3.3  1.0  26 |
| D2 | *M*  *SD*  Frequency (*n*) | 4.6  1.2  24 | 3.2  1.5  66 | 2.4  1.4  25 |
| D3 | *M*  *SD*  Frequency (*n*) | 7.2  1.3  23 | 4.6  1.5  64 | 3.0  2.0  27 |
| D4 | *M*  *SD*  Frequency (*n*) | 3.3  1.2  25 | 3.6  1.0  67 | 2.6  0.8  27 |
| D5 | *M*  *SD*  Frequency (*n*) | 5.7  1.4  20 | 6.6  1.4  63 | 5.2  1.2  27 |

**Supplementary Figure 11**

*SUDS Ratings for Participants D1 – D4 Using* MindShift


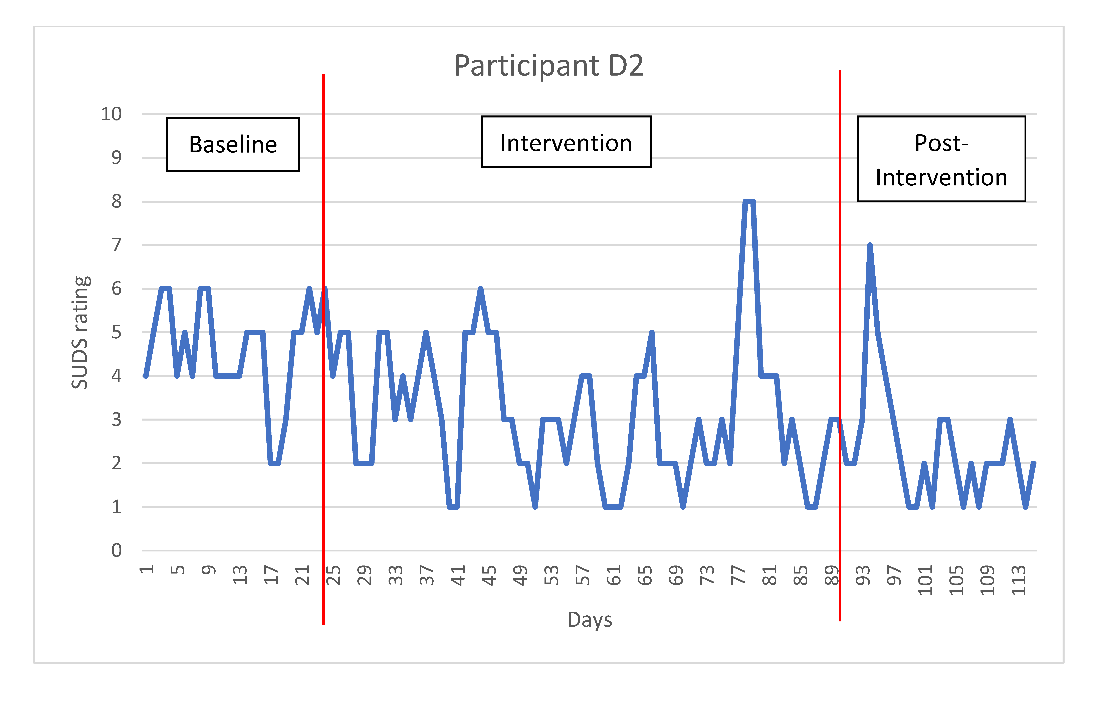

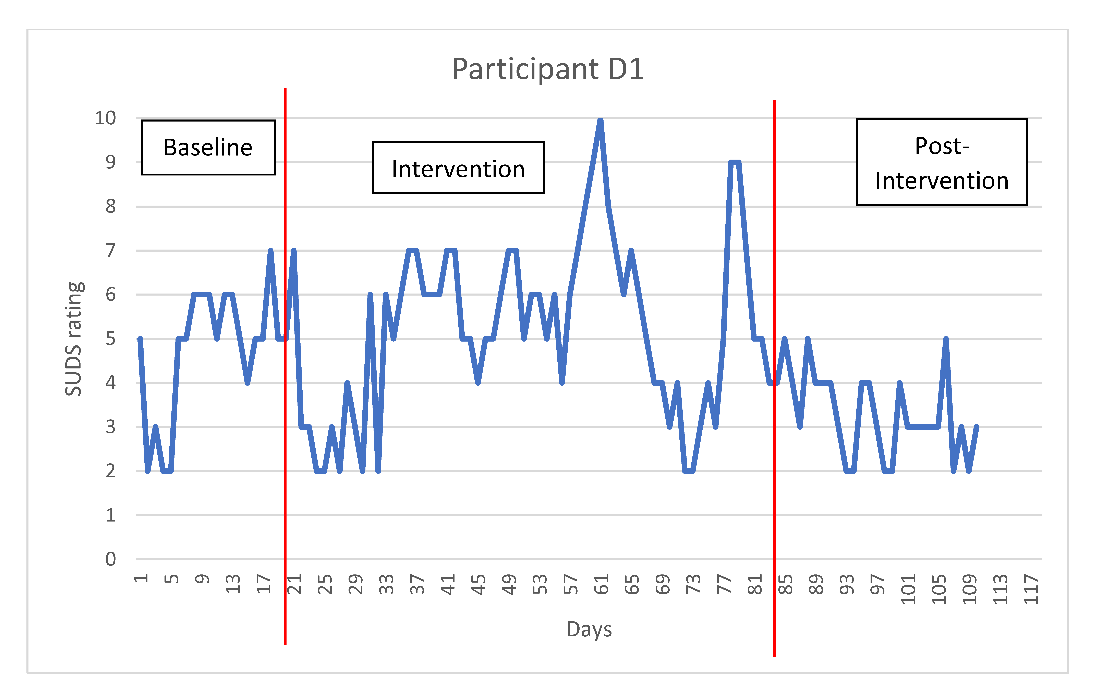

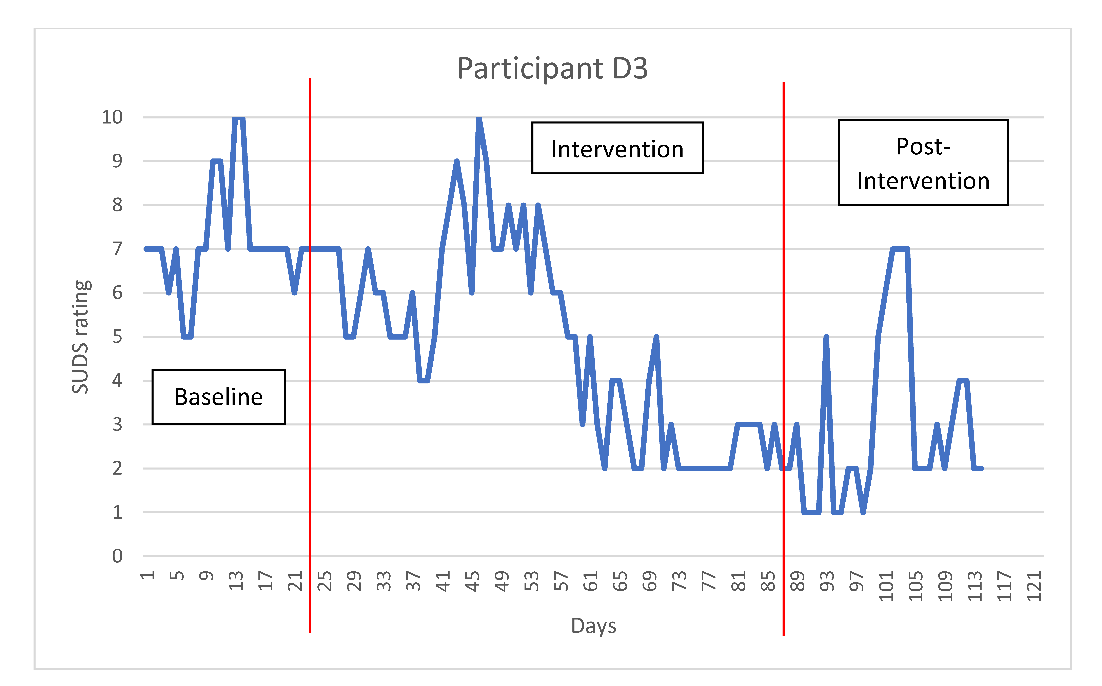

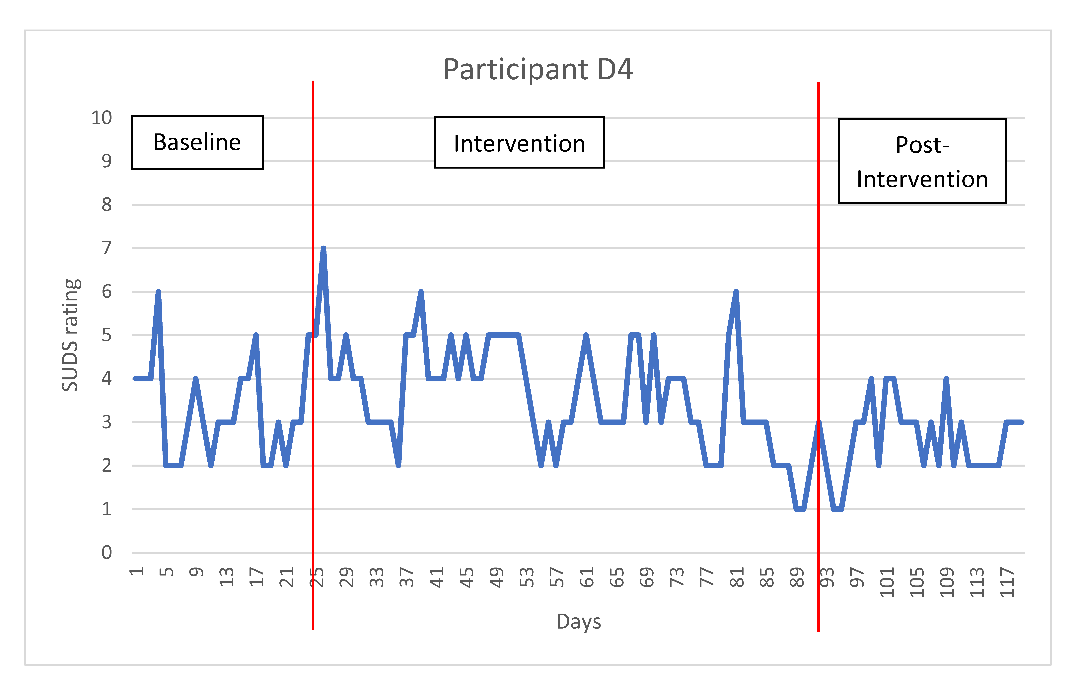


**Supplementary Figure 12**

*SUDS Ratings for Participant D5 Using* MindShift

*
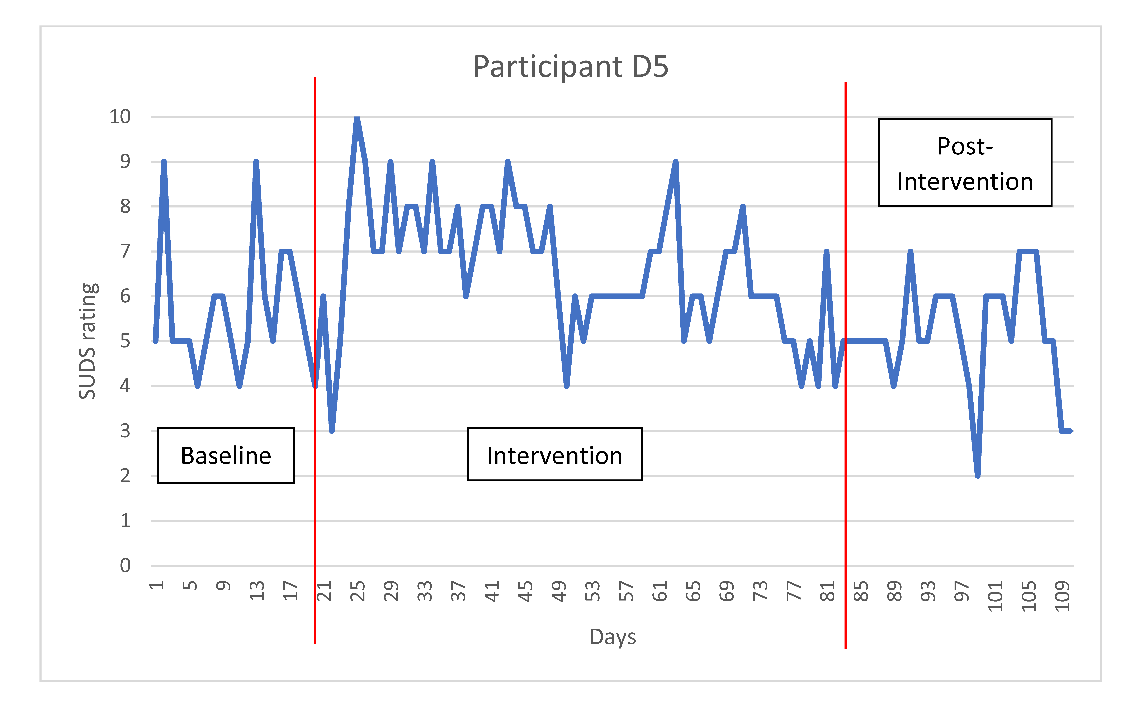
*

Supplementary Table 23

*SUDS Data Summary for Participants Using* Destressify

| Participant |  | Baseline SUDS | Intervention SUDS | Post-intervention SUDS |
| --- | --- | --- | --- | --- |
| E1 | *M*  *SD*  Frequency (*n*) | 2.9  1.2  22 | 2.2  1.3  61 | 1.6  0.9  27 |
| E2 | *M*  *SD*  Frequency (*n*) | 6.8  1.5  23 | 6.1  2.0  65 | 5.6  1.1  27 |
| E3 | *M*  *SD*  Frequency (*n*) | 4.3  1.0  22 | 4.8  1.3  68 | 4.1  1.1  25 |
| E4 | *M*  *SD*  Frequency (*n*) | 5.1  0.9  24 | 4.3  2.3  71 | 1.8  0.9  24 |
| E5 | *M*  *SD*  Frequency (*n*) | 7.1  1.1  22 | 6.2  1.7  68 | 4.7  0.8  26 |
| E6 | *M*  *SD*  Frequency (*n*) | 5.1  1.1  22 | 2.6  1.2  65 | 1.8  1.0  24 |

**Supplementary Figure 13**

*SUDS Ratings for Participants E1 – E4 Using* Destressify


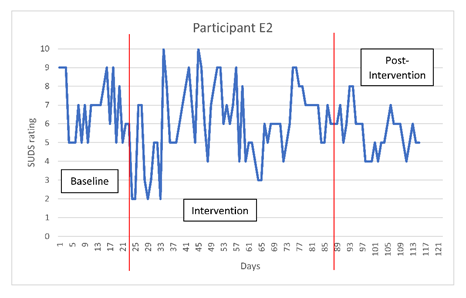

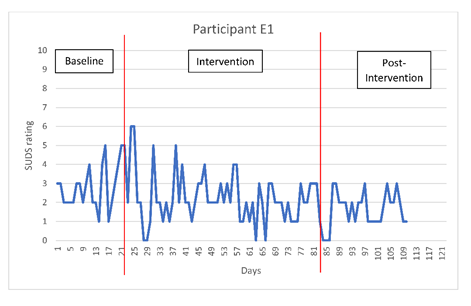

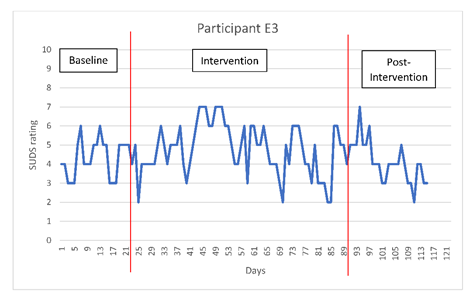

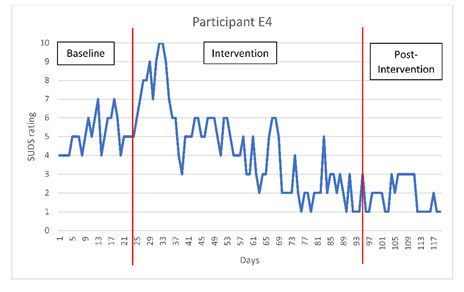


**Supplementary Figure 14**

*SUDS Ratings for Participants E5 – E6 Using* Destressify


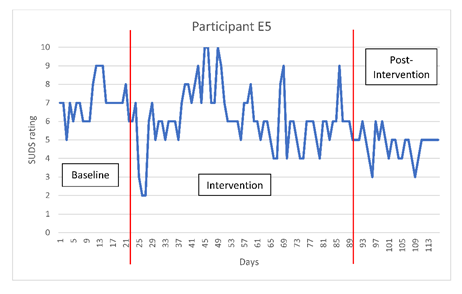

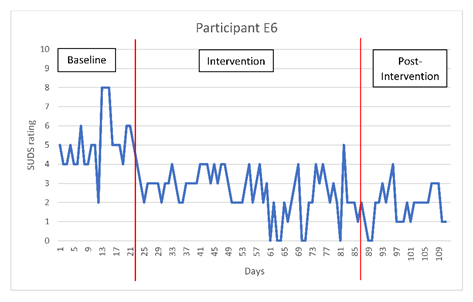


**Supplementary Figure 15**

*DASS-21 Anxiety Scores for Participants A1 – A5 Using* SuperBetter


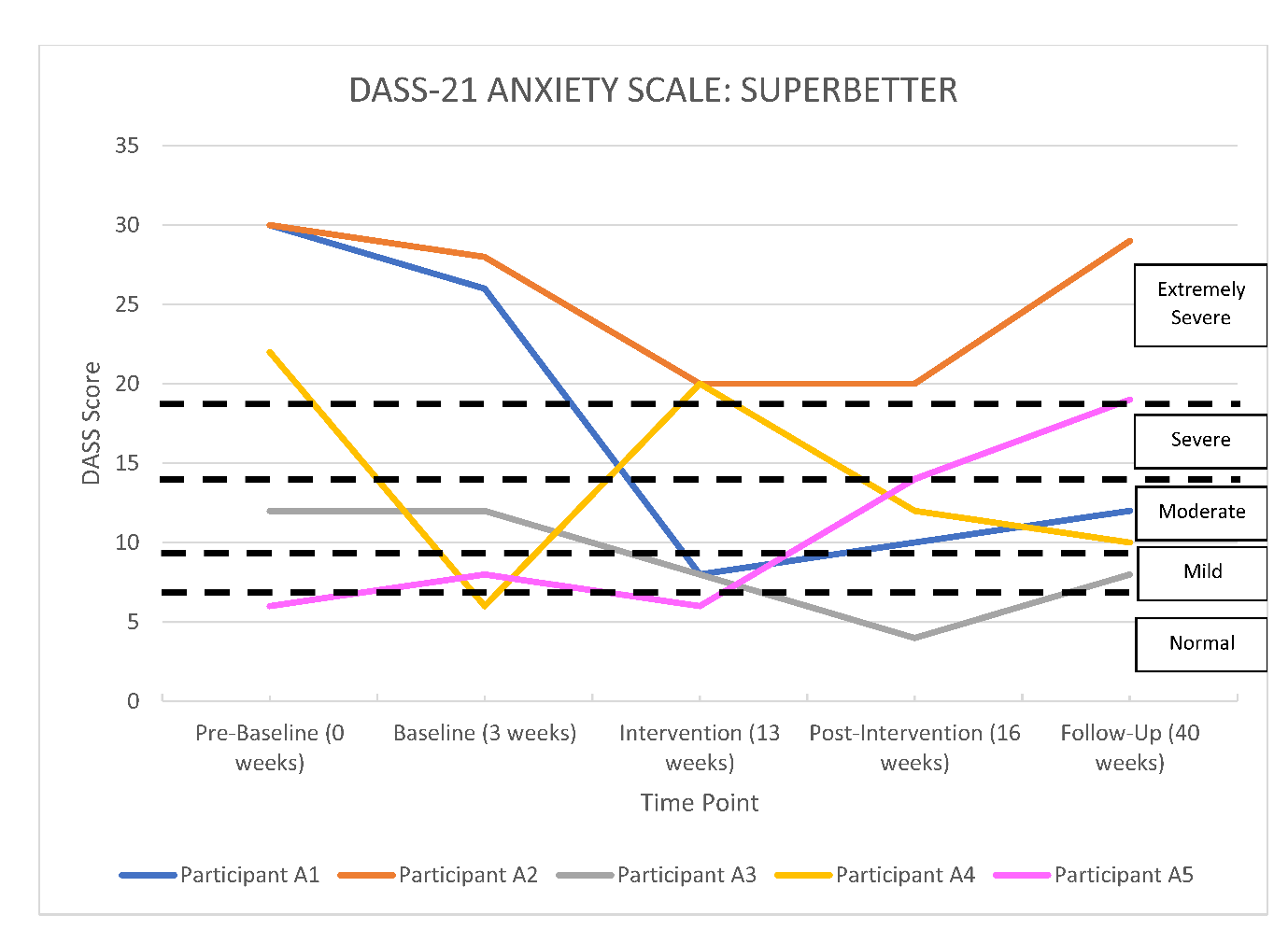


**Supplementary Figure 16**

*DASS-21 Anxiety Scores for Participants B1 – B7 Using* Smiling Mind


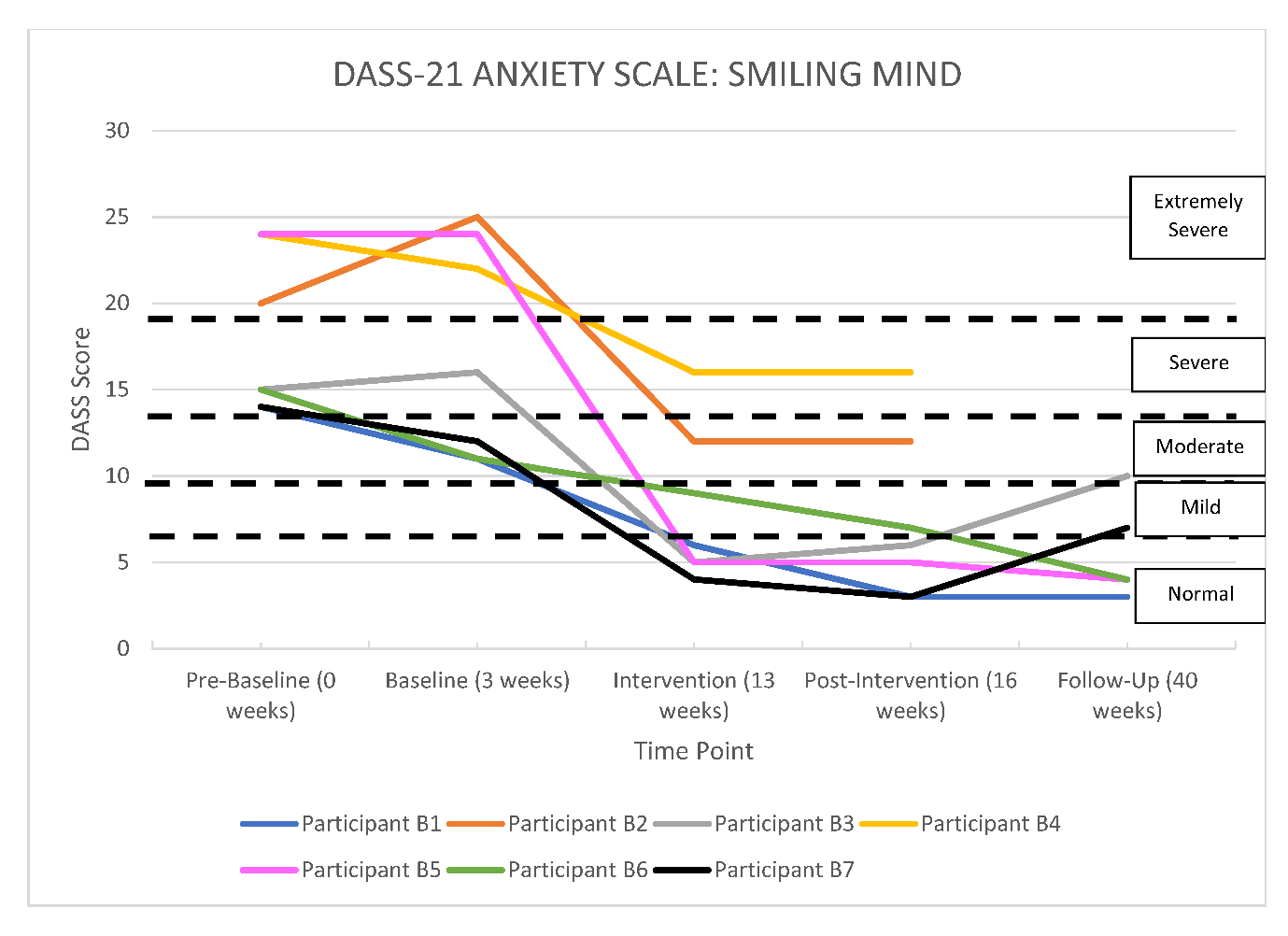


**Supplementary Figure 17**

*DASS-21 Anxiety Scores for Participants C1 – C6 Using* MoodMission


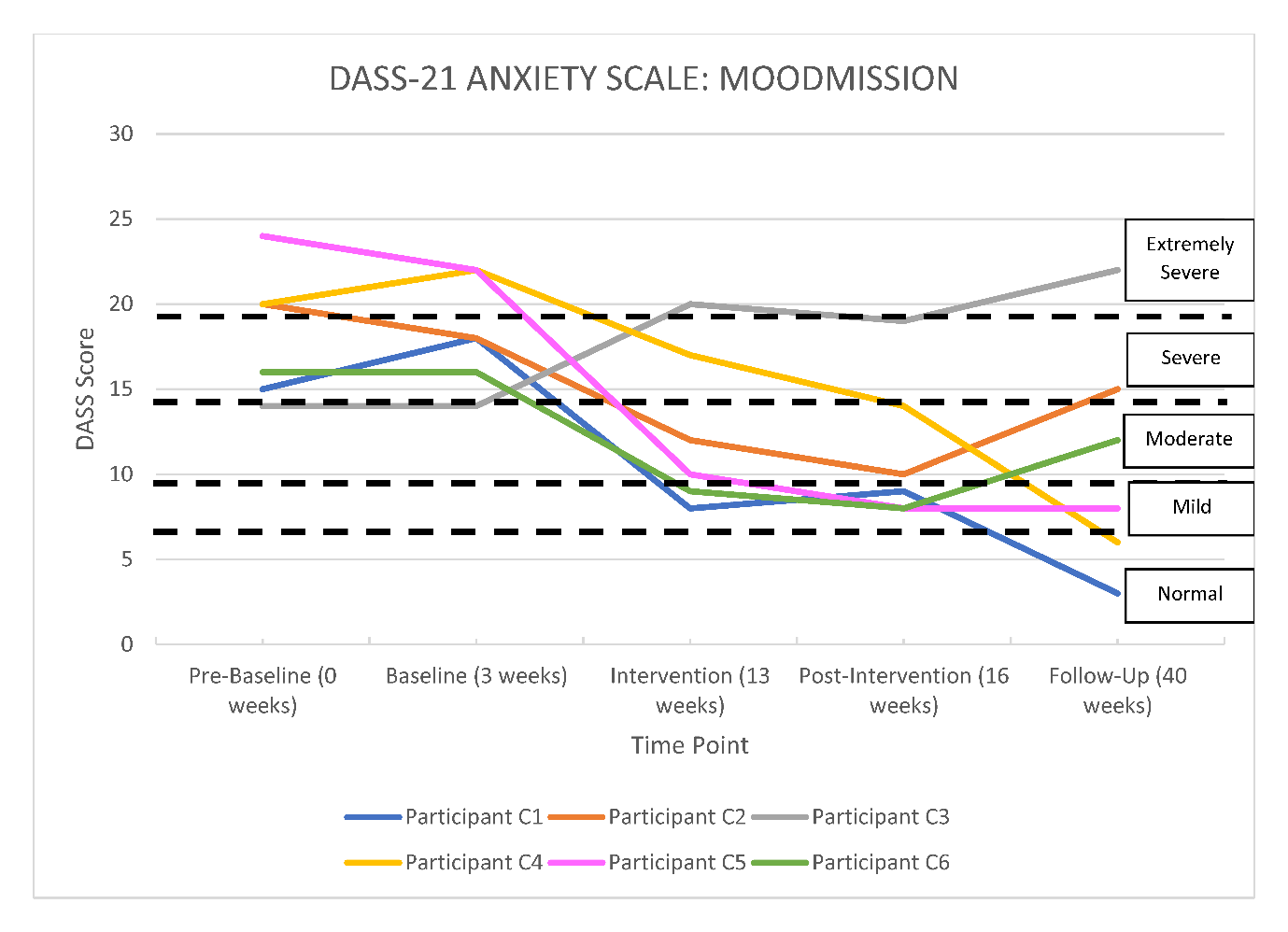


**Supplementary Figure 18**

*DASS-21 Anxiety Scores for Participants D1 – D5 Using* MindShift


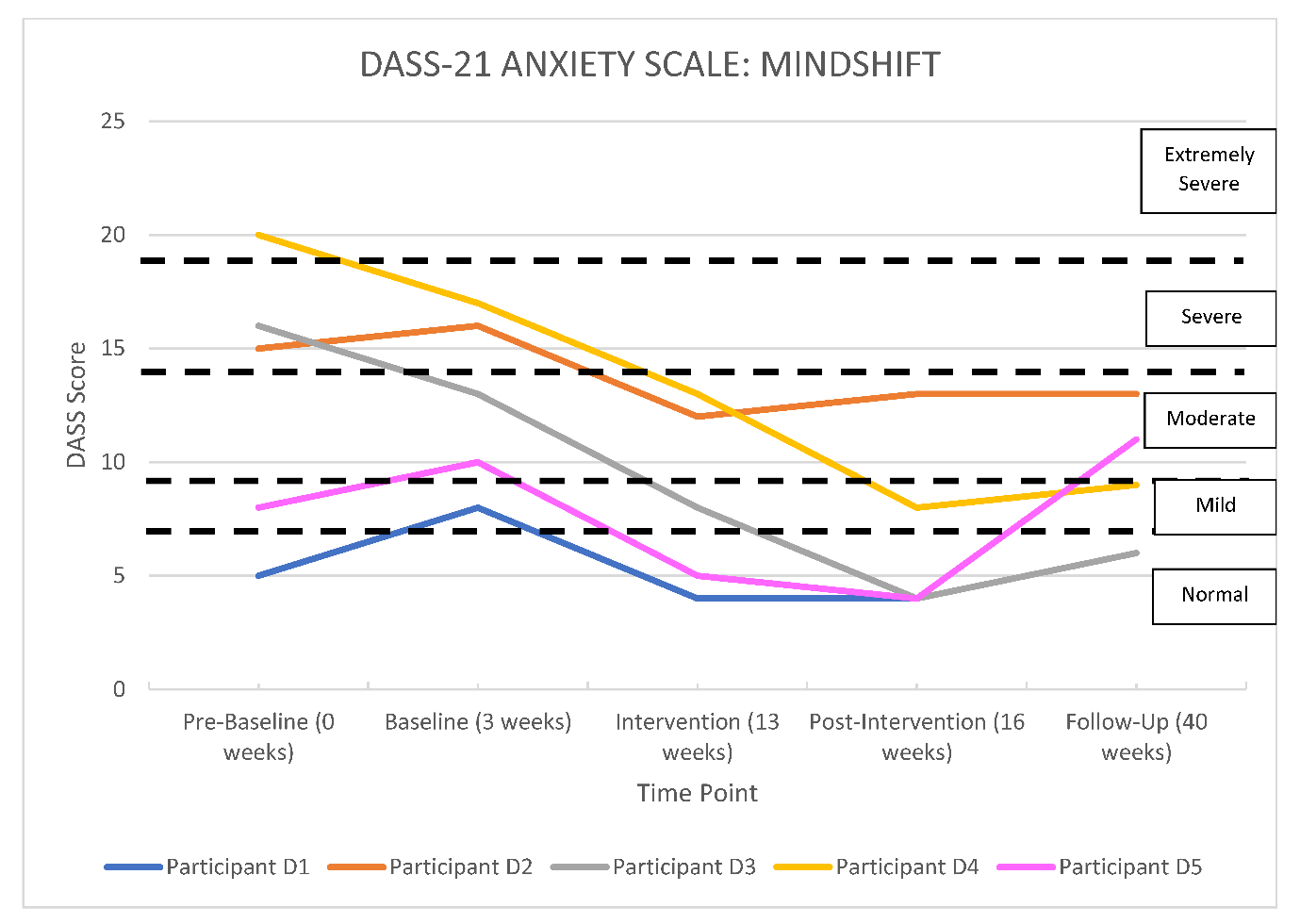


**Supplementary Figure 19**

*DASS-21 Anxiety Scores for Participants E1 – E6 Using* Destressify


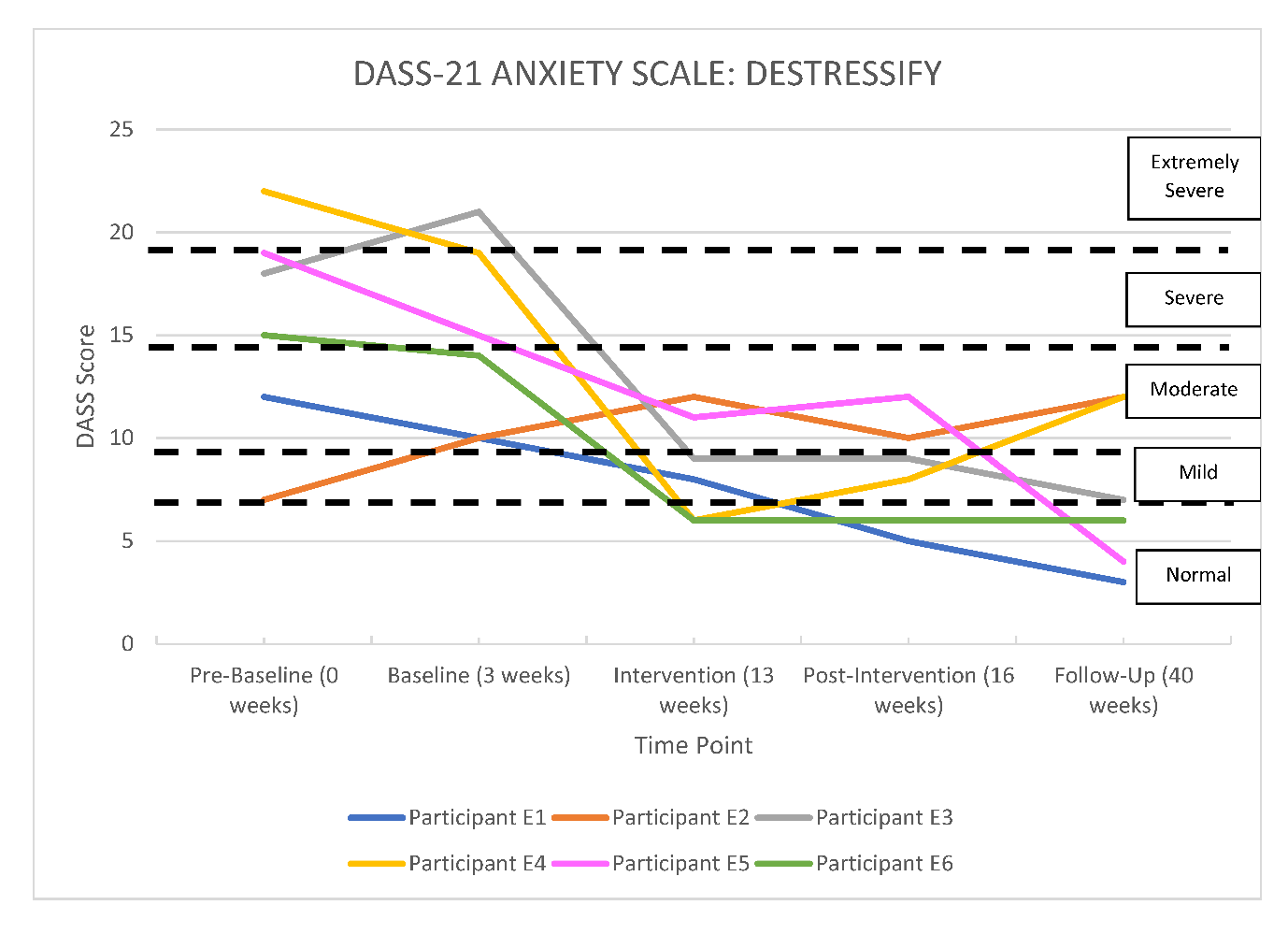


**Supplementary Figure 20**

*DASS-21 Depression Scores for Participants A1 – A5 Using* SuperBetter


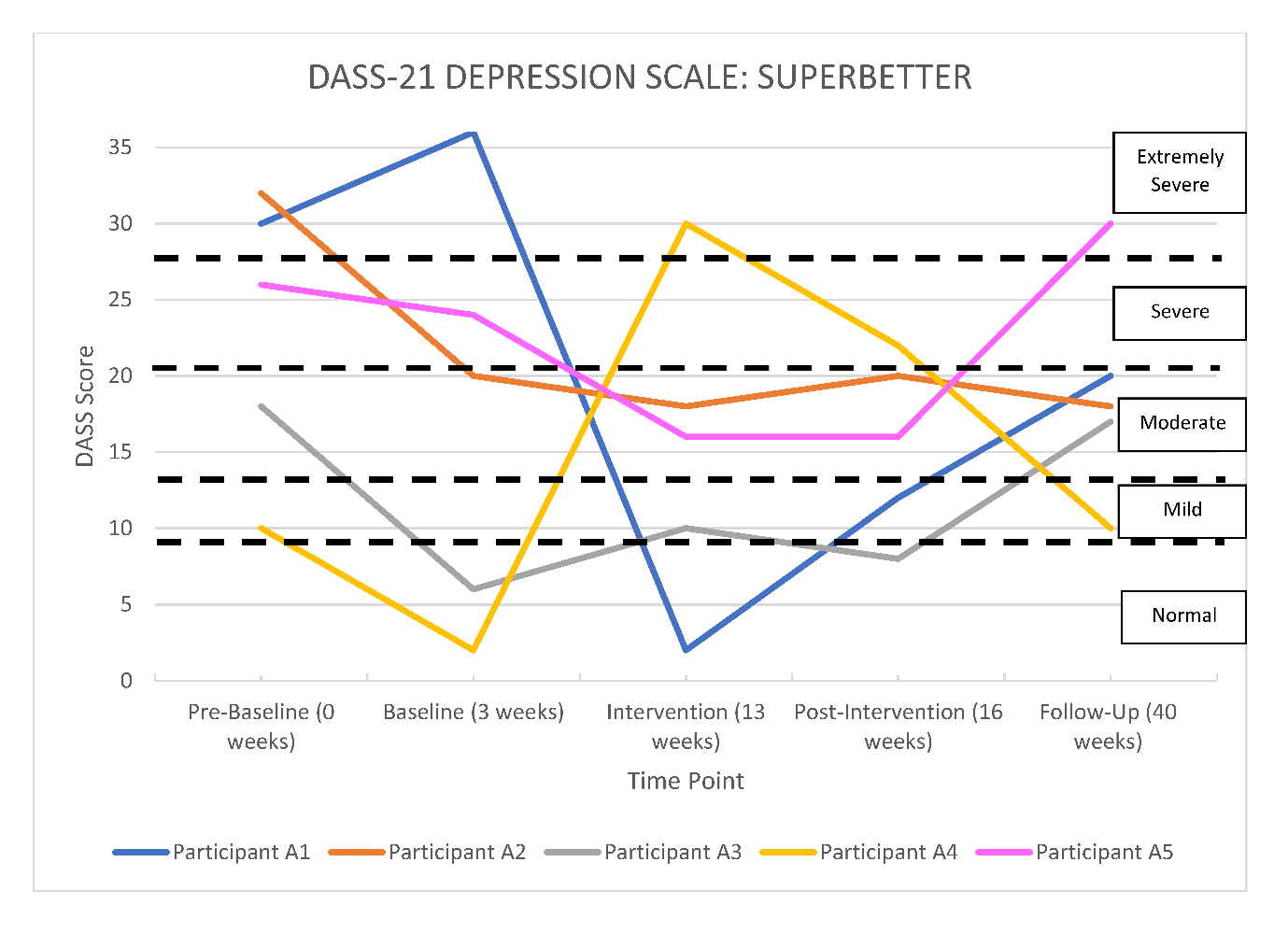


**Supplementary Figure 21**

*DASS-21 Depression Scores for Participants B1 – B7 Using* Smiling Mind


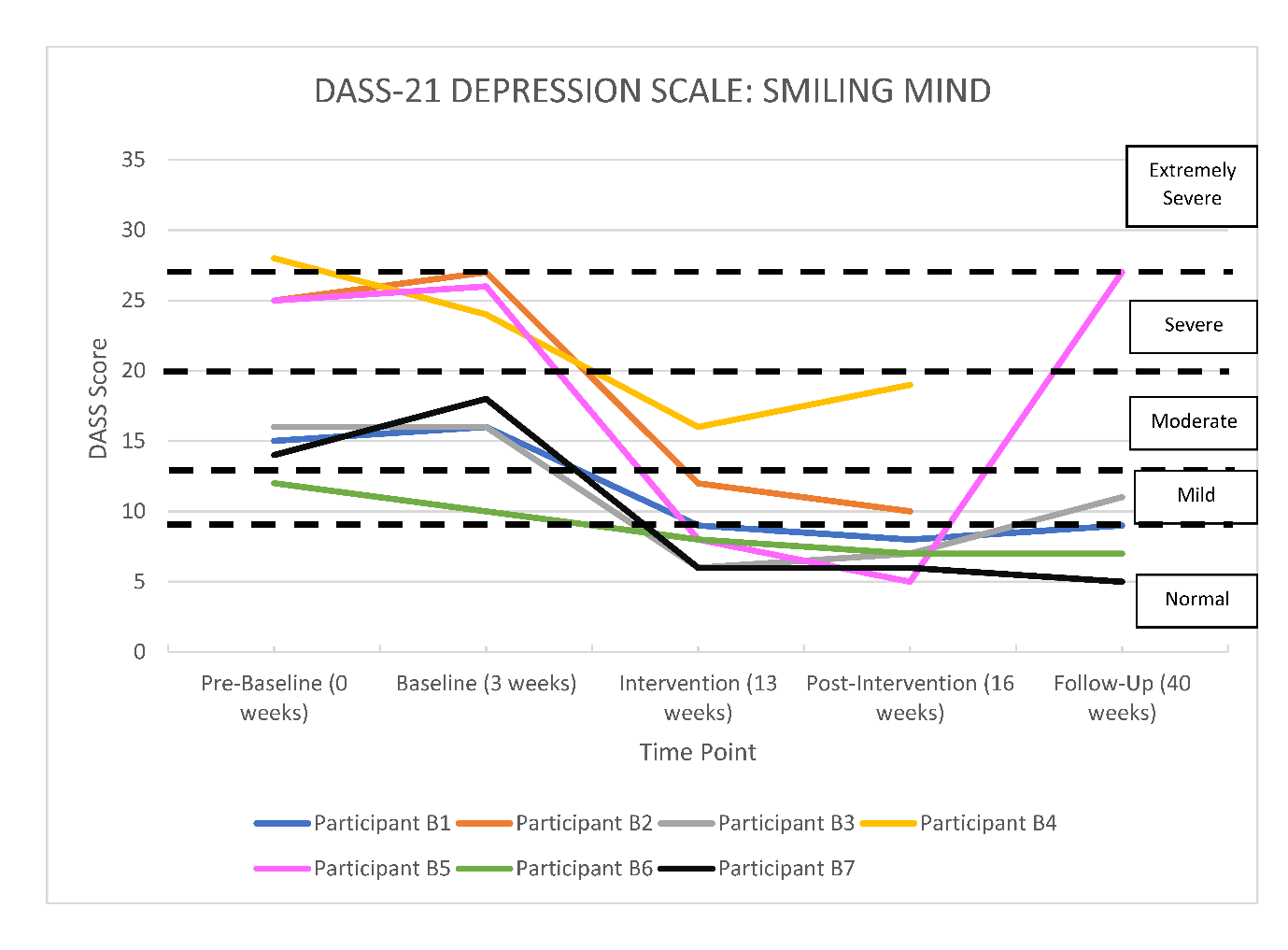


**Supplementary Figure 22**

*DASS-21 Depression Scores for Participants C1 – C6 Using MoodMission*


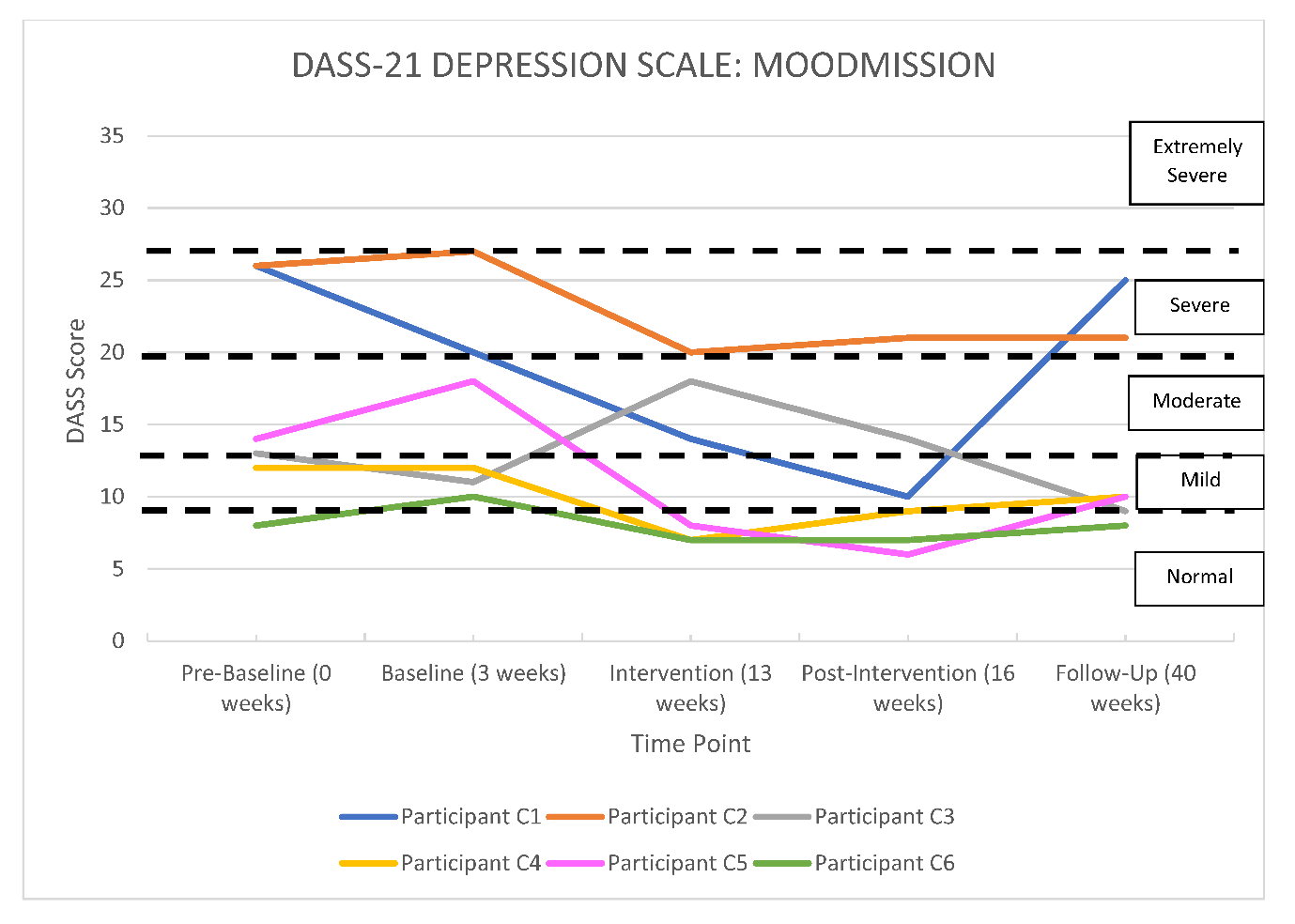


**Supplementary Figure 23**

*DASS-21 Depression Scores for Participants D1 – D5 Using* MindShift


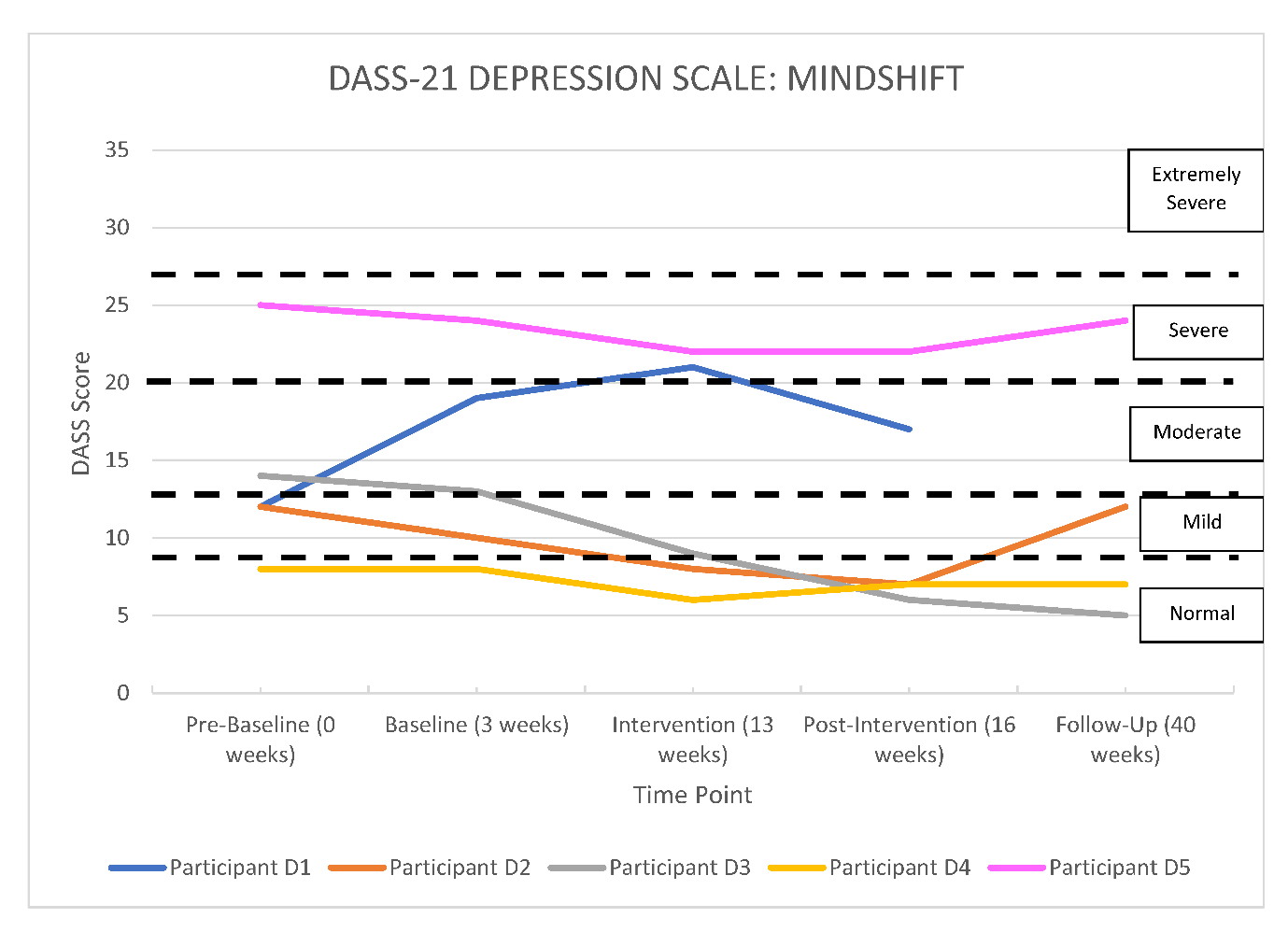


**Supplementary Figure 24**

*DASS-21 Depression Scores for Participants E1 – E6 Using* Destressify


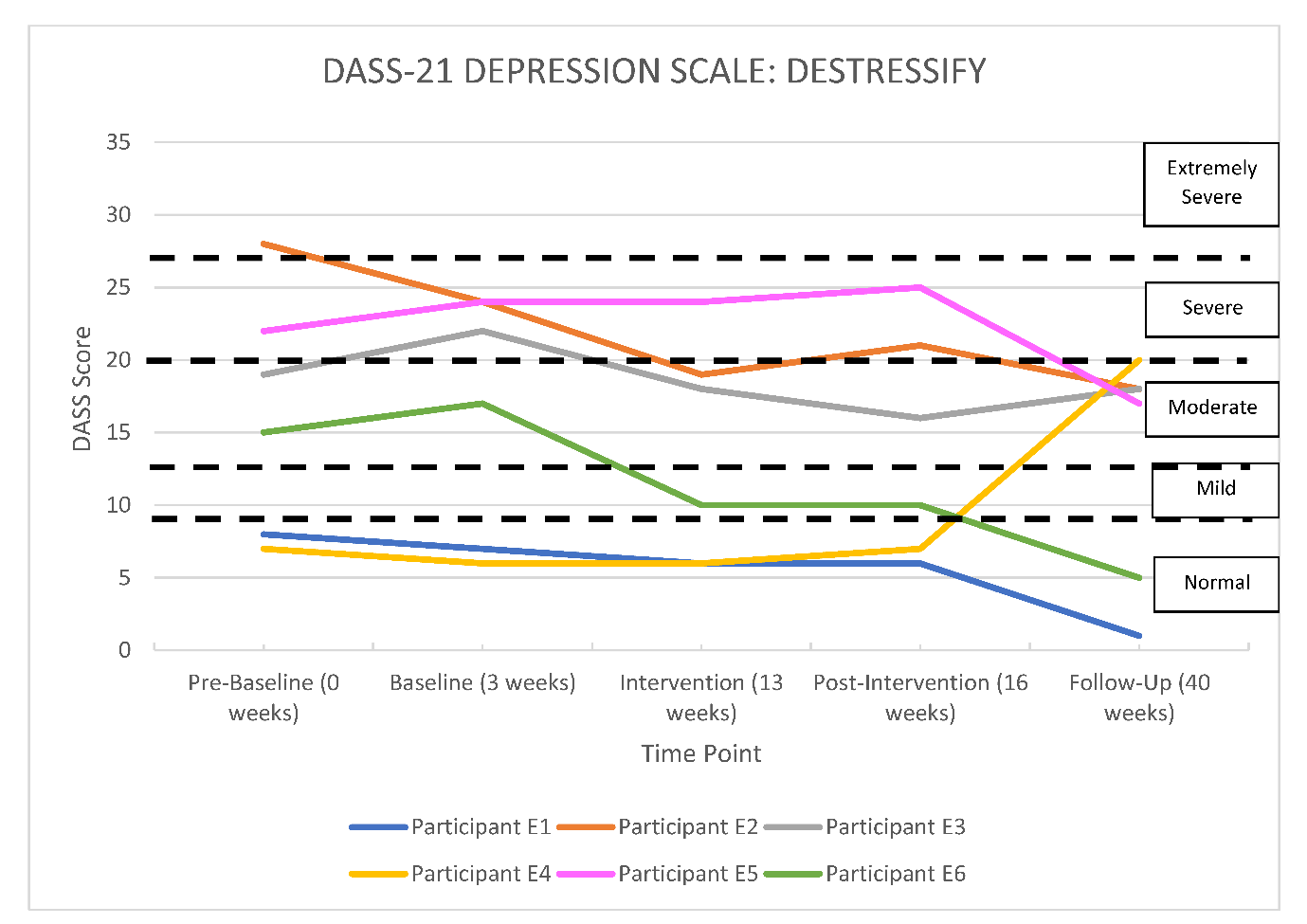


**Supplementary Table 24**

*OQ-45.2 Total Scores for Participants A1 – A5 Using* SuperBetter

| Participant | Pre-baseline to post-baseline | Post-baseline to post-intervention | Post-intervention to post-3-week follow up | Overall (pre-baseline to post-3-week follow up) | Classification (pre-baseline to post-3-week follow up) | Improvement (pre-baseline to post-3-week follow up) |
| --- | --- | --- | --- | --- | --- | --- |
| A1 | 99-96 ^ | 96-37 *+ | 37-32 ^ | 99-32 *+ | Recovered | 67.68% |
| A2 | 115-90 * | 90-83 ^ | 83-74 ^ | 115-74 * | Improved | 35.65% |
| A3 | 84-74 ^ | 74-55 *+ | 55-55 ^ | 84-55 *+ | Recovered | 34.52% |
| A4 | 69-49 *+ | (49-91) *+ | 91-87 ^ | (69-87) * | Deteriorated | 26.09% |
| A5 | (104-117) ^ | 117-83 * | 83-82 ^ | 104-82 * | Improved | 21.15% |

*Note*. Based on RCI = 14 and CSI = 63 for Total Score. ^ denotes not statistically reliable, not clinically significant; * denotes statistically reliable (*p* < .05); + denotes clinically significant; *+ denotes statistically reliable and clinically significant. Parentheses indicate change in a worsening direction. *Recovered* = clinically significant and statistically reliable; *Improved* = not clinically significant, but statistically reliable; *Unchanged* = not clinically significant or statistically reliable; *Deteriorated* = clinically significant and/or statistically reliable in a worsening direction.

**Supplementary Table 25**

*OQ-45.2 Total Scores for Participants B1 – B7 Using* Smiling Mind

| Participant | Pre-baseline to post-baseline | Post-baseline to post-intervention | Post-intervention to post-3-week follow up | Overall (pre-baseline to post-3-week follow up) | Classification (pre-baseline to post-3-week follow up) | Improvement (pre-baseline to post-3-week follow up) |
| --- | --- | --- | --- | --- | --- | --- |
| B1 | (85-89) ^ | 89-40 *+ | (40-42) ^ | 85-42 *+ | Recovered | 50.59% |
| B2 | (105-110) ^ | 110-59 *+ | 59-52 ^ | 105-52 *+ | Recovered | 50.47% |
| B3 | (80-88) ^ | 88-35 *+ | 35-33 ^ | 80-33 *+ | Recovered | 58.75% |
| B4 | 116-110 ^ | 110-100 ^ | (100-102) ^ | 116-102 * | Improved | 12.07% |
| B5 | (111-114) ^ | 114-38 *+ | 38-33 ^ | 111-33 *+ | Recovered | 70.27% |
| B6 | (57-60) ^ | (60-65) + | 65-45 *+ | 57-45 * | Unchanged ^a^ | 21.05% |
| B7 | 76-72 ^ | 72-50 *+ | (50-56) ^ | 76-56 *+ | Recovered | 26.32% |

*Note*. Based on RCI = 14 and CSI = 63 for Total Score. ^ denotes not statistically reliable, not clinically significant; * denotes statistically reliable (*p* < .05); + denotes clinically significant; *+ denotes statistically reliable and clinically significant. Parentheses indicate change in a worsening direction. *Recovered* = clinically significant and statistically reliable; *Improved* = not clinically significant, but statistically reliable; *Unchanged* = not clinically significant or statistically reliable; *Deteriorated* = clinically significant and/or statistically reliable in a worsening direction.

^a^ Non-clinical to begin with.

**Supplementary Table 26**

*OQ-45.2 Total Scores for Participants C1 – C6 Using* MoodMission

| Participant | Pre-baseline to post-baseline | Post-baseline to post-intervention | Post-intervention to post-3-week follow up | Overall (pre-baseline to post-3-week follow up) | Classification (pre-baseline to post-3-week follow up) | Improvement (pre-baseline to post-3-week follow up) |
| --- | --- | --- | --- | --- | --- | --- |
| C1 | 108-100 ^ | 100-62 *+ | 62-48 * | 108-48 *+ | Recovered | 55.56% |
| C2 | (110-111) ^ | 111-78 * | 78-78 ^ | 110-78 * | Improved | 29.09% |
| C3 | 53-50 ^ | (50-63) + | 63-61 ^ | (53-61) ^ | Unchanged ^a^ | 15.09% |
| C4 | 67-67 ^ | 67-59 + | 59-52 ^ | 67-52 *+ | Recovered | 22.39% |
| C5 | 87-86 ^ | 86-47 *+ | 47-44 ^ | 87-44 *+ | Recovered | 49.43% |
| C6 | 74-70 ^ | 70-52 *+ | 52-46 ^ | 74-46 *+ | Recovered | 37.84% |

*Note*. Based on RCI = 14 and CSI = 63 for Total Score. ^ denotes not statistically reliable, not clinically significant; * denotes statistically reliable (*p* < .05); + denotes clinically significant; *+ denotes statistically reliable and clinically significant. Parentheses indicate change in a worsening direction. *Recovered* = clinically significant and statistically reliable; *Improved* = not clinically significant, but statistically reliable; *Unchanged* = not clinically significant or statistically reliable; *Deteriorated* = clinically significant and/or statistically reliable in a worsening direction.

^a^ Non-clinical to begin with.

**Supplementary Table 27**

*OQ-45.2 Total Scores for Participants D1 – D5 Using* MindShift

| Participant | Pre-baseline to post-baseline | Post-baseline to post-intervention | Post-intervention to post-3-week follow up | Overall (pre-baseline to post-3-week follow up) | Classification (pre-baseline to post-3-week follow up) | Improvement (pre-baseline to post-3-week follow up) |
| --- | --- | --- | --- | --- | --- | --- |
| D1 | (64-72) ^ | 72-68 ^ | 68-54 *+ | 64-54 + | Unchanged | 15.63% |
| D2 | 63-56 + | 56-43 ^ | (43-44) ^ | 63-44 *+ | Recovered | 30.16% |
| D3 | 85-72 ^ | 72-50 *+ | 50-38 ^ | 85-38 *+ | Recovered | 55.29% |
| D4 | 66-61 + | 61-48 ^ | (48-54) ^ | 66-54 + | Unchanged | 18.18% |
| D5 | 96-87 ^ | 87-74 ^ | 74-66 ^ | 96-66 * | Improved | 31.25% |

*Note*. Based on RCI = 14 and CSI = 63 for Total Score. ^ denotes not statistically reliable, not clinically significant; * denotes statistically reliable (*p* < .05); + denotes clinically significant; *+ denotes statistically reliable and clinically significant. Parentheses indicate change in a worsening direction. *Recovered* = clinically significant and statistically reliable; *Improved* = not clinically significant, but statistically reliable; *Unchanged* = not clinically significant or statistically reliable; *Deteriorated* = clinically significant and/or statistically reliable in a worsening direction.

**Supplementary Table 28**

*OQ-45.2 Total Scores for Participants E1 – E6 Using* Destressify

| Participant | Pre-baseline to post-baseline | Post-baseline to post-intervention | Post-intervention to post-3-week follow up | Overall (pre-baseline to post-3-week follow up) | Classification (pre-baseline to post-3-week follow up) | Improvement (pre-baseline to post-3-week follow up) |
| --- | --- | --- | --- | --- | --- | --- |
| E1 | (44-51) ^ | 51-33 * | (33-37) ^ | 44-37 ^ | Unchanged ^a^ | 15.91% |
| E2 | 105-105 ^ | 105-96 ^ | 96-83 ^ | 105-83 * | Improved | 20.95% |
| E3 | 76-75 ^ | 75-57 *+ | (57-63) + | 76-63 ^ | Unchanged | 17.11% |
| E4 | 107-95 ^ | 95-92 ^ | 92-71 * | 107-71 * | Improved | 33.65% |
| E5 | 78-78 ^ | 78-67 ^ | 67-66 ^ | 78-66 ^ | Unchanged | 15.39% |
| E6 | (73-74) ^ | 74-60 *+ | 60-55 ^ | 73-55 *+ | Recovered | 24.66% |

*Note*. Based on RCI = 14 and CSI = 63 for Total Score. ^ denotes not statistically reliable, not clinically significant; * denotes statistically reliable (*p* < .05); + denotes clinically significant; *+ denotes statistically reliable and clinically significant. Parentheses indicate change in a worsening direction. *Recovered* = clinically significant and statistically reliable; *Improved* = not clinically significant, but statistically reliable; *Unchanged* = not clinically significant or statistically reliable; *Deteriorated* = clinically significant and/or statistically reliable in a worsening direction.

^a^ Non-clinical to begin with.

**Supplementary Table 29**

*Classification of Participant Improvements Across Daily Distress, Symptomatology, and Life Functioning*

| Participant | SUDS | Depression | DASS-42 Anxiety | Stress | Total Score | OQ-45.2 Symptom Distress | Interpersonal Relations | Social Role |
| --- | --- | --- | --- | --- | --- | --- | --- | --- |
| A1 | Mod | High | High | Mod | High | High | Less | Less |
| A2 | High | Mod | Mod | Mod | High | Mod | Mod | High |
| A3 | Less | High | High | Less | High | High | Less | Less |
| A4 | Less | Less | Mod | Less | Less | Less | Mod | Less |
| A5 | Mod | Mod | Less | Mod | Mod | Less | Mod | Less |
| B1 | High | High | High | High | High | High | Less | Mod |
| B2 | High | High | Mod | High | High | High | Less | Less |
| B3 | Mod | High | High | High | High | High | Mod | Mod |
| B4 | High | Mod | Mod | Less | Mod | Less | Less | Mod |
| B5 | High | High | High | High | High | High | High | High |
| B6 | Less | Less | Mod | Mod | Mod | Less | Less | Less |
| B7 | High | Mod | High | High | High | Mod | Less | Less |
| C1 | High | Mod | Less | Mod | High | High | Mod | Mod |
| C2 | Mod | Less | Mod | Less | Mod | Mod | Mod | Less |
| C3 | Less | Less | Less | Less | Less | Less | Less | Less |
| C4 | Mod | Less | Less | Less | High | Mod | Less | Less |
| C5 | High | Mod | Mod | Mod | High | High | Mod | Mod |
| C6 | Less | Less | Mod | Less | High | High | Less | Less |
| D1 | Less | Less | Less | Less | Less | Less | Less | Less |
| D2 | Mod | Less | Less | Less | High | Less | Less | Mod |
| D3 | High | Mod | Mod | Mod | High | High | Mod | Mod |
| D4 | Less | Less | Mod | Less | Less | Mod | Less | Less |
| D5 | Less | Less | Less | Less | Mod | Mod | Less | Less |
| E1 | Less | Less | Mod | Mod | Less | Mod | Less | Less |
| E2 | Less | Mod | Less | Less | Mod | Mod | Less | Less |
| E3 | Less | Less | Mod | Mod | Mod | Mod | Less | Less |
| E4 | High | Less | Mod | High | Mod | Mod | Mod | Mod |
| E5 | Less | Less | Mod | Less | Mod | Less | Less | Less |
| E6 | High | Less | Mod | Mod | High | High | Less | Less |

*Note*. High = highly effective, Mod = moderately effective, and Less = less effective. Classification of each participant into an effectiveness group is based on criteria set out in Table A.1 above and author consensus after visually studying the various Tables and Figures of daily distress (SUDS), symptomatology (DASS), life functioning (OQ-45), and clinical significance and statistically reliable improvements.

**Supplementary Table 30**

*Participant App Ratings From the uMARS Questionnaire*

| Participant | Section A – Engagement  (out of 25) | Section B – Functionality  (out of 20) | Section C – Aesthetics  (out of 15) | Section D – Information  (out of 20) | Section E – App subjective quality  (out of 20) | Section F – Perceived impact  (out of 30) | TOTAL  (out of 130) | STAR RATING (out of 5) |
| --- | --- | --- | --- | --- | --- | --- | --- | --- |
| A1 | 18 | 17 | 9 | 9 | 6 | 14 | 73 | 2 |
| A2 | 15 | 13 | 14 | 13 | 12 | 30 | 97 | 3 |
| A3 | 16 | 14 | 8 | 17 | 11 | 21 | 87 | 3 |
| A4 | 17 | 12 | 11 | 12 | 7 | 15 | 74 | 3 |
| A5 | 11 | 12 | 9 | 13 | 7 | 15 | 67 | 2 |
| *SuperBetter M* | 15.40 | 13.60 | 10.20 | 12.80 | 8.60 | 19.00 | 79.60 | 2.60 |
| *SuperBetter SD* | 2.70 | 2.07 | 2.39 | 2.86 | 2.70 | 6.75 | 12.16 | 0.55 |
| B1 | 21 | 18 | 13 | 17 | 15 | 22 | 106 | 4 |
| B2 | 19 | 17 | 12 | 17 | 18 | 28 | 111 | 5 |
| B3 | 16 | 17 | 12 | 16 | 13 | 23 | 97 | 4 |
| B4 | 10 | 13 | 8 | 11 | 5 | 12 | 59 | 1 |
| B5 | 19 | 16 | 12 | 18 | 14 | 22 | 101 | 3 |
| B6 | 17 | 16 | 12 | 15 | 17 | 25 | 102 | 5 |
| B7 | 22 | 19 | 15 | 19 | 20 | 29 | 124 | 5 |
| *Smiling Mind M* | 17.71 | 16.57 | 12.00 | 16.14 | 14.57 | 23.00 | 100.00 | 3.86 |
| *Smiling Mind SD* | 3.99 | 1.90 | 2.08 | 2.61 | 4.86 | 5.60 | 20.12 | 1.46 |
| C1 | 19 | 16 | 14 | 18 | 13 | 24 | 104 | 4 |
| C2 | 20 | 17 | 13 | 17 | 16 | 25 | 108 | 4 |
| C3 | 14 | 12 | 9 | 17 | 7 | 22 | 81 | 3 |
| C4 | 22 | 16 | 14 | 18 | 13 | 25 | 108 | 4 |
| C5 | 22 | 16 | 13 | 17 | 13 | 25 | 106 | 3 |
| C6 | 14 | 13 | 10 | 15 | 10 | 20 | 82 | 3 |
| *MoodMission M* | 18.50 | 15.00 | 12.17 | 17.00 | 12.00 | 23.50 | 98.17 | 3.50 |
| *MoodMission SD* | 3.67 | 2.00 | 2.14 | 1.10 | 3.10 | 2.07 | 13.00 | 0.55 |
| D1 | 17 | 16 | 13 | 18 | 16 | 25 | 105 | 4 |
| D2 | 11 | 12 | 9 | 14 | 6 | 18 | 70 | 2 |
| D3 | 16 | 13 | 11 | 17 | 11 | 24 | 92 | 3 |
| D4 | 16 | 15 | 11 | 18 | 8 | 23 | 91 | 3 |
| D5 | 13 | 11 | 8 | 13 | 5 | 10 | 60 | 1 |
| *MindShift M* | 14.60 | 13.40 | 10.40 | 16.00 | 9.20 | 20.00 | 83.60 | 2.60 |
| *MindShift SD* | 2.51 | 2.07 | 1.95 | 2.35 | 4.44 | 6.21 | 18.20 | 1.14 |
| E1 | 16 | 16 | 10 | 15 | 15 | 22 | 94 | 4 |
| E2 | 16 | 14 | 9 | 15 | 8 | 22 | 84 | 3 |
| E3 | 15 | 16 | 8 | 13 | 8 | 17 | 77 | 3 |
| E4 | 15 | 15 | 9 | 16 | 7 | 18 | 80 | 3 |
| E5 | 10 | 12 | 7 | 12 | 5 | 10 | 56 | 2 |
| E6 | 19 | 15 | 13 | 17 | 14 | 24 | 102 | 3 |
| *Destressify M* | 15.17 | 14.67 | 9.33 | 14.67 | 9.50 | 18.83 | 82.17 | 3.00 |
| *Destressify SD* | 2.93 | 1.51 | 2.07 | 1.86 | 4.04 | 5.08 | 15.83 | 0.63 |

*Note*. Participants A1 – A5 = *SuperBetter*; B1 – B7 = *Smiling Mind*; C1 – C6 = *MoodMission*; D1 – D5 = *MindShift*; and E1 – E6 = *Destressify*.
